# Supplementary material for: Ongoing training of community health workers in low-income and middle-income countries: a systematic scoping review of the literature
Source: BMJ Open. 2018 Apr 28;8(4):e021467. doi: 10.1136/bmjopen-2017-021467 (PMC5931295; doi:10.1136/bmjopen-2017-021467)
Supplement: Supplementary file 1 [file bmjopen-2017-021467supp001.pdf]

Supplementary Material

Table 1. Database search strategies, including search terms.

Database(s)	Search strategy
**AMED** (Allied and Complementary Medicine) 1985 to July 2017, **Embase** 1974 to 2017 July 10, **Global Health** 1973 to 2017 Week 29 **Ovid MEDLINE(R)** 1946 to July Week 2 2017.	**1.** Developing Countries**.sh,kf.**<br>**2.** (Africa or Asia or Caribbean or West Indies or South America or Latin America or Central America)**.hw,kf,ti,ab,cp.**<br>**3.** (Afghanistan or Albania or Algeria or Angola or Antigua or Barbuda or Argentina or Armenia or Armenian or Aruba or Azerbaijan or Bahrain or Bangladesh or Barbados or Benin or Byelarus or Byelorussian or Belarus or Belorussian or Belorussia or Belize or Bhutan or Bolivia or Bosnia or Herzegovina or Hercegovina or Botswana or Brasil or Brazil or Bulgaria or Burkina Faso or Burkina Fasso or Upper Volta or Burundi or Urundi or Cambodia or Khmer Republic or Kampuchea or Cameroon or Cameroons or Cameron or Camerons or Cape Verde or Central African Republic or Chad or Chile or China or Colombia or Comoros or Comoro Islands or Comores or Mayotte or Congo or Zaire or Costa Rica or Cote d'Ivoire or Ivory Coast or Croatia or Cuba or Cyprus or Czechoslovakia or Czech Republic or Slovakia or Slovak Republic or Djibouti or French Somaliland or Dominica or Dominican Republic or East Timor or East Timur or Timor Leste or Ecuador or Egypt or United Arab Republic or El Salvador or Eritrea or Estonia or Ethiopia or Fiji or Gabon or Gabonese Republic or Gambia or Gaza or Georgia Republic or Georgian Republic or Ghana or Gold Coast or Greece or Grenada or Guatemala or Guinea or Guam or Guiana or Guyana or Haiti or Honduras or Hungary or India or Maldives or Indonesia or Iran or Iraq or Isle of Man or Jamaica or Jordan or Kazakhstan or Kazakh or Kenya or Kiribati or Korea or Kosovo or Kyrgyzstan or Kirghizia or Kyrgyz Republic or Kirghiz or Kirgizstan or Lao PDR or Laos or Latvia or Lebanon or Lesotho or Basutoland or Liberia or Libya or Lithuania or Macedonia or Madagascar or Malagasy Republic or Malaysia or Malaya or Malay or Sabah or Sarawak or Malawi or Nyasaland or Mali or Malta or Marshall Islands or Mauritania or Mauritius or Agalega Islands or Mexico or Micronesia or Middle East or Moldova or Moldovia or Moldovian or Mongolia or Montenegro or Morocco or Ifni or Mozambique or Myanmar or Myanma or Burma or Namibia or Nepal or Netherlands Antilles or New Caledonia or Nicaragua or Niger or Nigeria or Northern Mariana Islands or Oman or Muscat or Pakistan or Palau or Palestine or Panama or Paraguay or Peru or Philippines or

	Philipines or Phillipines or Phillippines or Poland or Portugal or Puerto Rico or Romania or Rumania or Roumania or Russia or Russian or Rwanda or Ruanda or Saint Kitts or St Kitts or Nevis or Saint Lucia or St Lucia or Saint Vincent or St Vincent or Grenadines or Samoa or Samoan Islands or Navigator Island or Navigator Islands or Sao Tome or Saudi Arabia or Senegal or Serbia or Montenegro or Seychelles or Sierra Leone or Slovenia or Sri Lanka or Ceylon or Solomon Islands or Somalia or South Africa or Sudan or Suriname or Surinam or Swaziland or Syria or Tajikistan or Tadzhikistan or Tadjikistan or Tadzhik or Tanzania or Thailand or Togo or Togolese Republic or Tonga or Trinidad or Tobago or Tunisia or Turkey or Turkmenistan or Turkmen or Uganda or Ukraine or Uruguay or USSR or Soviet Union or Union of Soviet Socialist Republics or Uzbekistan or Uzbek or Vanuatu or New Hebrides or Venezuela or Vietnam or Viet Nam or West Bank or Yemen or Yugoslavia or Zambia or Zimbabwe or Rhodesia)**.hw,kf,ti,ab,cp.**<br>**4.** ((developing or less* developed or under developed or underdeveloped or middle income or low* income or underserved or under served or deprived or poor*) adj (countr* or nation? or population? or world))**.ti,ab.**<br>**5.** ((developing or less* developed or under developed or underdeveloped or middle income or low* income) adj (economy or economies))**.ti,ab.**<br>**6.** (low* adj (gdp or gnp or gross domestic or gross national))**.ti,ab.**<br>**7.** (low adj3 middle adj3 countr*)**.ti,ab.**<br>**8.** (lmic or lmics or third world or lami countr*)**.ti,ab.**<br>**9.** transitional countr***.ti,ab.**<br>**10.** or/1-9<br>AND<br>11. ((refresher* adj (train* or course*)) or (adequa* adj2 train*) or (on-going training) or (on-going education) or (continuing education) or ((in-service or update or recap*) adj3 train*)) or (exp education, continuing/ or exp inservice training/) or (supervision) or (supportive supervis*)<br>AND<br>12. ((community adj health* adj3 (worker* or volunteer or aide* or practition*)) or (community adj (mental health*) adj3 (worker* or volunteer or aide*)) or (village adj health* adj (worker* or team* or guide*)) or (lady health worker*) or (lady health visitor*) or (front-line primary health?care) or (front-line primary health care) or behvarz or brigadista or manzaneras or (rural health assistant*) or gramsakhi or (lay health worker*) or (trained birth assistant*) or (accredited social health activist*) or (adherence support worker*) or (care facilitator*) or (community adj10 (treatment support*)) or (community* adj4 (distributor* or volunteer*)) or (health extension worker*) or (lay counsellor*) or (maternal health

	worker*) or (shasthy? shebikas) or (shasthy? kormis) or (front line primary health* care worker) or (front line primary healthcare worker*) or (health activist*)).ti,ab.
**SCOPUS**	(TITLE-ABS-KEY ((front-line AND primary AND healthcare) OR (front-line AND primary AND health AND care) OR behvarz OR brigadista OR manzaneras OR (rural AND health AND assistant*) OR gramsakhi OR (lay AND health AND worker*) OR (trained AND birth AND assistant*) OR (accredited AND social AND health AND activist*) OR (adherence AND support AND worker*) OR (care AND facilitator*))) OR (TITLE-ABS-KEY ((community AND health* W/3 (worker* OR volunteer OR aide* OR practit*)) OR (community AND mental AND health* W/3 (worker* OR volunteer OR aide* OR practit*)) OR (village AND health* W/1 (worker* OR team* OR guide*)) OR (lady AND health AND worker*) OR (lady AND health AND visitor*) OR (lay AND healthworker) OR (TITLE-ABS-KEY ((health AND extension AND worker*) OR (lay AND counsellor*) OR (maternal AND health AND worker*) OR (peer AND educator*) OR (shasthy* AND shebikas) OR (shasthy* AND kormi) OR (front AND line AND primary AND health* AND care AND worker) OR (front AND line AND primary AND healthcare AND worker*) OR (health AND activist*))) OR (TITLE-ABS-KEY (community W/10 treatment AND support*)) OR (TITLE-ABS-KEY (community W/10 distributor*)) OR (TITLE-ABS-KEY (community W/10 volunteer*)) OR (TITLE-ABS-KEY (community W/10 care AND worker*)) OR (TITLE-ABS-KEY (community W/10 health AND worker*))<br><br>**AND**<br>(TITLE-ABS-KEY (refresher AND train* OR refresher AND course* OR (adequa* W/2 train*) OR (on-going education) OR (on-going training) OR (continuing education) OR (continuing training) OR (recap training) OR (in-service training) OR (update training) OR (supervision) or (supportive supervision)<br><br>**Limits applied**

	1. Countries - (Afghanistan or Albania or Algeria or Angola or Antigua or Barbuda or Argentina or Armenia or Armenian or Aruba or Azerbaijan or Bahrain or Bangladesh or Barbados or Benin or Byelarus or Byelorussian or Belarus or Belorussian or Belorussia or Belize or Bhutan or Bolivia or Bosnia or Herzegovina or Hercegovina or Botswana or Brasil or Brazil or Bulgaria or Burkina Faso or Burkina Fasso or Upper Volta or Burundi or Urundi or Cambodia or Khmer Republic or Kampuchea or Cameroon or Cameroons or Cameron or Camerons or Cape Verde or Central African Republic or Chad or Chile or China or Colombia or Comoros or Comoro Islands or Comores or Mayotte or Congo or Zaire or Costa Rica or Cote d'Ivoire or Ivory Coast or Croatia or Cuba or Cyprus or Czechoslovakia or Czech Republic or Slovakia or Slovak Republic or Djibouti or French Somaliland or Dominica or Dominican Republic or East Timor or East Timur or Timor Leste or Ecuador or Egypt or United Arab Republic or El Salvador or Eritrea or Estonia or Ethiopia or Fiji or Gabon or Gabonese Republic or Gambia or Gaza or Georgia Republic or Georgian Republic or Ghana or Gold Coast or Greece or Grenada or Guatemala or Guinea or Guam or Guiana or Guyana or Haiti or Honduras or Hungary or India or Maldives or Indonesia or Iran or Iraq or Isle of Man or Jamaica or Jordan or Kazakhstan or Kazakh or Kenya or Kiribati or Korea or Kosovo or Kyrgyzstan or Kirghizia or Kyrgyz Republic or Kirghiz or Kirgizstan or Lao PDR or Laos or Latvia or Lebanon or Lesotho or Basutoland or Liberia or Libya or Lithuania or Macedonia or Madagascar or Malagasy Republic or Malaysia or Malaya or Malay or Sabah or Sarawak or Malawi or Nyasaland or Mali or Malta or Marshall Islands or Mauritania or Mauritius or Agalega Islands or Mexico or Micronesia or Middle East or Moldova or Moldovia or Moldovian or Mongolia or Montenegro or Morocco or Ifni or Mozambique or Myanmar or Myanma or Burma or Namibia or Nepal or Netherlands Antilles or New Caledonia or Nicaragua or Niger or Nigeria or Northern Mariana Islands or Oman or Muscat or Pakistan or Palau or Palestine or Panama or Paraguay or Peru or Philippines or Philipines or Phillipines or Phillippines or Poland or Portugal or Puerto Rico or Romania or Rumania or Roumania or Russia or Russian or Rwanda or Ruanda or Saint Kitts or St Kitts or Nevis or Saint Lucia or St Lucia or Saint Vincent or St Vincent or Grenadines or Samoa or Samoan Islands

	or Navigator Island or Navigator Islands or Sao Tome or Saudi Arabia or Senegal or Serbia or Montenegro or Seychelles or Sierra Leone or Slovenia or Sri Lanka or Ceylon or Solomon Islands or Somalia or South Africa or Sudan or Suriname or Surinam or Swaziland or Syria or Tajikistan or Tadzhikistan or Tadjikistan or Tadzhik or Tanzania or Thailand or Togo or Togolese Republic or Tonga or Trinidad or Tobago or Tunisia or Turkey or Turkmenistan or Turkmen or Uganda or Ukraine or Uruguay or USSR or Soviet Union or Union of Soviet Socialist Republics or Uzbekistan or Uzbek or Vanuatu or New Hebrides or Venezuela or Vietnam or Viet Nam or West Bank or Yemen or Yugoslavia or Zambia or Zimbabwe or Rhodesia)<br>2. Dates - (1978-2017)

**Web of Science**	# 25 - #24 AND #16 AND #15<br>Indexes=SCI-EXPANDED, SSCI, A&HCI, CPCI-S, CPCI-SSH, BKCI-S, BKCI-SSH, ESCI, CCR-EXPANDED, IC Timespan=1978-2017<br># 24 - #23 OR #22 OR #21 OR #20 OR #19 OR #18 OR #17<br>Indexes=SCI-EXPANDED, SSCI, A&HCI, CPCI-S, CPCI-SSH, BKCI-S, BKCI-SSH, ESCI, CCR-EXPANDED, IC Timespan=1978-2017<br># 23 - TS=((health extension worker*) or (lay counsellor*) or (maternal health worker*) or (shasthy* kormis) or (shasthy* shebikas) or (health activist*))<br>Indexes=SCI-EXPANDED, SSCI, A&HCI, CPCI-S, CPCI-SSH, BKCI-S, BKCI-SSH, ESCI, CCR-EXPANDED, IC Timespan=1978-2017<br># 22 - TS=(community SAME treatment support*) OR TS=(community* NEAR/4 distributor*) OR TS=(community* NEAR/4 volunteer*) OR TS= (community* NEAR/4 care worker*) OR TS=(community* NEAR/4 careworker*) OR TS=(community* NEAR/4 healthworker*) OR TS=(community* NEAR/4 health worker*)<br>Indexes=SCI-EXPANDED, SSCI, A&HCI, CPCI-S, CPCI-SSH, BKCI-S, BKCI-SSH, ESCI, CCR-EXPANDED, IC Timespan=1978-2017<br># 21 - TS=((accredited social health activist*) or (adherence support worker*) or (care facilitator*))<br>Indexes=SCI-EXPANDED, SSCI, A&HCI, CPCI-S, CPCI-SSH, BKCI-S, BKCI-SSH, ESCI, CCR-EXPANDED, IC Timespan=1978-2017<br># 20 - TS=(behvarz or brigadista or manzaneras or (rural health assistant*) or gramsakhi or (lay health worker*) or (trained birth assistant*))<br>Indexes=SCI-EXPANDED, SSCI, A&HCI, CPCI-S, CPCI-SSH, BKCI-S, BKCI-SSH, ESCI, CCR-EXPANDED, IC Timespan=1978-2017<br># 19 - Ts=(frontline primary healthcare*) OR TS=(front line primary healthcare*) OR TS=(frontline primary health care*) OR TS=(front line primary health care*)<br>Indexes=SCI-EXPANDED, SSCI, A&HCI, CPCI-S, CPCI-SSH, BKCI-S, BKCI-SSH, ESCI, CCR-EXPANDED, IC Timespan=1978-2017<br># 18 - TS=(village health* worker*) OR TS=(village health* guide*) OR TS=(village health* team) OR TS=(lady health worker*)<br>Indexes=SCI-EXPANDED, SSCI, A&HCI, CPCI-S, CPCI-SSH, BKCI-S, BKCI-SSH, ESCI, CCR-EXPANDED, IC Timespan=1978-2017<br># 17 - TS=(community health* NEAR/3 worker*) OR TS=(community health* NEAR/3 volunteer*) OR TS=(community health* NEAR/3 aide*) OR TS=(community health* NEAR/3 practit*) OR TS=(community mental health* NEAR/3 worker*) OR TS=(community mental health* NEAR/3 volunteer*) OR TS=(community mental health* NEAR/3 aide*)<br>Indexes=SCI-EXPANDED, SSCI, A&HCI, CPCI-S, CPCI-SSH, BKCI-S, BKCI-SSH, ESCI, CCR-EXPANDED, IC Timespan=1978-2017

	# 16 98,552<br>#14 OR #13 OR #12<br>Indexes=SCI-EXPANDED, SSCI, A&HCI, CPCI-S, CPCI-SSH, BKCI-S, BKCI-SSH, ESCI, CCR-EXPANDED, IC<br>Timespan=1978-2017<br># 15 3,687,339<br>#11 OR #10 OR #9 OR #8 OR #7 OR #6 OR #5 OR #4 OR #3 OR #2 OR #1<br>Indexes=SCI-EXPANDED, SSCI, A&HCI, CPCI-S, CPCI-SSH, BKCI-S, BKCI-SSH, ESCI, CCR-EXPANDED, IC<br>Timespan=1978-2017<br># 14 42,703<br>TS=(recap* NEAR/3 train*) OR TS=(update NEAR/3 train*) OR TS=(ongoing NEAR/3 train*) OR TS=(in-service NEAR/3 train*) OR TS=(on-going education) OR TS=(supervision) OR TS=(supportive supervision)<br>Indexes=SCI-EXPANDED, SSCI, A&HCI, CPCI-S, CPCI-SSH, BKCI-S, BKCI-SSH, ESCI, CCR-EXPANDED, IC<br>Timespan=1978-2017<br># 13 53,039<br>TS=(continuing education) OR TS=(continuing training)<br>Indexes=SCI-EXPANDED, SSCI, A&HCI, CPCI-S, CPCI-SSH, BKCI-S, BKCI-SSH, ESCI, CCR-EXPANDED, IC<br>Timespan=1978-2017<br># 12 4,506<br>TS=(refresher train* or refresher course*) OR TS=(adequa* NEAR/2 train*)<br>Indexes=SCI-EXPANDED, SSCI, A&HCI, CPCI-S, CPCI-SSH, BKCI-S, BKCI-SSH, ESCI, CCR-EXPANDED, IC<br>Timespan=1978-2017<br># 11 141,978<br>TS=(underserved population* or underserved world* or underserved countr* or underserved nation*) OR TS=(under served population* or under served world* or under served countr* or under served nation*) OR TS=(deprived population* or deprived world* or deprived countr* or deprived nation*) OR TS=(poor population* or poor world* or poorcountr* or poor nation*)<br>Indexes=SCI-EXPANDED, SSCI, A&HCI, CPCI-S, CPCI-SSH, BKCI-S, BKCI-SSH, ESCI, CCR-EXPANDED, IC<br>Timespan=1978-2017<br># 10 438,437<br>TS=(developing population* or developing world*) OR TS=(less developed population* or less developed world*) OR TS=(under developed population* or under developed world*) OR TS=(underdeveloped population* or underdeveloped world*) OR TS=(middle income population* or middle income world*) OR TS=(low* income population* or low* income world*)<br>Indexes=SCI-EXPANDED, SSCI, A&HCI, CPCI-S, CPCI-SSH, BKCI-S, BKCI-SSH, ESCI, CCR-EXPANDED, IC<br>Timespan=1978-2017<br># 9 354,673<br>TS=(developing countr* or developing nation*) OR TS=(less developed countr* or less developed nation*) OR TS=(under developed countr* or under developed nation*) OR

	TS=(underdeveloped countr* or underdeveloped nation*) OR<br>TS=(middle income countr* or middle income nation*) OR<br>TS=(low* income countr* or low* income nation*)<br>Indexes=SCI-EXPANDED, SSCI, A&HCI, CPCI-S, CPCI-SSH, BKCI-S, BKCI-SSH, ESCI, CCR-EXPANDED, IC<br>Timespan=1978-2017<br># 8 50,977<br>TS=(developing economies or developing economy) OR TS=(less developed economies or less developed economy) OR TS=(under developed economies or under developed economy) OR<br>TS=(underdeveloped economies or underdeveloped economy) OR<br>TS=(middle income economies or middle income economies) OR<br>TS=(low* income economies or low* income economy)<br>Indexes=SCI-EXPANDED, SSCI, A&HCI, CPCI-S, CPCI-SSH, BKCI-S, BKCI-SSH, ESCI, CCR-EXPANDED, IC<br>Timespan=1978-2017<br># 7 7,542<br>TS=(low* gdp or low* GNP or low* gross domestic or low* gross national)<br>Indexes=SCI-EXPANDED, SSCI, A&HCI, CPCI-S, CPCI-SSH, BKCI-S, BKCI-SSH, ESCI, CCR-EXPANDED, IC<br>Timespan=1978-2017<br># 6 15,495<br>TS=(low SAME middle SAME countr*)<br>Indexes=SCI-EXPANDED, SSCI, A&HCI, CPCI-S, CPCI-SSH, BKCI-S, BKCI-SSH, ESCI, CCR-EXPANDED, IC<br>Timespan=1978-2017<br># 5 32,915<br>TS= (lmic or lmics or third world or lami countr*)<br>Indexes=SCI-EXPANDED, SSCI, A&HCI, CPCI-S, CPCI-SSH, BKCI-S, BKCI-SSH, ESCI, CCR-EXPANDED, IC<br>Timespan=1978-2017<br># 4 2,471<br>TS=(transitional countr*)<br>Indexes=SCI-EXPANDED, SSCI, A&HCI, CPCI-S, CPCI-SSH, BKCI-S, BKCI-SSH, ESCI, CCR-EXPANDED, IC<br>Timespan=1978-2017<br># 2 522,425<br>TS=(Africa or Asia or Caribbean or West Indies or South America or Latin America or Central America)<br>Indexes=SCI-EXPANDED, SSCI, A&HCI, CPCI-S, CPCI-SSH, BKCI-S, BKCI-SSH, ESCI, CCR-EXPANDED, IC<br>Timespan=1978-2017<br># 1 213,604<br>TS=Developing countries<br>Indexes=SCI-EXPANDED, SSCI, A&HCI, CPCI-S, CPCI-SSH, BKCI-S, BKCI-SSH, ESCI, CCR-EXPANDED, IC<br>Timespan=1978-2017

**ASSIA**	ab(((community health* worker* or community health volunteer or or community health aide* or community health practitioner or community mental health* worker* or community mental health* volunteer or community mental health* aide* or village health* worker* or village health* team* or village health* guide* or lady health worker* or lady health visitor* or laywomen* or laywoman* or front-line primary healthcare or front-line primary health care or behvarz or brigadista or manzaneras or rural health assistant* or gramsakhi or lay health worker* or trained birth assistant* or accredited social health activist* or adherence support worker* or care facilitator* or (community and (treatment support*)) or (community* and (distributor* or volunteer* or care worker* or health worker)) or health extension worker* or lay counsellor* or maternal health worker* or peer educator* or shasthya shebikas or shasthya kormis or front line primary health* care worker or front line primary healthcare worker* or health activist*) AND (refresher* train* or refresher* course* or (adequa* and train*) or (continuing medical education) or (continuing medical training) or (continuing nursing education) or (continuing nursing training) or or (supervision) or (supportive supervision) or (continuing education) or ((in-service or ongoing or update or recap*) and train*)) AND (developing countries or Africa or Asia or Caribbean or West Indies or South America or Latin America or Central America or lmic or underdeveloped countries or middle income countries or low income countries or transitional countries))) OR ti((community health* worker* OR community health volunteer OR community health aide* OR community mental health* worker* OR community mental health* volunteer OR community mental health* aide* OR village health* worker* OR village health* team* OR village health* guide* OR lady health worker* OR lady health visitor* OR laywomen* OR laywoman* OR front-line primary healthcare OR front-line primary health care OR behvarz OR brigadista OR manzaneras OR rural health assistant* OR gramsakhi OR lay health worker* OR trained birth assistant* OR accredited social health activist* OR adherence support worker* OR care facilitator* OR (community AND (treatment support*)) OR (community* AND (distributor* OR volunteer* OR care worker* OR health worker)) OR health extension worker* OR lay counsellor* OR maternal health worker* OR peer educator* OR shasthy* shebikas OR shasthy* kormis OR front line primary health* care worker OR front line primary healthcare worker* OR health activist*) AND (refresher* train* OR refresher* course* OR (adequa* AND train*) OR (continuing medical education) OR (continuing medical training) OR (continuing nursing education) OR (continuing

	nursing training) OR (continuing education) OR ((in-service OR ongoing OR update OR recap*) AND train*)) AND (developing countries OR Africa OR Asia OR Caribbean OR West Indies OR South America OR Latin America OR Central America OR lmic OR underdeveloped countries OR middle income countries OR low income countries OR transitional countries))
**LILACS**	community health workers 7<br>community health volunteer 0<br>community health aide 2<br>community mental health worker 0<br>community mental health volunteer 0<br>community mental health aide or 0<br>village health worker 1<br>village health team 0<br>village health guide 0<br>lady health worker 0<br>lady health visitor 0<br>laywomen 0<br>laywoman 0<br>front-line primary healthcare 0<br>front-line primary health care 0<br>behvarz 0<br>brigadista 0<br>manzaneras or<br>rural health assistant* 0<br>gramsakhi 0<br>lay health worker 0<br>trained birth assistant 0<br>accredited social health activist 0<br>adherence support worker 0<br>care facilitator 0<br>treatment support 11<br>community 0<br>health extension worker 0<br>lay counsellor 0<br>maternal health worker 0<br>peer educator 0<br>shasthya shebikas 0<br>shasthya kormis 0<br>health activist 0<br><br>and<br><br>refresher training or refresher course or adequate training or adequately trained or on-going education or on-going training or continuing education or in-service training or update training or recap or supervision or supportive

	supervision
**British Educational Index, ERIC via EBSCO**	S13 S10 AND S11 AND S12<br>S12 TI (((community N1 health* N3 (worker* or volunteer or aide* or practition*)) or (community N1 (mental health* N3 (worker* or volunteer or aide*)) or (village N1 health* N1 (worker* or team* or guide*)) or (lady health worker*) or (lady health visitor*) or (laywomen* or laywoman*) or (front-line primary healthcare) or (front-line primary health care) or behvarz or brigadista or manzaneras or (rural health assistant*) or gramsakhi or (lay health worker*) or (trained birth assistant*) or (accredited social health activist*) or (adherence support worker*) or (care facilitator*) or (community N10 (treatment support*)) or (community* N4 (distributor* or volunteer* or (care worker*) or (health worker))) or (health extension worker*) or (lay counsellor*) or (maternal health worker*) or (peer educator*) or (shasthya shebikas) or (shasthya kormis) or (front line primary health* care worker) or (front line primary healthcare worker*) or (health activist*))) OR AB (((community N1 health* N3 (worker* or volunteer or aide*)) or (community N1 (mental health* N3 (worker* or volunteer or aide*)) or (village N1 health* N1 (worker* or team* or guide*)) or (lady health worker*) or (lady health visitor*) or (laywomen* or laywoman*) or (front-line primary healthcare) or (front-line primary health care) or behvarz or brigadista or manzaneras or (rural health assistant*) or gramsakhi or (lay health worker*) or (trained birth assistant*) or (accredited social health activist*) or (adherence support worker*) or (care facilitator*) or (community N10 (treatment support*)) or (community* N4 (distributor* or volunteer* or (care worker*) or (health worker))) or (health extension worker*) or (lay counsellor*) or (maternal health worker*) or (shasthy* shebikas) or (shasthy* korbis) or (front line primary health* care worker) or (front line primary healthcare worker*) or (health activist*)))<br>S11 TI (((refresher* N1 (train* or course*)) or (adequa* N2 train*) or (on?going education) or (on?going training) or (continuing education) or (supervision) or (supportive supervision) or ((in-service or ongoing or update or recap*) N3 train*))) OR AB (((refresher* N1 (train* or course*)) or (adequa* N2 train*) or (on?going education) or (on?going training) or (supervision) or (supportive supervis*) or ((in-service or ongoing or update or recap*) N3 train*)))<br>S10 S1 OR S2 OR S3 OR S4 OR S5 OR S6 OR S7 OR S8 OR S9<br>S9 TI transitional countr* OR AB transitional countr*<br>S8 TI (lmic or lmics or third world or lami countr*)) OR AB (lmic or lmics or third world or lami countr*))<br>S7 TI (low N3 middle N3 countr*) OR AB (low N3 middle N3 countr*)<br>S6 TI ((low* N1 (gdp or gnp or gross domestic or gross national))) OR AB ((low* N1 (gdp or gnp or gross domestic or gross national)))<br>S5 TI (((developing or less* developed or under developed or underdeveloped or middle income or low* income) N1 (economy

	or economies))) OR AB (((developing or less* developed or under developed or underdeveloped or middle income or low* income) N1 (economy or economies)))<br>S4 TI (((developing or less* developed or under developed or underdeveloped or middle income or low* income or underserved or under served or deprived or poor*) N1 (countr* or nation* or population* or world))) OR AB (((developing or less* developed or under developed or underdeveloped or middle income or low* income or underserved or under served or deprived or poor*) N1 (countr* or nation* or population* or world)))<br>S3 TI (Afghanistan or Albania or Algeria or Angola or Antigua or Barbuda or Argentina or Armenia or Armenian or Aruba or Azerbaijan or Bahrain or Bangladesh or Barbados or Benin or Byelarus or Byelorussian or Belarus or Belorussian or Belorussia or Belize or Bhutan or Bolivia or Bosnia or Herzegovina or Hercegovina or Botswana or Brasil or Brazil or Bulgaria or Burkina Faso or Burkina Fasso or Upper Volta or Burundi or Urundi or Cambodia or Khmer Republic or Kampuchea or Cameroon or Cameroons or Cameron or Camerons or Cape Verde or Central African Republic or Chad or Chile or China or Colombia or Comoros or Comoro Islands or Comores or Mayotte or Congo or Zaire or Costa Rica or Cote d'Ivoire or Ivory Coast or Croatia or Cuba or Cyprus or Czechoslovakia or Czech Republic or Slovakia or Slovak Republic or Djibouti or French Somaliland or Dominica or Dominican Republic or East Timor or East Timur or Timor Leste or Ecuador or Egypt or United Arab Republic or El Salvador or Eritrea or Estonia or Ethiopia or Fiji or Gabon or Gabonese Republic or Gambia or Gaza or Georgia Republic or Georgian Republic or Ghana or Gold Coast or Greece or Grenada or Guatemala or Guinea or Guam or Guiana or Guyana or Haiti or Honduras or Hungary or India or Maldives or Indonesia or Iran or Iraq or Isle of Man or Jamaica or Jordan or Kazakhstan or Kazakh or Kenya or Kiribati or Korea or Kosovo or Kyrgyzstan or Kirghizia or Kyrgyz Republic or Kirghiz or Kirgizstan or Lao PDR or Laos or Latvia or Lebanon or Lesotho or Basutoland or Liberia or Libya or Lithuania or Macedonia or Madagascar or Malagasy Republic or Malaysia or Malaya or Malay or Sabah or Sarawak or Malawi or Nyasaland or Mali or Malta or Marshall Islands or Mauritania or Mauritius or Agalega Islands or Mexico or Micronesia or Middle East or Moldova or Moldovia or Moldovian or Mongolia or Montenegro or Morocco or Ifni or Mozambique or Myanmar or Myanma or Burma or Namibia or Nepal or Netherlands Antilles or New Caledonia or Nicaragua or Niger or Nigeria or Northern Mariana Islands or Oman or Muscat or Pakistan or Palau or Palestine or Panama or Paraguay or Peru or Philippines or Philipines or Phillipines or Phillippines or Poland or Portugal or Puerto Rico or Romania or Rumania or Roumania or Russia or Russian or Rwanda or Ruanda or Saint Kitts or St Kitts or Nevis or Saint Lucia or St Lucia or Saint Vincent or St Vincent or Grenadines or Samoa or Samoan Islands or Navigator Island or Navigator Islands or Sao Tome or Saudi Arabia or Senegal or Serbia or Montenegro or Seychelles or Sierra Leone or Slovenia or Sri Lanka or Ceylon or Solomon Islands or Somalia or South Africa or Sudan or Suriname or Surinam or Swaziland or Syria or Tajikistan or Tadzhikistan or Tadjikistan or Tadzhik or Tanzania

	or Thailand or Togo or Togolese Republic or Tonga or Trinidad or Tobago or Tunisia or Turkey or Turkmenistan or Turkmen or Uganda or Ukraine or Uruguay or USSR or Soviet Union or Union of Soviet Socialist Republics or Uzbekistan or Uzbek or Vanuatu or New Hebrides or Venezuela or Vietnam or Viet Nam or West Bank or Yemen or Yugoslavia or Zambia or Zimbabwe or Rhodesia) OR AB (Afghanistan or Albania or Algeria or Angola or Antigua or Barbuda or Argentina or Armenia or Armenian or Aruba or Azerbaijan or Bahrain or Bangladesh or Barbados or Benin or Byelarus or Byelorussian or Belarus or Belorussian or Belorussia or Belize or Bhutan or Bolivia or Bosnia or Herzegovina or Hercegovina or Botswana or Brasil or Brazil or Bulgaria or Burkina Faso or Burkina Fasso or Upper Volta or Burundi or Urundi or Cambodia or Khmer Republic or Kampuchea or Cameroon or Cameroons or Cameron or Camerons or Cape Verde or Central African Republic or Chad or Chile or China or Colombia or Comoros or Comoro Islands or Comores or Mayotte or Congo or Zaire or Costa Rica or Cote d'Ivoire or Ivory Coast or Croatia or Cuba or Cyprus or Czechoslovakia or Czech Republic or Slovakia or Slovak Republic or Djibouti or French Somaliland or Dominica or Dominican Republic or East Timor or East Timur or Timor Leste or Ecuador or Egypt or United Arab Republic or El Salvador or Eritrea or Estonia or Ethiopia or Fiji or Gabon or Gabonese Republic or Gambia or Gaza or Georgia Republic or Georgian Republic or Ghana or Gold Coast or Greece or Grenada or Guatemala or Guinea or Guam or Guiana or Guyana or Haiti or Honduras or Hungary or India or Maldives or Indonesia or Iran or Iraq or Isle of Man or Jamaica or Jordan or Kazakhstan or Kazakh or Kenya or Kiribati or Korea or Kosovo or Kyrgyzstan or Kirghizia or Kyrgyz Republic or Kirghiz or Kirgizstan or Lao PDR or Laos or Latvia or Lebanon or Lesotho or Basutoland or Liberia or Libya or Lithuania or Macedonia or Madagascar or Malagasy Republic or Malaysia or Malaya or Malay or Sabah or Sarawak or Malawi or Nyasaland or Mali or Malta or Marshall Islands or Mauritania or Mauritius or Agalega Islands or Mexico or Micronesia or Middle East or Moldova or Moldovia or Moldovian or Mongolia or Montenegro or Morocco or Ifni or Mozambique or Myanmar or Myanma or Burma or Namibia or Nepal or Netherlands Antilles or New Caledonia or Nicaragua or Niger or Nigeria or Northern Mariana Islands or Oman or Muscat or Pakistan or Palau or Palestine or Panama or Paraguay or Peru or Philippines or Philipines or Phillipines or Phillippines or Poland or Portugal or Puerto Rico or Romania or Rumania or Roumania or Russia or Russian or Rwanda or Ruanda or Saint Kitts or St Kitts or Nevis or Saint Lucia or St Lucia or Saint Vincent or St Vincent or Grenadines or Samoa or Samoan Islands or Navigator Island or Navigator Islands or Sao Tome or Saudi Arabia or Senegal or Serbia or Montenegro or Seychelles or Sierra Leone or Slovenia or Sri Lanka or Ceylon or Solomon Islands or Somalia or South Africa or Sudan or Suriname or Surinam or Swaziland or Syria or Tajikistan or Tadzhikistan or Tadjikistan or Tadzhik or Tanzania or Thailand or Togo or Togolese Republic or Tonga or Trinidad or Tobago or Tunisia or Turkey or Turkmenistan or Turkmen or Uganda or Ukraine or Uruguay or USSR or Soviet Union or Union of Soviet Socialist Republics or Uzbekistan or Uzbek or Vanuatu

	or New Hebrides or Venezuela or Vietnam or Viet Nam or West Bank or Yemen or Yugoslavia or Zambia or Zimbabwe or Rhodesia)<br>S2 SU (Africa or Asia or Caribbean or West Indies or South America or Latin America or Central America) OR MW (Africa or Asia or Caribbean or West Indies or South America or Latin America or Central America) OR MM (Africa or Asia or Caribbean or West Indies or South America or Latin America or Central America) OR MJ (Africa or Asia or Caribbean or West Indies or South America or Latin America or Central America) OR TI (Africa or Asia or Caribbean or West Indies or South America or Latin America or Central America) OR AB (Africa or Asia or Caribbean or West Indies or South America or Latin America or Central America)<br>S1 SU Developing Countries OR MW Developing Countries

Table 2. Results from individual database searches.

**Database**	**Number of hits**	**Page number for search strategy details**
Medline (via OVID)	391	2-4
EMBASE (via OVID)	505	2-4
Global Health (via OVID)	351	2-4
AMED (via OVID)	36	2-4
Scopus	1217	5-6
Web of Science	1001	7-9
ASSIA via ProQuest	64	10
LILACS	21	11-12
BEI via EBSCO	38	13-16
ERIC via EBSCO	262	13-16
CINAHL	67	13-16

Legend. The results from individual database searches, including the number of hits and the supplementary material page numbers where the search strategies can be found.

Table 3. Details of included studies.

Authors	Study Title	Year	Country and Region	CHW name	Cadre description	Number of CHWs	Disease Focus Area	Training details	Outcome measure and outcomes	Use of mHealth
Adejumo et al.[34]	Community referral for presumptive TB in Nigeria: a comparison of four models of active case finding.	2016	Nigeria, West Africa	Community Workers (CWs)	Cadre description varied depending on the district from:<br><br>A) Unsupervised volunteer CWs where selection criteria was "any interested member of the community". Paid $13-20 quarterly.<br><br>to<br><br>B) 'Direct dealing CWs' whose selection criteria was that they had to:<br>1. Be known to the local leader;	124	TB	**Type:** Supervision **Content:** No details **Duration:** Variable number of supervisory visits, ranging from no supervision to three monthly depending on the model of supervision. **Provider:** TB Local Government Supervisors and community based partner organisations **Location:** Varying from no supervision to monthly meetings in the office and	**Outcome measure(s):** Change in behaviour, attitude or practice e.g. number of cases of TB detected in the community.<br><br>**Outcome(s):** The highest median referrals and mean TB diagnoses was obtained by the model with training supervision, and $80/quarterly payments (Comprehensive Quotas-Oriented model). The model with irregularly supervised, trained, and compensated	No details

					community;<br>4. Preferably have previous active involvement in volunteer work. Paid $80 quarterly.					
Ameha et al.[35]	Effectiveness of supportive supervision on the consistency of integrated community cases management skills of the health extension workers in 113 districts of Ethiopia.	2013	Ethiopia, East Africa	Health Extension Workers (HEWs)	No details provided	5000	Child Health	**Type:** Supervision<br>**Content:** Review of at least two cases from register and performance coaching<br>**Duration:** Variable number of supervisory visits -minimum of one, maximum of four.<br>**Provider:** John Snow, Inc. through the Last Ten Kilometers project (L10K) in partnership with the Ministry of Health<br>**Location:** Health posts	**Outcome measure(s):** Change in behaviour, attitude or practice e.g. number of recorded cases of diahorrea, malaria and pneumonia managed correctly in the community<br><br>**Outcome(s):** After controlling for secular trend and other factors, significant dose-response relationships were observed between number of supportive supervision visits and Integrated Community Case Management (iCCM) treatment indictors	No details
Ayele et al.[36]	The functional status of community	1993	Ethiopia, East Africa	Community Health Agents (CHAs)	No details provided	102	General focus	**Type:** Refresher training course and supervision.<br>**Content:** No	**Outcome measure(s):** Change in behaviour, attitude	No details

	health agents: A trial of refresher courses and regular supervision.							details **Duration:** Five-day refresher course and one supervision per month. **Provider:** Community leaders **Location:** In the community (field supervision)	or practice e.g. number of home visits, registration activities<br><br>**Outcome(s):** 10 out of the 13 CHA activity scores were higher in the group receiving refresher training and supervision at 3 and 6 months compared to the group not receiving it	
Carlough & McCall[37]	Skilled birth attendance: What does it mean and how can it be measured? A clinical skills assessment of maternal and child health workers in Nepal.	2005	Nepal, South Asia	Maternal and Child Health Workers (MCHWs)	Local women aged 18—35 who have completed a 15-week course in maternal and child health which covers both theoretical and practical components	104 (66 received refresher training)	Maternal and Reproductive Health	**Type:** Refresher training course**.** **Content:** Midwifery and emergency obstetric skills including focused antenatal care, active management of the third stage of labor, initial care for postpartum hemorrhage, pre-eclampsia and infection, and immediate neonatal care. MCHWs who completed refresher training receiving a first aid obstetric	**Outcome measure(s):** Mixed methods. Knowledge and skills assessment using a Clinical Skills Assessment (CSA) tool, plus a qualitative self-assessment scale.<br><br>**Outcome(s):** The MCHWs who received refresher training performed significantly better than those who did not on the CSA especially in the domains of: Use of medications in pregnancy; Managing post-partum	No details

								emergency kit **Duration:** Six-week refresher training course. **Provider:** No details **Location:** No details	hemorrhage; Normal delivery management	
Das et al.[38]	Strengthening malaria service delivery through supportive supervision and community mobilization in an endemic Indian setting: an evaluation of nested delivery models	2015	India, South Asia	Accredited social health activists (ASHAs)	The ASHAs role is in the "early detection, management and prevention of malaria at the community level They have been trained to test for malaria cases using rapid diagnostic tests and to treat these cases with artemisinin combination therapy.	N/A (randomised at village level)	Infectious disease	**Type:** Supervision **Content:** Recapping knowledge about transmission, diagnosis and treatment of malaria; practical support for performing and interpreting rapid diagnosis tests; administration of the correct dosage of ACT and follow-up to monitor compliance and record keeping **Duration:** Twice monthly. **Provider:** A mixture between the governments National Vector Borne Disease Control Programme and an NGO **Location:** In the	**Outcome measure(s):** Change in behaviour, attitude or practice at the household level e.g. Assessing for increased use of long last insecticide treated bed nets and proportion of cases tested for falciparum malaria within 24 hours. **Outcome(s):** Combining supportive supervision of CHWs with community mobilisation resulted in greater usage of bed nets and greater likelihood to seek treatment from a CHW resulting in fever cases being more likely to receive a timely	No details

								field	diagnosis	
Datiko et al.[39]	Exploring providers' perspectives of a community based TB approach in Southern Ethiopia: implication for community based approaches.	2015	Ethiopia, East Africa	Health Extension Workers (HEWs)/Community Health Promoters (CHPs)	HEWs trained for 1 year; salaried members of formal health system; range of duties. In this project their role was to collect sputum, produce smears and support patient treatment.<br><br>CHPs were unpaid volunteers, selected by communities to play a support role to HEWs. Their role was to support the HEW in identifying TB cases.	20	TB	**Type:** Supervision<br>**Content:** Supervising HEWs practically and ensuring smooth running of the project e.g. collection of sputum, case identification etc.<br>**Duration:** Twice monthly<br>**Provider:** District field supervisors funded by TB Reach (a multilateral funding organisation)<br>**Location:** In the field	**Outcome measure(s):** Qualitative assessment. Interviews and focus discussion groups to elicit the experiences of providers.<br><br>**Outcome(s):** HEWs felt generally well supported by their supervisors.<br>A small number of HEWs referred to supervisors who had not fulfilled the demanding co-ordination role well.<br>The greatest challenge for supervisors was the intensity of their workload coupled with the need to cover the large geographical area of their district.	No details
Dewing et al.[40]	Lay Counselors' Ability to Deliver	2013	South Africa, Southern Africa	Lay Counselors (LCs)	Usually women carrying out functions related to health care	39	HIV	**Type:** Refresher training course and supportive	**Outcome measure(s):** Knowledge and skills assessment.	No details

	Counseling for Behavior Change.				delivery but who have no formal professional or paraprofessional certificate or degreed tertiary education. Their role specific to HIV relates to enhancing treatment adherence and encouraging safer sexual practice.			supervision. **Content:** Refresher training was aimed at covering difficulties counselors were experiencing with the eight-step ‘Options’ protocol and its delivery in the clinic setting. Supportive supervision recapped the protocol and MI principles. Learning was facilitated by means of demonstration, group discussion, role-plays, and self-evaluation. **Duration:**14 hours of refresher training over a two-day period and four 1-hour supportive supervision courses over a period of 4-months. **Provider:** Two counseling psychologists with experience in training lay	Lay counselors ability in motivational interviewing was assessed following refresher training using the Motivational Interviewing Treatment Integrity Tool and an instrument developed by the researchers.<br><br>**Outcome(s):** Although LCs did not achieve complete proficiency in MI, refresher training and supervision improved LCs basic counseling communication skills and therapeutic approach	

								counselors from the Western Cape Provincial Department of Health provided the refresher training course and individual NGOs provided the supervision. **Location:** No details		
Gallo et al.[41]	Evaluation of a volunteer community-based health worker programme for providing contraceptive services in Madagascar.	2013	Madagascar, East Africa	Community Health Workers (CHWs)	CHWs deliver maternal, reproductive health and family planning services.<br><br>Receive an initial 10-day training. Unpaid but can receive a small profit from commercial goods they sell e.g. condoms, oral and injectable contraception	100	Maternal and Reproductive Health	**Type:** Refresher training course. **Content:** No details **Duration:** Two-day refresher training course for those who did not meet the minimum level of competency following initial training. **Provider:** No details **Location:** No details	**Outcome measure(s):** Knowledge and skills assessment. A test involving five stimulated encounters regarding knowledge of an injectable contraception<br><br>**Outcome(s):** Refresher training resulted in higher scores on the clinical test, which consisted of assessing knowledge of injectable contraception, and five observed simulated client encounters.	No details
Gupta et al.[42]	Implementation of ORT:	1994	India, South Asia	Community Health	CHGs in this study were	323	Child Health	**Type:** Refresher training	**Outcome measure(s):**	No details

	some problems encountered in training of health workers during an operational research programme.			Guides (CHGs)/Anganwadi Health Workers (AHWs)	grassroots level, part time volunteers selected by village leaders from amongst the local residents. They receive 3 months of pre-service training and serve approximately 1000 people. Their role is to deliver primary healthcare in the village.<br><br>AHWs are also part time workers, with one AHW per village. They were trained for 3 months and their primary role was to deliver nutrition and healthcare services to children.<br><br>The government has overall responsibility for the workers.			course.<br>**Content:** Refresher course in Bengali recapping knowledge and containing important practical skills such as how to prepare ORS solution<br>**Duration:** One-day interactive refresher training course.<br>**Provider:** No details<br>**Location:** No details	Knowledge and skills assessment. Testing for improved knowledge of ORS using role playing and discussions.<br><br>**Outcome(s):** Following refresher training knowledge and skills on features such as use of home fluids, preparation of ORS and dosage of ORS was increased.	
Gupta et al.[43]	Improving quality of home-based postnatal	2016	India, South Asia	Auxiliary Nurse Midwives (ANMs)	ANMs receive 8 days of training in 'Integrated Management of	12	Child Health	**Type:** In-service training.<br>**Content:** microteaching to	**Outcome measure(s):** Knowledge and skills assessment.	No details

	care by microteaching of multipurpose workers in rural and urban slum areas of Chandigarh, India: a pilot study.				Newborn and Childhood Illnesses' and undertake 'skill based work'<br><br>No further details on remuneration or level of education.			enhance the postnatal care skills of ANMs<br>**Duration:** One 90-minute session every three months.<br>**Provider:** A Lady Health Visitor (LHV) and a male social worker<br>**Location:** A health post of the Dept. of Community Medicine, School of Public Health, Chandigarh.	Scores achieved on a structured checklist with items regarding maternal history and exam taking technique, new-born examination and maternal counselling.<br><br>**Outcome(s):** Maternal examination, maternal counseling regarding danger signs and newborn examination all improved significantly after the third round of microteaching. In addition more ANMs carried weighing scales, thermometers, and registers after receiving training.	
Hadi.[44]	Management of acute repiratory infections by community health volunteers: experience of Bangladesh	2003	Bangladesh, South Asia	Community Health Volunteers (CHVs)	The role of CHVs was to detect and treat cases of acute respiratory infection & to refer severe and complicated cases to nearby health clinics.	120	Child Health	**Type:** Supervision<br>**Content:** No details<br>**Duration:** Once a month.<br>**Provider:** Paramedics from BRAC<br>**Location:** No	**Outcome measure(s):** Knowledge and skills assessment. Comparing the diagnosis of acute respiratory infection and management between CHWs and	No details

	Rural Advancement Committee (BRAC).				Unpaid, selected from among the local area: most had only 5 years of schooling.<br><br>3 days of basic training covering theoretical and practical concepts of acute respiratory tract infections.			details	trained assessors.<br><br>**Outcome(s):** The "sensitivity, specificity, and overall agreement rates in diagnosing and treating ARIs" were significantly higher among the CHVs who were supervised.	
Horwood et al.[45]	A continuous quality improvement intervention to improve the effectiveness of community health workers providing care to mothers and children: a cluster randomised controlled trial in South Africa	2017	South Africa, Southern Africa	Community Health Workers (CHWs)	CHWs are recruited and deployed by the Department of Health and receive a small stipend.<br><br>They fulfill a variety of roles in the community including home-based care, education on prevention of mother to child transmission of HIV, adherence support for antiretroviral and TB treatment, and provision of maternal and child health services using	120	Maternal and Child Health.	**Type:** Supervision **Content:** Sessions focused on areas for improvement which were jointly decided with CHWs and supervisors **Duration:** Twice monthly. **Provider:** Mentors based at the University of KwaZulu-Natal **Location:** No details	**Outcome measure(s):** Mixed methods. Knowledge and skills were assessed using four questions, which were asked to mothers served by the CHWs regarding antenatal care. Markers including the number of household visits performed by the CHW assessed behaviour change.<br><br>**Outcome(s):** CHW visits during pregnancy and the postnatal period were significantly higher in the CHW	No details

					iCCM.				group who received supervision.<br>Mothers seen by CHWs who had received supervision demonstrated higher maternal and child health knowledge scores and reported higher exclusive breastfeeding rates. Similarly, HIV-positive mothers were more likely to have disclosed their HIV status to the CHW<br>however, uptake of facility-based interventions were not significantly different.	
Javanparast et al.[46]	The experience of community health workers training in Iran: a qualitative study.	2012	Iran, Middle East	Behvarz	Behvarz are full time employees of the health system. They are selected from her/his own community and work in the 'Village Health House' - the most peripheral health delivery facility in the rural areas of Iran.	91	General focus	**Type:** In-service training<br>**Content:** Updates on new policies and programmes, reinforcement of initial training concepts, and ensuring they are practicing skills learned correctly<br>**Duration:** Variable - ranging	**Outcome measure(s):** Qualitative evaluation e.g. interviews with behvarz.<br><br>**Outcome(s):** Compared to pre-service training, in-service training was viewed unfavourably by the behvarz.<br>They complained	No details

					They have a two year period of training.			from monthly to bi-annually.<br>**Provider:** GPs or other allied health workers<br>**Location:** Rural Health Centres	about "its quality and timing, the infrequency of courses, inadequately qualified trainers who are unfamiliar with the behvarz working environment, the lack of practical sessions and of physical space and training facilities".	
Joos et al.[47]	Evaluation of a mHealth data Quality Intervention to Improve Documentation of Pregnancy Outcomes by Health Surveillance Assistants in Malawi: A cluster RCT.	2016	Malawi, East Africa	Health Surveillance Assistants (HSAs)	HSAs are government trained and paid CHWs.<br><br>They are attached to a local health center and serve approximately 1000 people.<br><br>The scope of their work varies but specific to this project it involved training on the documentation of pregnancies, births, and deaths.	160	Maternal Health	**Type:** Supervision<br>**Content:** The intervention group received SMS messages containing motivational and data quality content.<br>**Duration:** 2-5 SMS messages were sent each week.<br>**Provider:** Mobile based<br>**Location:** NA	**Outcome measure(s):** Change in behaviour, attitudes or practice e.g. Improved recording of pregnancy.<br><br>**Outcome(s):** Improved documentation of pregnancies was observed in both the intervention and control groups.	**Yes** - one-way SMS messages that were sent to HSAs on a regular basis during a 12-month period and reporting on pregnancy outcomes was assessed. Two arms to the study. HSAs in the treatment group received high volume motivational and data quality SMS. HSAs in the control group only received low volume

										motivational SMS.<br><br>Labrique classification:<br>-Provider training and education
Kawasaki et al.[48]	Reactions of community members regarding community health workers' activities as a measure of the impact of a training programme in Amazonas, Brazil.	2015	Brazil, South America	Community Health Workers (CHWs)	Nationwide CHW programme coordinated by the MoH known as Programa dos Agentes Comunitários de Saúde (PACS).<br><br>CHWs are paid.<br><br>Multiple responsibilities including home visits, health promotion, vaccination, record keeping, community meetings.	102	General focus	**Type:** Refresher training course.<br>**Content:** CHWs were trained on facilitating adequate use of health-care services, and health promotion guidance based on the CHW manual published by the MoH plus mention of ad-hoc training<br>**Duration:** Once a month refresher training sessions.<br>**Provider:** An NGO in partnership with the city and state hospital<br>**Location:** In the hospital in the city	**Outcome measure(s):** Change in behaviour, attitudes or practice. Baseline and endline surveys concerning recognition and satisfaction with respect to CHW performance among members of the community were conducted<br><br>**Outcome(s):** Increased awareness of the work of CHWs amongst the community after refresher training courses and better partnership work between supervisors and CHWs was observed.<br><br>The survey also	No details

									revealed an increase in home visits, greater levels of recognition of CHW functions, and increase levels of satisfaction from community members.	
Kuule et al.[49]	Community Health Volunteers in Primary Healthcare in Rural Uganda: Factors Influencing Performance.	2017	Uganda, East Africa	Community Health Volunteers (CHVs)	CHVs are part of the Village Health Team programme in Uganda.<br><br>The CHVs are trained and maintained by a variety of organisations, including NGOs.<br><br>They are largely unpaid.<br><br>They are expected to engage in a variety of activities including general tasks in all primary health-care core areas e.g. home visits, community information management, health promotion and education, management of	508	Child & Maternal and Reproductive Health	**Type:** Refresher training course and supervision.<br>**Content:** updates on issues such as symptoms of childhood illnesses; key indicators for referrals and how to monitor children for malnutrition.<br>**Duration:** Biannual refresher training sessions and monthly supervisions.<br>**Provider:** Community health nurses<br>**Location:** Hospital setting	**Outcome measure(s):** Change in practice, attitudes or behaviour e.g. attendance at meetings, household follow-up and reporting, immunization coverage.<br><br>**Outcome(s):** Refresher trainings were associated with improved performance, however due to multiple confounding variables they could not be determined to be causative.	No details

					common illnesses, and follow-up of pregnant women.					
Mash et al.[50]	Reflections on the training of counsellors in motivational interviewing for programmes for the prevention of mother to child transmission of HIV in sub-Saharan Africa.	2008	South Africa, Southern Africa	Lay counsellors (LCs)	No details provided	18	HIV	**Type:** Supervision **Content:** Recapping of motivational interviewing techniques **Duration:** Once monthly **Provider:** Four trained action researchers and counsellors **Location:** No details	**Outcome measure(s):** Mixed methods knowledge and skills assessment through assessment of motivational interview techniques and qualitative feedback from supervisors.<br><br>**Outcome(s):** The lay counsellors were not proficient in motivational interviewing despite receiving on going training.<br><br>Qualitative feedback revealed that a lot of time during the on-going training was spent on covering "really basic information".	No details
McLean et al.[51]	Task sharing in rural Haiti: Qualitative assessment of a brief, structured training with	2015	Haiti, Central America	Ajan Santé (community health workers) and Promoteurs (community members	CHWs in this study were largely providing services for HIV/AIDS and cholera prevention prior to the study which	3	Mental Health	**Type:** Supervision **Content:** Recapping of knowledge and skills regarding mental health diagnosis and	**Outcome measure(s):** Mixed methods. Change in practice and behaviour measured through home visits, provision of	No details

	and without apprenticeship supervision for community health workers.			who provide health education through song).	focused on mental health service provision.<br><br>There was a lack of detail regarding financial remuneration and pre-service training.			symptoms, observed practice and details of how to carry out home visits<br>**Duration:** After initial training one week of daily observation by a licensed counselor followed by one further week of supervised sessions.<br>**Provider:** a licensed counselor<br>**Location:** No details	supportive visits and referrals. Qualitative assessment of confidence and satisfaction.<br><br>**Outcome(s):** With supervision there was a greater number of home visits. Qualitative findings support the added value of supervision according to trainees.	
Mengistu et al.[52]	Effect of performance review and clinical mentoring meetings (PRCMM) on recording of community case management by health extension workers in Ethiopia.	2014	Ethiopia, East Africa	Health Extension Workers (HEWs)	HEWs received training using iCCM materials on assessment and treatment of childhood pneumonia, malaria, diarrhea and malnutrition.<br><br>iCCM training was supported by the government and Save the Children (NGO).<br><br>No details on remuneration.<br><br>No details on pre-service education	1175	Child health	**Type:** Supervision<br>**Content:** A review of HEWs records took place on day one and clinical mentoring took place on day two.<br>**Duration:** Twice monthly for two days.<br>**Provider:** Health workers trained as trainers<br>**Location:** A meeting hall in a central town	**Outcome measure(s):** Change in practice, attitudes or behaviour e.g. Improved recording and adherence to iCCM guidelines.<br><br>**Outcome(s):** Supervision improved iCCM performance of HEWs and the authors recommended that as such it should be integrated within the PHC system and given about every six	No details

					levels.				months.	
Miller et al.[53]	Assessment of the impact of quality improvement interventions on the quality of sick child-care provided by Health Extension Workers in Ethiopia.	2016	Ethiopia, East Africa	Health Extension Workers (HEWs)	All HEWs are literate women with at least a tenth–grade education, who receive a one–year pre–service training. The pre–service training covers iCCM.<br><br>Following the training, they are recruited as government employees and deployed to work out of health posts at the kebele (sub–district) level.<br><br>There are typically two HEWs working at one health post, which serves approximately 5000 people.	157 (based on an estimate of 1.5 HEWs across 104 health posts)	Child health	**Type:** Refresher training course and supervision **Content:** Refresher training and supportive supervision focussed on reinforcing knowledge and skills learned during the initial iCCM training, carrying out observed visits and checking record keeping before identifying gaps for improvement. **Duration:** Refresher training was a half-day to one-day course eight weeks after intial iCCM training. Supportive supervision was carried out quarterly. **Provider:** Refresher training was provided by iCCM trainer from the district or from	**Outcome measure(s):** Change in behaviour**,** attitudes and practice e.g. number of children correctly managed according to iCCM guidelines.<br><br>**Outcome(s):** Children managed by a HEW who had attended a refresher training course were eight times more likely to be correctly managed, compared to children managed by a HEW who did not. Management by an HEW who received refresher training also significantly increased the odds of correct management**,** whereas the supportive supervision element did not significantly affect the odds of receiving correct care.	No details

								an implementing partner agency. Supervision was provided by implementing partner NGO staff, and sometimes health center staff or woreda health officials.<br>**Location:** Both were held at a local health post		
Mkumbo et al.[54]	Innovation in supervision and support of community health workers for better newborn survival in southern Tanzania.	2014	Tanzania, East Africa	Community Health Volunteers (CHVs).	CHVs were trained to carry out home visits in the first week of life to promote warmth, hygiene and breastfeeding, in order to try improve newborn survival.<br><br>Work in a voluntary capacity.	824	Child health	**Type:** Supervision<br>**Content:** Review of work around newborn checklist, discussion of individual needs<br>**Duration:** Quarterly meetings.<br>**Provider:** Nurse<br>**Location:** Community based visits	**Outcome measure(s):** Change in behaviour, attitudes and practice e.g. number of volunteer–supervisor contacts.<br><br>**Outcome(s):** The community-linked supervision approach resulted in over 50 times more supervision contacts than during the facility-only supervision approach**.**	No details
Msisuka et al.[55]	An evaluation of a refresher training intervention for HIV lay counsellors	2011	Zambia, East Africa	Lay counsellors (LCs)	Lay counsellors are community members recruited by the Zambian Ministry of Health who become certified	25	HIV	**Type:** Refresher training course<br>**Content:** Testing for HIV, counselling and safety. Training	**Outcome measure(s):** Mixed methods. Knowledge and skills assessment was answering true or false questions	No details

	in Chongwe District, Zambia.				after completing a 7-week national training package for psychosocial counseling for HIV. The training package includes a 2-week theoretical component followed by a 5-week supervised practical component. The training covered HIV infection, appropriate values and attitudes for counselors, behaviour change communication, psychosocial support, pre-test and post-test counselling, and professional ethics.			materials were adapted from the National Counseling and Testing Training Curriculum **Duration:** Two-day refresher training course. **Provider:** National trainers for psychosocial Counseling from the National AIDS Counsel **Location:** One central location in the Chongwe district	on a 25-question quiz and testing 10 blood panel samples. An attitudes assessment regarding motivations and obstacles to performance was carried out using a questionnaire.<br><br>**Outcome(s):** Refresher training increased knowledge domains in all areas, particularly in standard precaution and post-exposure prophylaxis.<br><br>52% of LCs responded that periodic opportunities to update their knowledge and skills are crucial to their continued work as LCs.	
Ndima et al.[15]	Supervision of community health workers in Mozambique: a	2015	Mozambique, East Africa	Agentes Polivalentes Elementaires (APEs)	APEs are volunteers, trained by the MoH, They commit to certain terms through a "	18	Child & Maternal and Reproductive Health	**Type:** Supervision **Content:** A checklist was used which covered several areas, including	**Outcome measure(s):** Mixed methods. Qualitative assessment e.g. interviews regarding	No details

	qualitative study of factors influencing motivation and programme implementation.				contract” which outlines their right to an allowance or subsidy and free health care at the local primary health centre or dispensary.<br><br>They receive a 4-month residential pre-service training in iCCM and maternal health.			whether APEs had particular commodities available, and if they were completing and recording their duties correctly<br>**Duration:** Monthly supervisions at the community health centre and quarterly supervisions in the community.<br>**Provider:** Qualified nurses attached to a health center<br>**Location:** Mix between health centre and community based	motivation and change in practice e.g. number of home visits and referrals.<br><br>**Outcome(s):** Supervision was irregular and infrequent, affecting APEs motivation. When it did occur, supervision was felt to focus more on fault-finding than being supportive in nature.<br>Supervisors, felt unsupported with high concurrent workloads in health facilities, where they had multiple roles.<br>A lack of resources for supervision activities was identified, and supervisors felt caught up in administrative issues around APE allowances that they were unable to solve. Many supervisors were not trained in	

									providing supportive supervision.	
*Puchalski Ritchie et al.[56]	A knowledge translation intervention to improve tuberculosis care and outcomes in Malawi: a pragmatic cluster randomized controlled trial.	2015	Malawi, East Africa	Health Surveillance Assistants (HSAs)	HSAs are a formal cadre of paid lay health worker. Their roles include provision of outpatient TB care and adherence support. At the time of this study, pre-service training for general HSAs consisted of 10 weeks of in-class training, with approximately 1 day devoted to TB control, transmission, and treatment.<br><br>A subgroup of HSAs, termed TB focus LHWs, receive 2 weeks of additional TB specific training and are responsible for the provision of TB care at the health center	49	TB/HIV	**Type:** In-service training<br>**Content:** Case-based discussions and role playing covering TB transmission, treatment, and consequences of poor adherence; the interaction of TB and HIV; common barriers to adherence and appropriate methods for preventing and addressing non-adherence. There was also training on the use of a clinical support tool.<br>**Duration:** Six on-going training courses lasting for 60-90 minutes over three-months.<br>**Provider:** TB focus LHWs<br>**Location:** Local health centres	**Outcome measure(s):** Change in behaviour, attitudes or practice. Measured through assessing adherence to TB medications and improvements in clinical conditions at the community level.<br><br>**Outcome(s):** There was no different between the control and intervention groups regarding the proportion of treatment successes.	No details

					level. TB focus LHWs recruit and train general LHWs to assist with TB care.					
*Puchalski Ritchie et al.[57]	Lay Health Workers experience of a tailored knowledge translation intervention to improve job skills and knowledge: a qualitative study in Zomba district Malawi.	2016	Malawi, East Africa	Health Surveillance Assistants (HSAs)	See description provided above.	36	TB/HIV	**Type:** In-service training **Content:** Case based role playing and discussions covering topics such as TB transmission and natural history, the interaction of TB and HIV, TB treatment including side-effects and their management, common barriers to adherence, consequences of poor treatment adherence, and approaches to preventing and addressing poor adherence **Duration:** Six on-going training courses lasting for 60-90 minutes over three-months. **Provider:** TB focus LHWs **Location:** Local	**Outcome measure(s):** Qualitative assessment. Interviews with CHWs regarding perceived improvement in knowledge and skills and ability to perform their roles.<br><br>**Outcome(s):** Generally the in-service training was well received. HSAs reported increased TB, HIV, and job-specific knowledge; improved clinical skills; and increased confidence and satisfaction with their work. Suggestions for improvement were less consistent across participants, but included: increasing the duration of the	No details

								health centres	training, changing to an off-site venue, providing stipends or refreshments as incentives, and adding HIV and drug dosing content	
**Rabbani et al.[58]	Health workers' perspectives, knowledge and skills regarding community care management of childhood diahorrea and pneumonia: a qualitative inquiry for an implementation research project "Nigraan" in District Badin, Sindh, Pakistan.	2016	Pakistan, South Asia	Lady Health Workers (LHWs)	LHWs provide preventive and basic curative maternal, newborn and under five child health (MNCH) services in their catchment area. LHWs are salaried staff, recognized by the government and are preferably married and educated (minimum eight years of schooling). They mostly reside in the area where they serve. An LHW serves approximately 100– 150 households, representing an average population of 1000.	108	Child Health	**Type:** Supervision **Content:** Supervisory visits involve the LHW being accompanied during their home visits, where their supervisor guides them and addresses their concerns **Duration:** Twice monthly. **Provider:** Lady Health Supervisors **Location:** Community based	**Outcome measure(s):** Mixed methods. Knowledge and skills assessment regarding management plus qualitative perceptions of the supervision.<br><br>**Outcome(s):** LHWs considered adequate supervision and the presence of LHSs during household visits as a factor facilitating their performance. LHWs did not have a preference for written or verbal feedback, but supervisors considered written individual feedback to LHWs to be more useful than group and verbal feedback.	No details

					The LHW also works from her home, where she is encouraged to have a portion of her home designated as a " Health House".<br><br>The are supervised by Lady Health Supervisors (LHSs). LHSs are attached to the first level care facility (FLCF) and are responsible for on-going supervision and monitoring of LHWs.<br>LHSs are female health workers aged 22– 45, residing locally with a good educational background and have several years' experience as a LHW.<br>Salaries range from $160-180/month. Each LHS supervises approximately 15– 25 LHWs.					

**Rabbani et al.[59]	Inspiring health worker motivation with supportive supervision: a survey of lady health supervisor motivating factors in rural Pakistan.	2016	Pakistan, South Asia	Lady Health Supervisors (LHSs)	See description of LHSs provided above.	29	Child Health	**Type:** Supervision<br>**Content:** Supervisory visits involve the LHW being accompanied during their home visits, where their supervisor guides them and addresses their concerns<br>**Duration:** Twice monthly<br>**Provider:** LHSs<br>**Location:** Community based	**Outcome measure(s):** Qualitative measures e.g. motivation following supervision.<br><br>**Outcome(s):** Lady health supervisors are motivated by both their role in providing supportive supervision to lady health workers and by the supervisory support received from their coordinators and managers.	Mobile phones were provided to improve communication and coordination between LHSs and LHWs regarding case detection, tracking, management, and follow-up.<br>Labrique classification:<br>-Provider to provider communication
Roberton et al.[60]	Initial experiences and innovations in supervising community health workers for maternal, newborn, and childhealth in Morogoro region,	2015	Tanzania, East Africa	Community Health Workers (CHWs)	CHWs are volunteers. Their role includes identifying pregnancies, conducting routine home visits to antenatal and postpartum women and facilitating group-based discussion sessions in the community.	228	Maternal and Child Health	**Type:** Supervision<br>**Content:** Different supervisors have different roles. Facility based supervisors responsibilities include providing technical support to CHWs to facilitate community mapping and household census,	**Outcome measure(s):** Mixed methods. Qualitative interviews assessing CHWs experiences and change in behaviour, attitudes or practice measured through survey data recording frequency and content of CHW supervision.	No details

	Tanzania.				The topics of these discussions include antenatal care, danger signs, birth preparedness, maternal and child nutrition, postpartum and newborn care, family planning, and HIV/AIDS.<br><br>CHWs are required to be residents of the village, over age 18, role models for maternal and child health in their community, and preferably with at least form four level of schooling.<br><br>CHWs self nominate themselves prior to village governments nominating their top candidates. Selection of of CHWs was finalized at			MoH supervisors used supervisions to provide CHWs with working tools and stipends whereas village leaders work at a local level for advocacy, support and community awareness<br>**Duration:** Once monthly from facility based supervisors and quarterly from MoH teams and village leaders<br>**Provider:** A mixed provider model including facility health workers trained in supportive supervision through a 2-week " Community MNCH Supervisor' s Training" programme, village leaders and<br>**Location:** Mixture between primary health clinic and in the field	**Outcome(s):**<br>CHWs value supervision and appreciate the sense of legitimacy that arises when supervisors visit them in their village. Village leaders and district staff are engaged and committed to supporting CHWs. Despite these successes, facility-based supervisors visit CHWs in their village an average of only once every 2.8 months, CHWs and supervisors still see supervision primarily as an opportunity to check reports, and meetings with district staff are infrequent and not well scheduled.	

					village meetings.					
Rowe et al.[61]	Longitudinal analysis of community health workers' adherence to treatment guidelines, Siaya, Kenya, 1997-2002.	2007	Kenya, East Africa	Community Health Workers (CHWs)	CHWs were trained by CARE, an NGO.<br><br>Their role was to assess, diagnose and treat children under age 5 years according to the CARE Management of the Sick Child (MSC) guidelines, a simplified version of the WHO/ UNICEF iCCM guidelines.	114	Child Health	**Type:** Refresher training course<br>**Content:** Knowledge reviews followed by working on weaknesses in CHW clinical skills that were identified by performance assessments and practical sessions in small groups<br>**Duration:** Two three-month blocks of refresher training.<br>**Provider:** No details<br>**Location:** No details	**Outcome measure(s):** Change in behaviour, attitudes or practice e.g. correct referrals and management of sick children.<br><br>**Outcome(s):** The study revealed that immediately after the first refresher training, the mean adherence level of CHWs to the guidelines for managing sick children improved for patients with a severe illness, but worsened for patients without severe illness. Adherence scores declined rapidly during the 6 months after the second refresher training. The authors concluded that the first refresher was partially effective but the second refresher had an effect contrary to that intended, and patient	No details

									characteristics had a strong influence on adherence patterns.	
Singh et al.[13]	Supportive supervision for volunteers to deliver reproductive health education: a cluster randomized trial.	2016	Uganda, East Africa	Community Health Volunteers (CHVs) and Community Health Workers (CHWs)	CHVs role in this context was to offer preventative reproductive health care. They had "relatively short training" and they volunteer 5–10 h per week, receiving little or no remuneration and poor supervision after an initial training period.<br><br>Groups of CHVs are known as Village Health Teams and are often maintained by various NGOs<br><br>The CHWs were younger demographic with higher basic education. Trained for 6 months to 2 years as full-time members of the health system.	82	General focus	**Type:** Supervision **Content:** The supervision sessions involved accompaniment on a home visit and topics covered were those from previous training sessions such as encouraging birth at a health facility or danger signs in pregnancy **Duration:** Monthly training lasting for between two to three hours per month. **Provider:** CHWs **Location:** In the field (home visits)	**Outcome measure(s):** Change in behaviour, attitudes or practice e.g. immunizations, breastfeeding, number of installed tippy taps for hand washing assessed at the household level.<br><br>**Outcome(s):** Overall this study demonstrated an increase in desired behaviors in both the intervention and control arms over the study period. Both arms showed high retention rates of CHVs. At 1 year follow-up there was a significantly higher prevalence of installed and functioning tippy taps for hand washing in the intervention villages than control villages. All outcome and	No details

									process measures related to home-visits to homes with pregnant women and newborn babies favored the intervention villages.	
Sylla et al.[62]	Low level educated community health workers training: a strategy to improve children access to acute respiratory treatment in Senegal	2007	Senegal, West Africa	Les agents de santé communautaire (ASC)	ASCs are volunteers serving their communities. They offer their services within health huts. They are recruited by the government and are typically have at least a primary level of education and are able to read and write in French.<br><br>In this programme ASCs received a 3-day preservice training course using the WHO guidelines for Acute Respiratory Illness.	107	Child Health	**Type:** In-service training<br>**Content:** Recapping material from the WHO guidelines to assess & manage acute respiratory illness (ARI)<br>**Duration:** Once a month<br>**Provider:** Head nurse<br>**Location:** Health centres	**Outcome measure(s):** Change in behaviour, attitudes or practice.<br><br>**Outcome(s):** ASCs who were trained and supported with follow-up could help provide care to children with ARI in the community by following the WHO guidelines for ARI recognition and management, however given that 28% of severe pneumonia cases were misclassified as pneumonia it would be important to emphasize the recognition of danger signs and the follow-up of severe cases.	No details
Talukder	In a rural	2016	Banglades	Traditional	TBAs are also	N/A –	Child	**Type:** Supervision	**Outcome**	No details

et al.[63]	area of Bangladesh, traditional birth attendant training improved early infant feeding practices: a pragmatic cluster randomized trial.		h, South Asia	birth attendants (TBAs) / Community Volunteers (CVs)	known as 'skilled birth attendants' and are government trained. There are approximately 7500 working across Bangladesh. Their roles are include assisting deliveries and advising mothers on breastfeeding.<br><br>CVs are community based volunteers such as relatives, friends or neighbours. No information was provided on their exact roles or training.	randomisation done at district level	health	**Content:** Field supervisors checked on breastfeeding activities in the community<br>**Duration:** Once a week supervision sessions.<br>**Provider:** Field supervisors<br>**Location:** In the field	**measure(s):** Change in behaviour, attitudes or practice e.g. number of home visits, initiation of breastfeeding<br><br>**Outcome(s):** Although outcome measures, such as rate of breast feeding and avoidance of pre-lacteal feeds, improved in both groups, there was no significant difference between outcome measures in the group that had received just training compared to training plus supervision	
Vu Henry et al.[14]	Enhancing the Supervision of Community Health Workers With WhatsApp Mobile Messaging: Qualitative Findings From 2	2016	Kenya, East Africa	Community Health Volunteers / Community Health Extension Workers (CHEWs)	CHVs are volunteer Community Health workers. No further details were provided on specific cadre roles.<br><br>CHEWs are the CHV supervisors.	25	General focus	**Type:** Supervision<br>**Content:** Messages were sent between CHVs and CHEWs regarding assessing childhood development Milestones.<br>**Duration:** Continuous supportive supervision over a	**Outcome measure(s):** Qualitative analysis of WhatsApp messages.<br><br>**Outcome(s):** The thematic analysis revealed that most of the content related to creating a social environment, sharing	Yes – WhatsApp groups were created between supervisors and CHWs to support supervision, professional development, and team building.

	Low-Resource Settings in Kenya.							period of six months via WhatsApp. **Provider:** Through a communication group between CHWs and their supervisors installed on a mobile phone **Location:** Not applicable (mobile based)	communication and information, or promoting quality of services.	Labrique classification categories:<br><br>-Provider to provider communicatio n<br>-Provider training and education<br>-Human Resource Management
Vallières et al.[64]	There's No App for That: Assessing the Impact of mHealth on the Supervision, Motivation, Engagement, and Satisfaction of Community Health Workers in Sierra Leone.	2016	Sierra Leone, West Africa	Community Health Workers (CHWs)	CHWs in this study were trained by World Vision Ireland' s Access to Infant and Maternal Health programme.<br><br>Recruitment was done in accordance with the Policy for Community Health Workers in Sierra Leone published by the MoH.<br><br>CHWs in this model are volunteers, undergo a minimum 10-day basic training	292	Maternal and child health	**Type:** Supervision **Content:** The MOTECH suite app allowed CHWs to register pregnant women and their children, alert CHWs when household visits are overdue, allow CHWs to make referrals to their affi liated PHU, and collect household data during household visits. **Duration:** 6-months **Provider:** MOTECH suite **Location:** Mobile based application	**Outcome measure(s):** Change in behaviour, attitudes or practice measured through self-reported measures of work engagement and job satisfaction.<br><br>**Outcome(s):** There was no differences between the perceived supervision and motivation across the different groups of CHWs over time with the introduction of the MOTECH Suite as a human resource management tool. Furthermore, there	**Yes** - assessed the use of the Mobile Technology for Community Health (MOTECH) Suite application on the perceived organizational factors of a CHW programme. The MOTECH suite allows CHWs to "register pregnant women and their children …alert CHWs when

					course and be a resident of the village.<br><br>They serve between 100-500 people.				was no significant change in the self-reported measures of work engagement and job satisfaction across each of the intervention groups over time.	household visits are overdue, allow CHWs to make referrals to their local health unit and collect household data".<br><br>Labrique categories:<br>-Registries or vital event tracking<br>-Data collection and reporting<br>-Provider workplanning and scheduling<br>-Provider to provider communicatio n<br>-Human Resource Management
Zeitz et al.[65]	Community health worker competency in managing acute respiratory	1993	Bolivia, South America	Communtiy Health Workers (CHWs)	CHWs in this study were recruited and managed by three different NGOs.<br><br>They were all	80	Child Health	**Type:** Refresher training course<br>**Content:** Knowledge and skills pertaining to the recognition of acute respiratory	**Outcome measure(s):** Knowledge and skills assessment using a pre- and post-intervention test.	No details

	infections of childhood in Bolivia.				volunteers with additional jobs with many working in agriculture. Four-fifths of the members of all three groups were literate and able to count.<br><br>They had all received variable training based on the WHO acute respiratory infections guidelines (*some of which were out-dated*).			infection in children using the results of a pre-course evaluation.<br>**Duration:** One-day refresher course lasting for eight hours.<br>**Provider:** Physicians, nurses, and auxiliary health workers who were routinely involved with training CHWs in ARI management.<br>**Location:** No details	**Outcome(s):** Improvements were seen across the pre and post test assessments following refresher training and statistically significant improvements were observed in key domains including identification of danger signs, acute respiratory tract infection classification and treatment.	

Legend. A table outlining key details from the studies included within the final scoping review.

Key

*Two separate analyses from the same study. One study focused on the number of HIV and TB cases correctly managed following a knowledge translation intervention, the other focused on a qualitative evaluation of the same intervention.

**Two separate analyses from the same study. One study focused on a qualitative evaluation of Lady Health Supervisors feelings of motivation following provision of supervisor, the other focussed on a mixed methods assessment of Lady Health Workers regarding management of childhood diarrhoea and pneumonia following supervision plus qualitative perceptions of the supervision.
