# Supplementary material for: Ongoing training of community health workers in low-income and middle-income countries: a systematic scoping review of the literature
Source: BMJ Open. 2018 Apr 28;8(4):e021467. doi: 10.1136/bmjopen-2017-021467 (PMC5931295; doi:10.1136/bmjopen-2017-021467)
Supplement: Author's manuscript [file bmjopen-2017-021467.draft_revisions.pdf]

BMJ Open

BMJ Open is committed to open peer review. As part of this commitment we make the peer review history of every article we publish publicly available.

When an article is published we post the peer reviewers' comments and the authors' responses online. We also post the versions of the paper that were used during peer review. These are the versions that the peer review comments apply to.

The versions of the paper that follow are the versions that were submitted during the peer review process. They are not the versions of record or the final published versions. They should not be cited or distributed as the published version of this manuscript.

BMJ Open is an open access journal and the full, final, typeset and author-corrected version of record of the manuscript is available on our site with no access controls, subscription charges or pay-per-view fees (http://bmjopen.bmj.com).

If you have any questions on BMJ Open's open peer review process please email info.bmjopen@bmj.com

BMJ Open

The on-going training of Community Health Workers in low- and middle-income countries: A systematic scoping review of the literature.

Journal:	*BMJ Open*
Manuscript ID	bmjopen-2017-021467
Article Type:	Research
Date Submitted by the Author:	02-Jan-2018

Complete List of Authors:	O'Donovan, James; University of Oxford , Department of Education<br>O'Donovan, Charles; University of Leeds Faculty of Medicine and Health<br>Kuhn, Isla; University of Cambridge, Medical Library, School of Clinical Medicine<br>Sachs, Sonia; Earth Institute at Columbia University<br>Winters, Niall; University of Oxford, Education

Keywords:	MEDICAL EDUCATION & TRAINING, EDUCATION & TRAINING (see Medical Education & Training), PUBLIC HEALTH, Human resource management < HEALTH SERVICES ADMINISTRATION & MANAGEMENT, Health policy < HEALTH SERVICES ADMINISTRATION & MANAGEMENT, International health services < HEALTH SERVICES ADMINISTRATION & MANAGEMENT

SCHOLARONE™
Manuscripts

For peer review only

Full Title: The on-going training of Community Health Workers in low- and middle-income countries: A systematic scoping review of the literature.

Authors

James O'Donovan[1*], Charles O'Donovan[2], Isla Kuhn[3], Sonia Sachs[4], Niall Winters[1].

Affiliations

1. University of Oxford, Department of Education, Oxford, Oxfordshire, UK.
2. University of Leeds Faculty of Medicine and Health, Leeds, West Yorkshire, UK.
3. University of Cambridge, Medical Library, School of Clinical Medicine, Cambridge, UK.
4. Earth Institute at Columbia University, New York, NY USA.

*Corresponding Author

Address: University of Oxford, Department of Education, Norham Gardens, Oxford, OX2 6PY.

Telephone: +44 7999177071

Email: james.odonovan@seh.ox.ac.uk

Word Count (excluding title page, abstract, references, figures and tables)**:** 4879

For peer review only

Abstract

Objectives: Understanding the current landscape of on-going training for Community Health Workers (CHWs) in low- and middle-income countries (LMICs) is important both for organisations responsible for their training, as well as researchers and policy makers. This scoping review explores this under-researched area, informed by the following questions: How is on-going training for CHWs designed and delivered? Are theories of learning used to inform the design of on-going training? Do mobile technologies have a role in the delivery of on-going training? How are the outcomes of on-going training reported?

Design: Systematic scoping review.

Data sources: Medline, Embase, AMED, CINAHL, Web of Science, Scopus, ASSIA, LILACS, BEI and ERIC.

Study selection: Original studies focussing on the provision of on-going training for CHWs working in a country defined as low- or middle-income according to World Bank Group 2012 classification of economies.

Results: The scoping review found 36 original studies that met the inclusion criteria. On-going training activities for CHWs were described as in-service or refresher training (n=14), supervision (n=19) or a mixture of both (n=3). Most studies emphasised the importance of providing on-going training, however only eight sought CHW feedback to inform programme design in relation to training and only three referred to the use of learning theories. On-going training was mainly conducted in-person, however five studies used mobile technologies to support training delivery. The outcomes from on-going training activities were measured in different ways, including changes in practice and behaviour (n=13), knowledge and skills (n=7), qualitative assessments (n=8) or a mixed methods approach (n=8).

For peer review only

Conclusions: This review highlights the diverse range of on-going training for CHWs in LMICs. Given the expansion of CHW programmes globally, more attention should be given to the design, delivery, monitoring and sustainability of on-going training from a health systems strengthening perspective.

Keywords: Global Health, Community Health Worker (CHW), Lay health worker, Education, On-going training, Refresher training, In-service training, Supportive Supervision, Low- and middle-income country (LMIC)

Strengths and limitations of this study

- The study searched 10 major databases and the grey literature in order to include as many relevant studies as possible.
- This is one of the first known reviews to assess the provision of on-going training to CHWs in LMICs.
- Methodological quality assessment of the studies included did not take place, since this was a scoping review.
- The review is limited to papers published in the English language.
- There is no fixed definition of a Community Health Worker, and so some exclusions based on terminology may be debated.

Funding statement: This research received no specific grant from any funding agency in the public, commercial or not-for-profit sectors', however Dr. James O'Donovan is a DPhil candidate at The University of Oxford and is supported by a personal expenses and research support grant from The Economics and Social Research Council (ES/P000649/1).

Competing interests statement: "All authors have completed the ICMJE uniform disclosure form at *www.icmje.org/coi_disclosure.pdf* and declare: Dr. O'Donovan reports grants and personal fees from Economics and Social Research Council, during the conduct of the study;

For peer review only

no financial relationships with any organisations that might have an interest in the submitted work in the previous three years; no other relationships or activities that could appear to have influenced the submitted work."

For peer review only

Introduction

The World Health Organization (WHO) have forecast a global shortage of 18 million health workers by 2030.[1] One solution to address this gap has been to advocate for the recruitment, training and deployment of Community Health Workers (CHWs) in low- and middle-income countries (LMICs).[2] In the broadest sense, CHWs are lay people working within their own community in a health promotion and prevention role,[3] however their exact roles, responsibilities, recruitment, remuneration and training vary from country to country.[4] When provided with the correct resources, training, and support, CHWs have been proven to help improve health outcomes, improve accessibility to basic services and be highly cost effective in comparison to other cadres of salaried health workers, such as midwives and nurses.[2,5,6]

The WHO have suggested that for CHWs to fulfil their role successfully, they require "regular training and supervision".[7] For the purpose of this scoping review, we will focus specifically on evaluating the provision of on-going training for CHWs, rather than initial or pre-service training, since on-going training has typically been "the most neglected phase" of training,[8] with significant variability in terms of how it is delivered.[9] On-going training includes 'in-service' or 'refresher' training, defined as "follow on training received after a period of initial training",[10] or supportive supervision, defined as "a process of helping staff to improve their own work performance continuously… with a focus on using supervisory visits as an opportunity to improve knowledge and skills."[11]

Despite the importance placed on on-going training,[9] there is significant variation both in terms of its frequency, content, structure and monitoring between the different groups responsible for training CHWs.[9,12-14] For example, a study by Singh et al., found there were 22 different designated organisations responsible for training CHWs in Uganda.[12] The study also found that many of these organizations did not have specific training on "when, what and

For peer review only - http://bmjopen.bmj.com/site/about/guidelines.xhtml

how to supervise" CHWs.[12]

The frequency with which on-going training is provided appears to vary significantly between different organizations and countries. Guidelines produced by the USAID Health Care Improvement Project, recommend that refresher training should be provided at least every six months to update CHWs on new skills, reinforce initial training, and ensure they are practicing skills learned,[15] yet some CHWs have not had refresher training for over five years.[16] This finding of a poor provision of on-going training is commonplace and mentioned in several other studies, across multiple geographic contexts.[17-20] A multinational analysis from several countries in sub-Saharan Africa concluded that the current provision of refresher training courses was "not sufficient to meaningfully improve the quality of care in these countries," raising into question the need to assess the effectiveness of training programmes, both from the perspective of the individual CHW, but also the health system in which they operate.[21]

Although a systematic review was published in 2013 by Bluestone et al., evaluating effective in-service training design and delivery for health professionals more broadly,[22] there has been no review to specifically assess on-going training for CHWs in LMICs. A review published in 2014 by Hill et al., aimed to determine the impact of supportive supervision strategies for health workers in LMICs, however the scope of this review was relatively narrow, focusing just on supportive supervision, rather than on-going training more broadly and included multiple cadres of health workers.[23]

The questions we therefore aim to answer in this scoping review of the literature are:

1. How is on-going training for CHWs in LMICs designed and delivered?
2. How are on-going training programmes designed? Do they incorporate theories of learning? Are participatory approaches used to engage communities to ensure context-relevant design?

For peer review only

3. What role, if any, does mobile technology play in the delivery of on-going training?

4. How are the outcomes of on-going training reported and what measures are used?

For peer review only

Methods

Review approach

We conducted a systematic scoping review on the provision of on-going training for CHWs in LMICs. A scoping review is defined as “a form of knowledge synthesis that addresses an exploratory research question aimed at mapping key concepts, types of evidence, and gaps in research related to a defined area or field by systematically searching, selecting, and synthesizing existing knowledge”.[24] Scoping reviews are part of the family of research synthesis methods, but compared to systematic reviews address broader research questions. They aim to provide an overview and organisation of existing knowledge rather than a narrow synthesis of a predefined research question,[25,26] and place less emphasis on the critical appraisal of the included evidence compared to a traditional systematic review.[27]

A scoping literature review was chosen for this study since we wished to discover the gaps in the literature with regards to the provision of on-going training for CHWs in LMICs - an area that has not been reviewed before. This approach also enabled us to review a broad body of literature to better understand the current landscape of on-going training across a variety of contexts. This included mapping the extent, range and nature of how on-going training is provided and what future research needs to be undertaken.

Our scoping review followed explicit and transparent research steps to explore the research evidence on on-going training for CHWs in LMICs. A review protocol was not published, and the study was not registered with PROSPERO, as these mechanisms are not applied to scoping reviews.[24,25]

Search Strategy and Selection Criteria

The Cochrane Library, The Campbell Collaboration and The International Prospective Register of Systematic Reviews (PROSPERO) and grey literature were searched to identify

For peer review only - http://bmjopen.bmj.com/site/about/guidelines.xhtml

available or on-going systematic reviews pertaining to the provision of on-going training for CHWs in LMICs. No previous or on-going relevant reviews were identified.

We then designed an exhaustive and sensitive search strategy to identify all relevant studies. The search was developed with and reviewed by a medical librarian (IK) to ensure completeness. The search strategy was deliberately designed to be over inclusive. Relevant search terms for 'Community Health Workers' and 'on-going training' were developed (*see supplementary material*). These were combined to the World Bank Group 2012 list of LMICs[28] using the AND boolean operator to develop a master search string. Where appropriate, each index-linked MeSH term was exploded to contain all relevant subheadings. In addition, synonyms were searched for each key term, along with wildcards and truncation for free text words. A full record of the conducted search for each database is provided in the Supplementary Material. The following databases were searched to identify primary, peer-reviewed studies published from 12th September 1978, up to and including July 10th 2017:

- Medline;
- Embase and AMED via Ovid;
- CINAHL via Ebsco;
- Web of Science;
- Scopus;
- ASSIA via ProQuest;
- LILACS;
- British Education Index;
- ERIC

We wanted to ensure coverage of the relevant literature and education and the social sciences as well as medical sciences, hence including ERIC, BEI, ASSIA and Web of Science. We

For peer review only

For peer review only - http://bmjopen.bmj.com/site/about/guidelines.xhtml

also wanted to ensure broader coverage of global literature, hence the inclusion of LILACS, which give extensive coverage of Latin America and the Caribbean. The 12th of September 1978 was chosen as a cut-off date since this was the date of the Alma Ata Declaration, which identified CHWs as “one of the cornerstones of comprehensive primary health care”.[7]

Despite issues relating to data quality, we included non-peer reviewed ‘grey’ literature in this review in order to encapsulate a broad overview of the literature pertaining to refresher training for CHWs in LMICs. To identify relevant grey literature, we used the following sources; e-theses online service (ETHoS), conference proceedings on Index of Conference proceedings and Google Scholar. Finally, we also searched the reference lists of all relevant papers that we identified, using snowball sampling.

Inclusion and exclusion criteria

Studies were included if:

1. The primary participants were CHWs;
2. The CHWs worked in a country defined as low- or middle-income according to World Bank Group 2012 classification of economies;
3. It was explicitly stated that the objectives or aims of the study were to evaluate or assess the provision of on-going training, which could include refresher training, in-service training, continuing training or supportive supervision.

Studies were excluded if:

1. The primary focus of the paper was on health care professionals other than CHWs - for example, doctors, medical students, nurses, or allied healthcare professionals, such as midwives, were excluded.
2. The study was not conducted in a country defined as a LMIC according to World Bank Group 2012 classification of economies;

For peer review only

3. The paper was not an original, full text, research study. For example, commentaries, letters, opinion pieces, study protocols, training needs assessments and conference proceedings with only an abstract available, were all excluded.
4. The focus of the study was primarily on initial or pre-service training, rather than on-going training;
5. As part of the screening process during the full text review stage, studies were excluded if they did not report or describe the following three areas: (i) the design, (ii) the duration and frequency, and (iii) the outcomes, of the on-going training programme. It was deemed necessary that these three areas were commented upon in order that we had sufficient detail about the on-going training programme from which to base our analysis. These were also good screening questions from which to exclude studies for which the description and evaluation of on-going training was not the primary focus of the study, but rather was just mentioned briefly or in passing.
6. The full text of the study was not available in the English language.

Since the aim of our scoping review was to map the existing literature regarding the provision, design and outcomes of on-going training, both qualitative and quantitative study designs were included. Studies did not require a comparison group for inclusion.

Population: Although the nomenclature given to CHWs varies across the literature, for the purpose of this study we referred to the 2007 WHO definition:

"Community health workers should be members of the communities where they work, should be selected by the communities, should be answerable to the communities for their activities, should be supported by the health system but not necessarily a part of its organization, and have shorter training than professional workers."[29]

For peer review only

This definition allows for different types of health care workers to be classified as CHWs in different contexts. To clarify the ambiguity surrounding the term 'shorter training' given in the description above, we followed the definition from Lewin et al., to define shorter training as: "no formal professional or paraprofessional certificated or degreed tertiary education."[30]

Intervention: Studies had to focus on the provision of on-going training. For this review, on-going training is an umbrella term referring to any type of training a CHW can receive after a period of initial training. This can include refresher training, continuing training, in-service training or supportive supervision. We purposely aimed to encapsulate a broad range of on-going training subtypes, so as to better understand the current state of the field.

Research design: To be included, studies had to qualify as an original, full text, research study. This meant that review articles, commentaries, letters, policy briefs, protocols, training needs assessments and conference abstracts were not included. Generally, the original article had to include an introduction, explicitly state the aims of the study were to evaluate the provision of on-going training, and include a methods, results and discussion section to allow us to extract the necessary data for the questions we set out to answer.

Outcomes: No studies were excluded based on the measured outcomes, since one of the primary aims of this scoping review was to determine which measures are used to report the outcomes of on-going training programmes.

Study selection

All papers identified via database searching were exported into EndNote 7.1 and duplicate references removed. Titles and abstracts of all publications identified in the search were screened by two authors (JOD and COD). This determined whether they would be considered for a full text review. Those that were clearly irrelevant to the topic of this study were discarded at this stage. The full text of all the papers identified as potentially relevant by one

For peer review only

or both review authors was then retrieved and reviewed in full against the inclusion and exclusion criteria. At all stages, disagreements between the review authors were resolved via discussion or, if required, by seeking a third review from an independent researcher. Where appropriate, we contacted the authors of individual studies for further information. Once studies were determined to have met the inclusion criteria, the relevant data was systematically extracted from each study and tabulated using an Excel spreadsheet. Where necessary, the corresponding authors for relevant studies were contacted via email to clarify aspects of their work prior to final inclusion.

For peer review only

Results

Ten major databases and the grey literature were searched between June and July 2017. The initial searches yielded 3633 articles. After exclusion of duplicate references using the EndNote referencing system, 2319 papers were identified for initial screening. Studies were initially screened based on the title and their abstract by two separate reviewers (JOD & COD), resulting in 2153 studies being excluded – a result of our deliberately over-inclusive search strategy. 166 studies were then identified for an in-depth analysis of full text. Following this in-depth analysis 130 papers were excluded. Reasons for exclusion at full-text screening can be found in the PRISMA flowchart (Figure 1). As a result, we were left with 36 original studies that met the inclusion criteria.

17 different terms were identified as defining CHWs across the 36 studies, with 'Community Health Workers' being the most commonly used nomenclature (n=10) (*see Table 1, supplementary material*). The majority of studies (n=22) evaluating the provision of on-going training for CHWs have been published since 2015,[12-14,31-49] with no relevant studies published before 1993. In terms of geographic location, most studies took place in East Africa, (n=17)[12-14,34-36,38,41,43,46,50-56] or South Asia (n=8).[12-14,34-36,48,50-54] Most studies focused on the provision of on-going training for a particular disease group, the majority of which concentrated on child and maternal health issues (n=19),[32,34,38,44,46,48,50,52,56-59] or HIV and TB (n=7)[35,36,42,43,53,60,61] (*see Table 2, supplementary material).*

The reported frequency of on-going training delivery was highly variable, ranging from one off refresher training courses held over the period of one day,[62] to monthly refresher training sessions over the period of two years[33] (*see Table 2, supplementary material*). Most training was delivered in-person, with five studies reporting the use of mobile technologies as a

For peer review only - http://bmjopen.bmj.com/site/about/guidelines.xhtml

means of training delivery.[13,31,38,47,49] Only eight studies sought the views of CHWs or stakeholders in the process of programme design.[12,13,31,33,35,36,38,45]

To report the outcomes of on-going training programmes, a range of different measures were used. 13 studies evaluated the effect of on-going training by using proxy markers to assess change in practice. This included assessing behaviour change at a community level, such as improved vaccination uptake and hand washing amongst households,[12] to changes in practice among CHWs, such as improved record keeping.[38] Assessment of knowledge and skills, mainly through the use of pre- and post-intervention tests, formed the sole means of evaluation in seven of the studies.[32,54,58,60-63] Eight studies used a qualitative approach for programme evaluation, mainly through the use of interviews and focus discussion groups.[13,33,35,40,43,47,49,64] Eight studies adopted a mixed methods approach, combining an assessment of skills and knowledge with qualitative feedback,[31,39,52,53,57] or changes in behaviours and practice,[14,44] or a behaviour change and qualitative approach.[45]

For peer review only

Discussion

This scoping review highlights the diverse range of approaches in the design, delivery and reported outcomes of on-going training for CHWs in LMICs, as well as identifying critical gaps in the literature.

Location, content and duration of on-going training programmes

The majority of studies describing on-going training for CHWs have been concentrated in East Africa and South Asia. Given the highly contextualised role of the CHW,[65] this presents an opportunity for further research to be carried out in other geographical contexts. Furthermore, the majority of studies focused on the provision of on-going training for maternal and child health, or infectious diseases such as HIV and TB. Although these are undoubtedly major challenges in LMICs, no studies focussed on the provision of training for non-communicable diseases (NCDs) and only one study focussed on the provision of on-going training for CHWs involved in mental health care.[45] NCDs have been described as the "social justice issue of our time,"[66,67] since they disproportionally affect populations in LMICs.[68] It is therefore imperative that more attention is directed towards providing on-going training in the prevention and management of NCDs at a community level if we are to make realistic progress towards SDG 3.4, which has set the target of reducing premature mortality from NCDs by a third, by 2030.[69]

The on-going training programmes varied in terms of duration (*see Table 2, supplementary material*). For example, Zeitz et al., reported on a one-day refresher training course for CHWs which specifically focused on acute respiratory illness in children and used pre and post testing of knowledge as the outcome measure of the training.[62] This is in contrast with the study by Kawasaki et al., who carried out a two-year study where CHWs received monthly refresher trainings and the outcome measures were focused on behaviour change at

the community level; for example improved handwashing techniques and the number of household visits carried out by CHWs.[33] This variation between programmes, both in terms of duration, structure, and outcomes reporting, makes direct comparison difficult.

Participatory approaches to programme design

Oliver et al., highlighted the importance of co-designing programmes with CHWs to help ensure relevance to their practices and experiences.[70] Our scoping review revealed a lack of participatory input from key stakeholders in the design and delivery of the training programmes. Only eight studies documented seeking input from CHWs in the design of the training programmes. For example, Joos et al., modified the content and frequency of motivational SMS messages based on CHW feedback,[38] and Puchalski Ritchie et al., developed training content based on the needs identified by CHWs prior to the programme being established.[36] Similarly, McLean et al., modified the structure and content of supervisory sessions based on the feedback from CHWs to ensure terminology was clarified and printed copies of training resources were provided.[45] Research into the use of participatory action research (PAR) methodologies to inform the design of on-going training programmes for CHWs in LMICs is one area that would warrant further investigation. PAR broadly involves working 'with' end-users in a collaborative effort rather than 'for' or 'on' them.[71] It encapsulates the ideals of promoting autonomy and social justice, and works on the principle that the end-users wishes and needs, should be respected and valued.[71] This school of thought was echoed by Perry and Crigler, who advised a "top-down supervisory approach…may not be as feasible or effective as a participatory supervision model where CHWs and their communities are provided with the resources and autonomy to seek out the support that they need to perform well and stay motivated."[72]

For peer review only - http://bmjopen.bmj.com/site/about/guidelines.xhtml

It is also important to consider socio-cultural sensitivities in the design of an on-going training intervention, including cultural beliefs, especially in areas where the practice of traditional medicine is still commonplace and may be at odds with a more Western approach to healthcare. In the study by Singh et al., the training intervention was delayed by four months due to villagers believing the immunizations used by the CHWs were intended to cause infertility and the insecticide treated bednets were designed to 'kill their children'.[12] This is especially relevant when on-going training programmes are being designed and implemented by non-native researchers, in countries emerging from post-colonial pasts and where local beliefs are rooted in historical antecedants.[73]

Use of learning theory in programme design

Despite many on-going training programmes claiming to help improve knowledge of CHWs, only three studies used theories of learning to inform programme design.[13,31,32] Although Henry et al., made explicit reference to best supportive supervision practices using theories of feedback and problem solving, this commentary was often absent in other literature. There is therefore a danger that studies focusing on on-going training can over claim with regards to their ability to promote CHWs' education. Robertson et al., reported in their study that supervisory activities often involved checking reports, rather than focusing on developing more transferable skills, such as problem solving.[41] By focussing on an information dissemination model of learning, rather than underlying processes and theories, we are in danger of training CHWs to follow protocols and algorithms and not fully understand what they are practicing or the importance of the knowledge they have acquired; a concept that has been evidenced in previous studies.[74,75] This highlights the need for interdisciplinary work between global health researchers, practitioners and educationalists to help the creation and delivery of meaningful and pedagogically effective learning activities. In the study by Ndima

For peer review only

et al., many of the CHWs commented on how they found the checklist tool used during feedback demotivating. This method of monitoring meant they received very little meaningful feedback on how to improve their individual practice.[14] The literature to date on CHW training indicates that providing feedback is an important component of improving CHW performance,[76] yet many of the studies failed to provide specific details of how feedback was delivered. In the study by Rabbani et al., a CHW stated “feedback is very important, in this way our capabilities are exposed".[39] It was revealed that CHWs involved in this study preferred oral group feedback, rather than written individual feedback, since they felt that written feedback provided documentary evidence of “good and bad CHWs” and that their peers were unable to learn from their mistakes if feedback was not shared.[39]

Delivery of on-going training programmes

The majority of studies provided very few details regarding the structure and format of on-going training, however role-playing was a popular activity in several programmes.[32,35,36,59] Most programmes used local CHW supervisors and leaders to deliver training, rather than external trainers, which is important for sustainability and capacity building reasons.[41]

In terms of delivery style, most refresher training programmes and supervisions took place in-person. Only five studies reporting the use of mobile technologies as a delivery tool, and of these, two studies used different measures to report outcomes from the same project.[31,47] This was surprising since mobile technologies have been used as a mean to train other cadres of healthcare professionals in LMICs.[77-79] Given the high ownership of mobile phones in sub-Saharan Africa,[80] and the ability for flexible learning, data collection,[81] the use of mHealth to facilitate on-going training warrants exploration.[82] One of the studies included in this review highlighted the role of mobile phones to strengthen supportive supervision for CHWs in Kenya.[13] A WhatsApp group to facilitate instant messaging was created for CHWs and their

supervisors to "*support supervision, professional development, and team building*".[13] Importantly the authors of this study reported not only on the quality assurance and information exchange, which the system facilitated, but also on the supportive environment fostered by the use of the technology.[13] Another study to use mobile technologies as an on-going training tool found that there was no difference between the intervention and control groups in terms of knowledge acquisition.[31] As a caveat, Hampshire et al., have urged researchers and practitioners to proceed with caution and consider the financial implications when considering mobile technologies as a training tool for CHWs, due to the potential risk of reinforcing socioeconomic, geographical and gender inequalities.[83] Finally, Joos et al., highlighted the need to consider how mobile phones can successfully transition to scale following pilot studies.[38]

Reported outcomes of on-going training programmes

For outcome reporting, 16 studies used markers of behaviour change at the household level or CHW practice to measure the impact of on-going training. For example, Horwood et al. found that children managed by a CHW who had attended a refresher training session were more likely to be managed correctly according to iCCM guidelines compared to those who had not.[44] Similarly, a study by Singh et al., found that homes in areas where CHWs had received supportive supervision were more likely to have installed and functioning tippy taps for hand washing, compared to areas served by CHWs who had not received supervision.[12] Using measures of behaviour change to evaluate the effectiveness of on-going training is a welcome move towards ensuring meaningful programme evaulation,[84,85] however researchers and programme managers should be aware of the multiple confounding variables that could influence these behaviors, such as the Hawthorne effect, and the difficulty in assessing these practices longitudinally.

For peer review only

13 studies measured knowledge and skills acquisition and retention following on-going training.[31,32,52-54,57,58,61-63] This was largely done using pre- and post- intervention tests. Although pre- and post-tests are a popular way of conducting assessments of knowledge, they do not necessarily reflect the abilities of CHWs to perform their role well in the community,[86] nor do they provide any insight into CHWs experiences of training. Hamilton and Friesen, argue that instrumental views of assessing learning often fail to capture the practical and emancipatory concerns of learners,[87] and thus alternative methods of evaluation should be explored.[75] Furthermore, it is important to consider the validity and applicability of such tests to real life settings, given that many of the assessment tools have been designed by the researchers and are unvalidated. What is more, some CHWs have only been in formal education to the level of primary or secondary school, and so this form of assessment may introduce construct-validity bias.

Puchalski Richie et al., actively avoided using an “assessment of knowledge and skills”[35] since they were concerned that it might negatively affect participation in training; instead, they carried out a qualitative evaluation of CHWs overall satisfaction with the program as a measure of training success.[36] Interestingly, Rowe et al., who used a skills and knowledge assessment tool and found no improvement in scores between the groups of CHWs who took part in refresher training and those who did not.[54] They questioned the usefulness of refresher training based on this outcome, however failed to acknowledge the other benefits of on-going training which they did not measure, such as an improved sense of community, motivation and empowerment.[76]

No studies used the framework for outcome-level evaluation of in-service training of healthcare workers produced by O’Mallay et al., in 2013.[88] This framework was developed in a holistic manner to evaluate in-service training of health workers based on the needs of the

For peer review only

individual, the organisation and the health system. Current assessment of in-service training programme assessment relies heavily on measuring and reporting training "outputs" such as the number of CHWs trained, the total hours of training delivered, and scores obtained on standardised tests.

A small number of studies used self-reported satisfaction,[49] motivation[39] or increased agency[36] as outcomes to measure the impact of on-going training. These are what Kok refers to as "software" of a training programme and can affect motivation and performance.[89] Kok argues that the software elements of the system are important since they effect CHW performance by "influencing self-esteem, attitudes and agency,"[89] as well as satisfaction and motivation. Ndima et al., commented that when training focuses too heavily on developing technical skills there is a danger that "examining value and attitudes of CHWs and abilities to understand and support individual and group dynamics"[14] can be lost.

Study limitations

It is important to recognise that given the highly contextualised nature of CHW training programmes[20,65] this scoping review does not try to address best practice or provide guidelines. Rather, we have attempted to map the current landscape of on-going training for CHWs in order to broadly identify key similarities or differences between on-going training programmes and identify areas that may have received little attention in the literature to date to help inform other researchers, practitioners or policy makers working in this field.

We tried to be as inclusive as possible to identify relevant literature, but with the diverse range of terms used to describe CHWs, it is possible we have inadvertently missed out some eligible studies describing on-going training for CHWs.

For peer review only

Unfortunately, we had to exclude one study at the final inclusion stage, as although the abstract was in English and appeared to be relevant, the full text of the article was in French and we did not have sufficient resources to translate it.[90]

Finally, given the nature of scoping reviews, a critical appraisal of the studies included in the review was not performed.

For peer review only

For peer review only - http://bmjopen.bmj.com/site/about/guidelines.xhtml

Conclusions

There is significant variability between on-going training programmes for CHWs in LMICs, both in terms of design, content, duration and reported outcomes (*see Supplementary Material Figure 2*). This fragmented approach means little is understood about how to best deliver on-going training in settings of poverty and social inequality. On-going training programmes to date have largely focused on specific areas, for example child and maternal health and infectious diseases, in limited geographic contexts and have variable, fragmented approaches in measuring outcomes. The danger is that this approach fails to acknowledge what Kim, Farmer and Porter refer to as the "broader systems and conditions affecting global health-care delivery."[85]

Further research should be carried out to better understand how learning theories and alternative models of on-going training delivery, such as mobile technologies, could be leveraged to support and sustain effective training and educational programmes. In order to achieve this, collaboration between global health practitioners working across disciplines and organisations responsible for training CHWs is needed. By taking this interdisciplinary approach, on-going training which is designed and measured to consider contextual needs is more likely to contribute to a systems level improvement in resource limited settings.

For peer review only

Figure Legends:

Figure 1. PRISMA flow diagram. The PRIMSA diagram details our search and selection process applied during the scoping review.

Contributor Statement: Activities undertaken by the authors were as follows: Establishment of research question(s) and development of search strategy: JOD, IK and NW. Background framing: JOD & NW. Database search and record screening: JOD, COD & IK. Extraction of primary studies from the included reviews: JOD and COD. Discussion: JOD, COD, NW, SS.

Acknowledgements: We wish to thank Miss. Stephanie Sobek for proofreading the paper prior to final submission.

Data sharing: All data is contained within the main body of the text and in the on-line supplementary material.

For peer review only

References

1. Darzi A, Evans T. The global shortage of health workers-an opportunity to transform care. *Lancet.* 2016;388(10060):2576-2577.
2. Perry HB, Zulliger R, Rogers MM. Community health workers in low-, middle-, and high-income countries: an overview of their history, recent evolution, and current effectiveness. *Annu Rev Public Health.* 2014;35:399-421.
3. Turinawe EB, Rwemisisi JT, Musinguzi LK, et al. Selection and performance of village health teams (VHTs) in Uganda: lessons from the natural helper model of health promotion. *Hum Resour Health.* 2015;13:73.
4. Bhattacharyya K, Winch P, LeBan K, M. T. *Community Health Worker Incentives and Disincentives: How They Affect Motivation, Retention, and Sustainability. Basic Support for Institutionalizing Child Survival Project (BASICS II).* Virginia, USA: USAID;2001.
5. Prinja S, Jeet G, Verma R, et al. Economic analysis of delivering primary health care services through community health workers in 3 North Indian states. *PLoS One.* 2014;9(3):e91781.
6. Vaughan K, Kok MC, Witter S, Dieleman M. Costs and cost-effectiveness of community health workers: evidence from a literature review. *Hum Resour Health.* 2015;13:71.
7. The World Health Organization. Community health workers: What do we know about them? The state of the evidence on programmes, activities, costs and impact on health outcomes of using community health workers. 2007; http://www.who.int/hrh/documents/community_health_workers_brief.pdf Accessed 05/11/2017.
8. Li VC, Goethals PR, Dorfman S. A global review of training of community health workers, 1983-84. *Int Q Community Health Educ.* 2006;27(3):181-218.
9. Redick C, Faich Dini HS, Long L-A. *The Current State of CHW Training Programs in Sub-Saharan Africa and South Asia: What We Know, What We Don't Know, and What We Need to Do.* 2014.
10. Hopkins D. *Inservice training and educational development: An International Survey* New Hampshire, USA,1986.
11. The World Health Organization. Training for mid-level managers (MLM) Module 4: Supportive supervision. 2008; http://www.who.int/immunization/documents/MLM_module4.pdf. Accessed 05/11/2017.
12. Singh D, Negin J, Orach CG, Cumming R. Supportive supervision for volunteers to deliver reproductive health education: a cluster randomized trial. *Reproductive Health.* 2016;13(1):126.
13. Henry JV, Winters N, Lakati A, et al. Enhancing the Supervision of Community Health Workers With WhatsApp Mobile Messaging: Qualitative Findings From 2 Low-Resource Settings in Kenya. *Glob Health Sci Pract.* 2016;4(2):311-325.
14. Ndima SD, Sidat M, Give C, Ormel H, Kok MC, Taegtmeyer M. Supervision of community health workers in Mozambique: a qualitative study of factors influencing motivation and programme implementation. *Human Resources for Health [Electronic Resource].* 2015;13:63.
15. Crigler L HK, Furth R, Bjerregaard D. Community Health Worker Assessment and Improvement Matrix (CHW AIM): A Toolkit for Improving Community Health Worker Programs and Services USAID Health Care Improvement Project. 2011; http://www.who.int/workforcealliance/knowledge/toolkit/50.pdf. Accessed 17/8/2017.
16. Wanduru P, Tetui M, Tuhebwe D, et al. The performance of community health workers in the management of multiple childhood infectious diseases in Lira, northern Uganda - a mixed methods cross-sectional study. *Glob Health Action.* 2016;9:33194.

For peer review only - http://bmjopen.bmj.com/site/about/guidelines.xhtml

17. Stellenberg E, Van Zyl M, Eygelaar J. Knowledge of community care workers about key family practices in a rural community in South Africa. *African Journal of Primary Health Care & Family Medicine.* 2015;7(1):892.
18. Sipsma HL, Curry LA, Kakoma JB, Linnander EL, Bradley EH. Identifying characteristics associated with performing recommended practices in maternal and newborn care among health facilities in Rwanda: a cross-sectional study. *Human Resources for Health.* 2012;10.
19. Sheikh S, Qureshi RN, Khowaja AR, et al. Health care provider knowledge and routine management of pre-eclampsia in Pakistan. *Reproductive Health.* 2016;13(Suppl 2):104.
20. Blacklock C, Goncalves Bradley DC, Mickan S, et al. Impact of Contextual Factors on the Effect of Interventions to Improve Health Worker Performance in Sub-Saharan Africa: Review of Randomised Clinical Trials. *PLoS One.* 2016;11(1):e0145206.
21. Leslie HH, Gage A, Nsona H, Hirschhorn LR, Kruk ME. Training And Supervision Did Not Meaningfully Improve Quality Of Care For Pregnant Women Or Sick Children In Sub-Saharan Africa. *Health Affairs.* 2016;35(9):1716-1724.
22. Bluestone J, Johnson P, Fullerton J, Carr C, Alderman J, BonTempo J. Effective in-service training design and delivery: evidence from an integrative literature review. *Hum Resour Health.* 2013;11:51.
23. Hill Z, Dumbaugh M, Benton L, et al. Supervising community health workers in low-income countries--a review of impact and implementation issues. *Glob Health Action.* 2014;7:24085.
24. Colquhoun HL, Levac D, O'Brien KK, et al. Scoping reviews: time for clarity in definition, methods, and reporting. *J Clin Epidemiol.* 2014;67(12):1291-1294.
25. Arksey H, O'Malley L. Scoping studies: towards a methodological framework. *International Journal of Social Research Methodology.* 2005;8(1):19-32.
26. Miake-Lye IM, Hempel S, Shanman R, Shekelle PG. What is an evidence map? A systematic review of published evidence maps and their definitions, methods, and products. *Syst Rev.* 2016;5:28.
27. Haby MM, Chapman E, Clark R, Barreto J, Reveiz L, Lavis JN. What are the best methodologies for rapid reviews of the research evidence for evidence-informed decision making in health policy and practice: a rapid review. *Health Res Policy Syst.* 2016;14(1):83.
28. The World Bank. World Bank Country and Lending Groups. 2012 http://data.worldbank.org/about/country-classifications. Accessed 07/11/2017.
29. The World Health Organization. *Strengthening the performance of community health workers in primary health care. Report of a WHO Study Group.* WHO Technical Report Series, No. 780 Geneva World Health Organization 1989.
30. Lewin SA, Dick J, Pond P, et al. Lay health workers in primary and community health care. *Cochrane Database Syst Rev.* 2005(1):CD004015.
31. Gill CJ, Ngoc BL, Halim N, et al. The mCME Project: A Randomized controlled trial of an SMS-Based continuing medical education intervention for improving medical knowledge among Vietnamese community based physicians' assistants. *PLoS ONE.* 2016;11 (11) (no pagination)(e0166293).
32. Gupta M, Tripathy JP, Jamir L, Sarwa A, Sinha S, Bhag C. Improving quality of home-based postnatal care by microteaching of multipurpose workers in rural and urban slum areas of Chandigarh, India: a pilot study. *Advances in Medical Education and Practice.* 2017;8:1-8.
33. Kawasaki R, Sadamori T, Ferreira de Almeida T, et al. Reactions of community members regarding community health workers' activities as a measure of the impact of a training program in Amazonas, Brazil. *Journal of Rural Medicine.* 2015;10(1):7-19.
34. Kuule Y, Dobson AE, Woldeyohannes D, et al. Community Health Volunteers in Primary Healthcare in Rural Uganda: Factors Influencing Performance. *Frontiers in Public Health.* 2017;5:62.

For peer review only

35. Puchalski Ritchie LM, Schull MJ, Martiniuk AL, et al. A knowledge translation intervention to improve tuberculosis care and outcomes in Malawi: a pragmatic cluster randomized controlled trial. *Implement Sci.* 2015;10:38.
36. Puchalski Ritchie LM, van Lettow M, Barnsley J, et al. Lay Health Workers experience of a tailored knowledge translation intervention to improve job skills and knowledge: a qualitative study in Zomba district Malawi. *BMC Medical Education.* 2016;16:54.
37. Das A, Friedman J, Kandpal E, et al. Strengthening malaria service delivery through supportive supervision and community mobilization in an endemic Indian setting: an evaluation of nested delivery models. *Malar J.* 2014;13:482.
38. Joos O, Silva R, Amouzou A, et al. Evaluation of a mHealth Data Quality Intervention to Improve Documentation of Pregnancy Outcomes by Health Surveillance Assistants in Malawi: A Cluster Randomized Trial. *PLoS One.* 2016;11(1):e0145238.
39. Rabbani F, Perveen S, Aftab W, Zahidie A, Sangrasi K, Qazi SA. Health workers' perspectives, knowledge and skills regarding community case management of childhood diarrhoea and pneumonia: a qualitative inquiry for an implementation research project "Nigraan" in District Badin, Sindh, Pakistan. *BMC Health Serv Res.* 2016;16:462.
40. Rabbani F, Shipton L, Aftab W, Sangrasi K, Perveen S, Zahidie A. Inspiring health worker motivation with supportive supervision: a survey of lady health supervisor motivating factors in rural Pakistan. *BMC Health Serv Res.* 2016;16(1):397.
41. Roberton T, Applegate J, Lefevre AE, et al. Initial experiences and innovations in supervising community health workers for maternal, newborn, and child health in Morogoro region, Tanzania. *Hum Resour Health.* 2015;13:19.
42. Adejumo AO, Azuogu B, Okorie O, et al. Community referral for presumptive TB in Nigeria: a comparison of four models of active case finding. *BMC Public Health.* 2016;16:177.
43. Datiko DG, Yassin MA, Tulloch O, et al. Exploring providers' perspectives of a community based TB approach in Southern Ethiopia: implication for community based approaches. *BMC Health Serv Res.* 2015;15:501.
44. Horwood C, Butler L, Barker P, et al. A continuous quality improvement intervention to improve the effectiveness of community health workers providing care to mothers and children: a cluster randomised controlled trial in South Africa. *Hum Resour Health.* 2017;15(1):39.
45. McLean KE, Kaiser BN, Hagaman AK, Wagenaar BH, Therosme TP, Kohrt BA. Task sharing in rural Haiti: Qualitative assessment of a brief, structured training with and without apprenticeship supervision for community health workers. *Intervention (Amstelveen).* 2015;13(2):135-155.
46. Miller NP, Amouzou A, Hazel E, et al. Assessment of the impact of quality improvement interventions on the quality of sick child care provided by Health Extension Workers in Ethiopia. *J Glob Health.* 2016;6(2):020404.
47. Sabin LL, Larson Williams A, Le BN, et al. Benefits and Limitations of Text Messages to Stimulate Higher Learning Among Community Providers: Participants' Views of an mHealth Intervention to Support Continuing Medical Education in Vietnam. *Glob Health Sci Pract.* 2017;5(2):261-273.
48. Talukder S, Farhana D, Vitta B, Greiner T. In a rural area of Bangladesh, traditional birth attendant training improved early infant feeding practices: a pragmatic cluster randomized trial. *Matern Child Nutr.* 2017;13(1).
49. Vallieres F, McAuliffe E, van Bavel B, Wall PJ, Trye A. There's No App for That: Assessing the Impact of mHealth on the Supervision, Motivation, Engagement, and Satisfaction of Community Health Workers in Sierra Leone. *Ann Glob Health.* 2016;82(5):936-949.
50. Ameha A, Karim AM, Erbo A, et al. Effectiveness of supportive supervision on the consistency of integrated community cases management skills of the health extension workers in 113 districts of Ethiopia. *Ethiopian Medical Journal.* 2014;52 Suppl 3:65-71.

For peer review only - http://bmjopen.bmj.com/site/about/guidelines.xhtml

51. Ayele F, Desta A, Larson C. The functional status of community health agents: A trial of refresher courses and regular supervision. *Health Policy and Planning.* 1993;8(4):379-384.
52. Gallo MF, Walldorf J, Kolesar R, et al. Evaluation of a volunteer community-based health worker program for providing contraceptive services in Madagascar. *Contraception.* 2013;88(5):657-665.
53. Msisuka C, Nozaki I, Kakimoto K, Seko M, Ulaya MM. An evaluation of a refresher training intervention for HIV lay counsellors in Chongwe District, Zambia. *SAHARA J: Journal of Social Aspects of HIV/AIDS Research Alliance.* 2011;8(4):204-209.
54. Rowe SY, Olewe MA, Kleinbaum DG, et al. Longitudinal analysis of community health workers' adherence to treatment guidelines, Siaya, Kenya, 1997-2002. *Tropical Medicine and International Health.* 2007;12(5):651-663.
55. Mengistu B, Karim AM, Eniyew A, et al. Effect of performance review and clinical mentoring meetings (PRCMM) on recording of community case management by health extension workers in Ethiopia. *Ethiop Med J.* 2014;52 Suppl 3:73-81.
56. Mkumbo E, Hanson C, Penfold S, Manzi F, Schellenberg J. Innovation in supervision and support of community health workers for better newborn survival in southern Tanzania. *Int Health.* 2014;6(4):339-341.
57. Carlough M, McCall M. Skilled birth attendance: What does it mean and how can it be measured? A clinical skills assessment of maternal and child health workers in Nepal. *International Journal of Gynecology & Obstetrics.* 2005;89(2):200-208.
58. Gupta DN, SenGupta PG, Sircar BK, Mondal S, Sarkar S, Deb BC. Implementation of ORT: some problems encountered in training of health workers during an operational research programme. *Indian Journal of Public Health.* 1994;38(2):69-72.
59. Hadi A. Management of acute respiratory infections by community health volunteers: experience of Bangladesh Rural Advancement Committee (BRAC). *Bulletin of the World Health Organization.* 2003;81(3):183-189.
60. Dewing S, Mathews C, Cloete A, Schaay N, Simbayi L, Louw J. Lay counselors' ability to deliver counseling for behavior change. *J Consult Clin Psychol.* 2014;82(1):19-29.
61. Mash R, Baldassini G, Mkhatshwa H, Sayeed I, Ndapeua S, Mash B. Reflections on the training of counsellors in motivational interviewing for programmes for the prevention of mother to child transmission of HIV in sub-Saharan Africa. *South African Family Practice.* 2008;50(2):53-59.
62. Zeitz PS, Harrison LH, Lopez M, Cornale G. Community health worker competency in managing acute respiratory infections of childhood in Bolivia. *Bull Pan Am Health Organ.* 1993;27(2):109-119.
63. Kumar S, Roy R, Dutta S. Scaling-up public sector childhood diarrhea management program: Lessons from Indian states of Gujarat, Uttar Pradesh and Bihar. *Journal of Global Health.* 2015;5(2):020414.
64. Javanparast S, Baum F, Labonte R, Sanders D, Rajabi Z, Heidari G. The experience of community health workers training in Iran: a qualitative study. *BMC Health Services Research.* 2012;12:291.
65. Dieleman M, Gerretsen B, van der Wilt GJ. Human resource management interventions to improve health workers' performance in low and middle income countries: a realist review. *Health Res Policy Syst.* 2009;7:7.
66. Saligram N, Nugent R. Chronic diseases--the social justice issue of our time. *Lancet.* 2016;387(10022):943.
67. Casswell S. Chronic diseases--the social justice issue of our time. *Lancet.* 2016;387(10022):942-943.
68. The World Health Organization. Global Health Observatory data - NCD mortality and morbidity. 2015 http://www.who.int/gho/ncd/mortality_morbidity/en/. Accessed 14/11/2017.

For peer review only

69. United Nations Development Programme. Sustainable Development Goals - SDG 3. 2017 https://sustainabledevelopment.un.org/sdg3. Accessed 14/11/2017
70. Oliver M, Geniets A, Winters N, Rega I, Mbae SM. What do community health workers have to say about their work, and how can this inform improved programme design? A case study with CHWs within Kenya. *Glob Health Action.* 2015;8:27168.
71. Bennett M. A Review of the Literature on the Benefits and Drawbacks of Participatory Action Research *First Peoples Child & Family Review.* 2004;1(1):19-32.
72. Perry H, Crigler L. *Developing and Strengthening Community Health Worker Programs at Scale* Washington D.C. : USAID and MCHIP (Maternal and Child Health Integrated Program);2014.
73. Grietens KP, Ribera JM, Erhart A, et al. Doctors and vampires in sub-Saharan Africa: ethical challenges in clinical trial research. *Am J Trop Med Hyg.* 2014;91(2):213-215.
74. Jones CO, Wasunna B, Sudoi R, Githinji S, Snow RW, Zurovac D. "Even if you know everything you can forget": health worker perceptions of mobile phone text-messaging to improve malaria case-management in Kenya. *PLoS One.* 2012;7(6):e38636.
75. Winters N, Oliver M, Langer L. Can mobile health training meet the challenge of 'measuring better'? *Comparitive Education.* 2017;53(1):115-131.
76. Rowe AK, de Savigny D, Lanata CF, Victora CG. How can we achieve and maintain high-quality performance of health workers in low-resource settings? *Lancet.* 2005;366(9490):1026-1035.
77. O'Donovan J, Bersin A, O'Donovan C. The effectiveness of mobile health (mHealth) technologies to train healthcare professionals in developing countries: a review of the literature. *BMJ Innovations.* 2015;1(1):33-36.
78. Singh P, Sachs JD. 1 million community health workers in sub-Saharan Africa by 2015. *Lancet.* 2013;382(9889):363-365.
79. Diedhiou A, Gilroy KE, Cox CM, et al. Successful mLearning Pilot in Senegal: Delivering Family Planning Refresher Training Using Interactive Voice Response and SMS. *Glob Health Sci Pract.* 2015;3(2):305-321.
80. GSMA Intelligence. The Mobile Economy, SubSaharan Africa 2015. 2015; https://www.gsma.com/mobileeconomy/archive/GSMA_ME_SubSaharanAfrica_2015.pdf. Accessed 23/11/2017.
81. Gordon N. Flexible Pedagogies: technology-enhanced learning. 2014; https://www.heacademy.ac.uk/system/files/resources/tel_report_0.pdf. Accessed 21/11/2017.
82. Langer L., Winters N., Stewart R. Mobile Learning for Development: Ready to Randomise? In: Kalz M., Bayyurt Y., Specht M., eds. *Mobile as a Mainstream – Towards Future Challenges in Mobile Learning. mLearn 2014. Communications in Computer and Information Science*: Springer, Cham 2014.
83. Hampshire K, Porter G, Mariwah S, et al. Who bears the cost of 'informal mhealth'? Health-workers' mobile phone practices and associated political-moral economies of care in Ghana and Malawi. *Health Policy Plan.* 2017;32(1):34-42.
84. A new institute for global health evaluations. *Lancet.* 2007;369(9577):1902.
85. Kim JY, Farmer P, Porter ME. Redefining global health-care delivery. *Lancet.* 2013;382(9897):1060-1069.
86. Abdel-All M, Putica B, Praveen D, Abimbola S, Joshi R. Effectiveness of community health worker training programmes for cardiovascular disease management in low-income and middle-income countries: a systematic review. *BMJ Open.* 2017;7(11):e015529.
87. Hamilton E, Friesen N. Online Education: A Science and Technology Studies Perspective. *Canadian Journal of Learning and Technology.* 2013;39(2).
88. O'Malley G, Perdue T, Petracca F. A framework for outcome-level evaluation of in-service training of health care workers. *Human Resources for Health.* 2013;11.

For peer review only

89. Kok MC, Broerse JEW, Theobald S, Ormel H, Dieleman M, Taegtmeyer M. Performance of community health workers: situating their intermediary position within complex adaptive health systems. *Hum Resour Health.* 2017;15(1):59.
90. Sylla A, Guèye EHB, N'Diaye O, et al. Low level educated community health workers training: a strategy to improve children access to acute respiratory treatment in Senegal. *Archives de Pediatrie.* 2007;14(3):244-248.

For peer review only

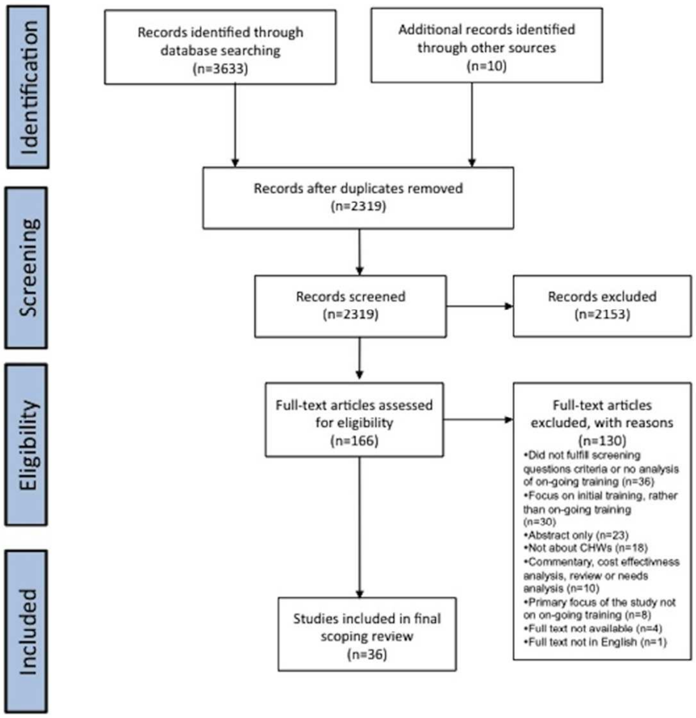

Figure 1. PRISMA flow diagram. The PRIMSA diagram details our search and selection process applied during the scoping review.

81x60mm (300 x 300 DPI)

Supplementary Material

Search results and search strategies.

The following search terms were used and adapted as appropriate for each database on the 10th of July 2017:

Database	Number of hits	Page number for search details
Medline, Embase and Global Health and AMED via (OVID)	1253	2-4
Scopus	1217	5-6
Web of Science	1001	7-9
ASSIA via ProQuest	64	10
LILACS	21	11-12
BEI via EBSCO	1	13-16
ERIC via EBSCO	9	13-16
CINAHL via EBSCO	67	13-16

Total number of references generated from original search: 3633

Number of duplicate references: 1314

Total number of references to screen by title and abstract after de-duplication: 2319

Total number of studies to be review at full text stage: 166

Final number of studies to include in final review after full text screen: 36

For peer review only - http://bmjopen.bmj.com/site/about/guidelines.xhtml

AMED (Allied and Complementary Medicine) 1985 to July 2017, **Embase** 1974 to 2017 July 10, **Global Health** 1973 to 2017 Week 29 **Ovid MEDLINE(R)** 1946 to July Week 2 2017.

1. Developing Countries**.sh,kf.**

2. (Africa or Asia or Caribbean or West Indies or South America or Latin America or Central America)**.hw,kf,ti,ab,cp.**

3. (Afghanistan or Albania or Algeria or Angola or Antigua or Barbuda or Argentina or Armenia or Armenian or Aruba or Azerbaijan or Bahrain or Bangladesh or Barbados or Benin or Byelarus or Byelorussian or Belarus or Belorussian or Belorussia or Belize or Bhutan or Bolivia or Bosnia or Herzegovina or Hercegovina or Botswana or Brasil or Brazil or Bulgaria or Burkina Faso or Burkina Fasso or Upper Volta or Burundi or Urundi or Cambodia or Khmer Republic or Kampuchea or Cameroon or Cameroons or Cameron or Camerons or Cape Verde or Central African Republic or Chad or Chile or China or Colombia or Comoros or Comoro Islands or Comores or Mayotte or Congo or Zaire or Costa Rica or Cote d'Ivoire or Ivory Coast or Croatia or Cuba or Cyprus or Czechoslovakia or Czech Republic or Slovakia or Slovak Republic or Djibouti or French Somaliland or Dominica or Dominican Republic or East Timor or East Timur or Timor Leste or Ecuador or Egypt or United Arab Republic or El Salvador or Eritrea or Estonia or Ethiopia or Fiji or Gabon or Gabonese Republic or Gambia or Gaza or Georgia Republic or Georgian Republic or Ghana or Gold Coast or Greece or Grenada or Guatemala or Guinea or Guam or Guiana or Guyana or Haiti or Honduras or Hungary or India or Maldives or Indonesia or Iran or Iraq or Isle of Man or Jamaica or Jordan or Kazakhstan or Kazakh or Kenya or Kiribati or Korea or Kosovo or Kyrgyzstan or Kirghizia or Kyrgyz Republic or Kirghiz or Kirgizstan or Lao PDR or Laos or Latvia or Lebanon or Lesotho or Basutoland or Liberia or Libya or Lithuania or Macedonia or Madagascar or Malagasy Republic or Malaysia or Malaya or Malay or Sabah or Sarawak or Malawi or Nyasaland or Mali or Malta or Marshall Islands or Mauritania or Mauritius or Agalega Islands or Mexico or Micronesia or Middle East or Moldova or Moldovia or Moldovian or Mongolia or Montenegro or Morocco or Ifni or Mozambique or Myanmar or Myanma or Burma or Namibia or Nepal or Netherlands Antilles or New Caledonia or Nicaragua or Niger or Nigeria or Northern Mariana Islands or Oman or Muscat or Pakistan or Palau or Palestine or Panama or Paraguay or Peru or Philippines or Philipines or Phillipines or Phillippines or Poland or Portugal or Puerto Rico or Romania or Rumania or Roumania or Russia or Russian or Rwanda or Ruanda or Saint Kitts or St Kitts or Nevis or

For peer review only - http://bmjopen.bmj.com/site/about/guidelines.xhtml

Saint Lucia or St Lucia or Saint Vincent or St Vincent or Grenadines or Samoa or Samoan Islands or Navigator Island or Navigator Islands or Sao Tome or Saudi Arabia or Senegal or Serbia or Montenegro or Seychelles or Sierra Leone or Slovenia or Sri Lanka or Ceylon or Solomon Islands or Somalia or South Africa or Sudan or Suriname or Surinam or Swaziland or Syria or Tajikistan or Tadzhikistan or Tadjikistan or Tadzhik or Tanzania or Thailand or Togo or Togolese Republic or Tonga or Trinidad or Tobago or Tunisia or Turkey or Turkmenistan or Turkmen or Uganda or Ukraine or Uruguay or USSR or Soviet Union or Union of Soviet Socialist Republics or Uzbekistan or Uzbek or Vanuatu or New Hebrides or Venezuela or Vietnam or Viet Nam or West Bank or Yemen or Yugoslavia or Zambia or Zimbabwe or Rhodesia)**.hw,kf,ti,ab,cp.**

4. ((developing or less* developed or under developed or underdeveloped or middle income or low* income or underserved or under served or deprived or poor*) adj (countr* or nation? or population? or world))**.ti,ab.**

5. ((developing or less* developed or under developed or underdeveloped or middle income or low* income) adj (economy or economies))**.ti,ab.**

6. (low* adj (gdp or gnp or gross domestic or gross national))**.ti,ab.**

7. (low adj3 middle adj3 countr*)**.ti,ab.**

8. (lmic or lmics or third world or lami countr*)**.ti,ab.**

9. transitional countr***.ti,ab.**

10. or/1-9

AND

11. ((refresher* adj (train* or course*)) or (adequa* adj2 train*) or (on-going training) or (on-going education) or (continuing education) or ((in-service or update or recap*) adj3 train*)) or (exp education, continuing/ or exp inservice training/) or (supervision) or (supportive supervis*)

AND

12. ((community adj health* adj3 (worker* or volunteer or aide* or practition*)) or (community adj (mental health*) adj3 (worker* or volunteer or aide*)) or (village adj health* adj (worker* or team* or guide*)) or (lady health worker*) or (lady health visitor*) or (front-line primary health?care) or (front-line primary health care) or behvarz or brigadista or manzaneras or (rural health assistant*) or gramsakhi or (lay health worker*) or (trained birth assistant*) or (accredited social health activist*) or (adherence support worker*) or (care facilitator*) or (community adj10 (treatment support*)) or (community* adj4 (distributor* or

For peer review only

volunteer*)) or (health extension worker*) or (lay counsellor*) or (maternal health worker*) or (shasthy? shebikas) or (shasthy? kormis) or (front line primary health* care worker) or (front line primary healthcare worker*) or (health activist*)).ti,ab.

For peer review only

SCOPUS

(TITLE-ABS-KEY ((front-line AND primary AND healthcare) OR (front-line AND primary AND health AND care) OR behvarz OR brigadista OR manzaneras OR (rural AND health AND assistant*) OR gramsakhi OR (lay AND health AND worker*) OR (trained AND birth AND assistant*) OR (accredited AND social AND health AND activist*) OR (adherence AND support AND worker*) OR (care AND facilitator*))) OR (TITLE-ABS-KEY ((community AND health* W/3 (worker* OR volunteer OR aide* OR practit*)) OR (community AND mental AND health* W/3 (worker* OR volunteer OR aide* OR practit*)) OR (village AND health* W/1 (worker* OR team* OR guide*)) OR (lady AND health AND worker*) OR (lady AND health AND visitor*) OR (lay AND healthworker) OR (TITLE-ABS-KEY ((health AND extension AND worker*) OR (lay AND counsellor*) OR (maternal AND health AND worker*) OR (peer AND educator*) OR (shasthy* AND shebikas) OR (shasthy* AND kormi) OR (front AND line AND primary AND health* AND care AND worker) OR (front AND line AND primary AND healthcare AND worker*) OR (health AND activist*))) OR (TITLE-ABS-KEY (community W/10 treatment AND support*)) OR (TITLE-ABS-KEY (community W/10 distributor*)) OR (TITLE-ABS-KEY (community W/10 volunteer*)) OR (TITLE-ABS-KEY (community W/10 care AND worker*)) OR (TITLE-ABS-KEY (community W/10 health AND worker*))

AND

(TITLE-ABS-KEY (refresher AND train* OR refresher AND course* OR (adequa* W/2 train*) OR (on-going education) OR (on-going training) OR (continuing education) OR (continuing training) OR (recap training) OR (in-service training) OR (update training) OR (supervision) or (supportive supervision)

Limits applied

1. Countries - (Afghanistan or Albania or Algeria or Angola or Antigua or Barbuda or Argentina or Armenia or Armenian or Aruba or Azerbaijan or Bahrain or Bangladesh or Barbados or Benin or Byelarus or Byelorussian or Belarus or Belorussian or Belorussia or Belize or Bhutan or Bolivia or Bosnia or Herzegovina or Hercegovina or Botswana or Brasil or Brazil or Bulgaria or Burkina Faso or Burkina Fasso or Upper Volta or Burundi or Urundi or Cambodia or Khmer Republic or Kampuchea or Cameroon or Cameroons or Cameron or Camerons or Cape Verde or Central African Republic or Chad or Chile or China or Colombia or Comoros or Comoro Islands or Comores or Mayotte or Congo or Zaire or Costa Rica or Cote d'Ivoire or Ivory Coast or Croatia or Cuba or Cyprus or Czechoslovakia or Czech Republic or Slovakia or

For peer review only - http://bmjopen.bmj.com/site/about/guidelines.xhtml

Slovak Republic or Djibouti or French Somaliland or Dominica or Dominican Republic or East Timor or East Timur or Timor Leste or Ecuador or Egypt or United Arab Republic or El Salvador or Eritrea or Estonia or Ethiopia or Fiji or Gabon or Gabonese Republic or Gambia or Gaza or Georgia Republic or Georgian Republic or Ghana or Gold Coast or Greece or Grenada or Guatemala or Guinea or Guam or Guiana or Guyana or Haiti or Honduras or Hungary or India or Maldives or Indonesia or Iran or Iraq or Isle of Man or Jamaica or Jordan or Kazakhstan or Kazakh or Kenya or Kiribati or Korea or Kosovo or Kyrgyzstan or Kirghizia or Kyrgyz Republic or Kirghiz or Kirgizstan or Lao PDR or Laos or Latvia or Lebanon or Lesotho or Basutoland or Liberia or Libya or Lithuania or Macedonia or Madagascar or Malagasy Republic or Malaysia or Malaya or Malay or Sabah or Sarawak or Malawi or Nyasaland or Mali or Malta or Marshall Islands or Mauritania or Mauritius or Agalega Islands or Mexico or Micronesia or Middle East or Moldova or Moldovia or Moldovian or Mongolia or Montenegro or Morocco or Ifni or Mozambique or Myanmar or Myanma or Burma or Namibia or Nepal or Netherlands Antilles or New Caledonia or Nicaragua or Niger or Nigeria or Northern Mariana Islands or Oman or Muscat or Pakistan or Palau or Palestine or Panama or Paraguay or Peru or Philippines or Philipines or Phillipines or Phillippines or Poland or Portugal or Puerto Rico or Romania or Rumania or Roumania or Russia or Russian or Rwanda or Ruanda or Saint Kitts or St Kitts or Nevis or Saint Lucia or St Lucia or Saint Vincent or St Vincent or Grenadines or Samoa or Samoan Islands or Navigator Island or Navigator Islands or Sao Tome or Saudi Arabia or Senegal or Serbia or Montenegro or Seychelles or Sierra Leone or Slovenia or Sri Lanka or Ceylon or Solomon Islands or Somalia or South Africa or Sudan or Suriname or Surinam or Swaziland or Syria or Tajikistan or Tadzhikistan or Tadjikistan or Tadzhik or Tanzania or Thailand or Togo or Togolese Republic or Tonga or Trinidad or Tobago or Tunisia or Turkey or Turkmenistan or Turkmen or Uganda or Ukraine or Uruguay or USSR or Soviet Union or Union of Soviet Socialist Republics or Uzbekistan or Uzbek or Vanuatu or New Hebrides or Venezuela or Vietnam or Viet Nam or West Bank or Yemen or Yugoslavia or Zambia or Zimbabwe or Rhodesia)

2. Dates - (1978-2017)

For peer review only - http://bmjopen.bmj.com/site/about/guidelines.xhtml

Web of Science

# 25	1,001	#24 AND #16 AND #15<br>Indexes=SCI-EXPANDED, SSCI, A&HCI, CPCI-S, CPCI-SSH, BKCI-S, BKCI-SSH, ESCI, CCR-EXPANDED, IC Timespan=1978-2017
# 24	32,232	#23 OR #22 OR #21 OR #20 OR #19 OR #18 OR #17<br>Indexes=SCI-EXPANDED, SSCI, A&HCI, CPCI-S, CPCI-SSH, BKCI-S, BKCI-SSH, ESCI, CCR-EXPANDED, IC Timespan=1978-2017
# 23	3,562	TS=((health extension worker*) or (lay counsellor*) or (maternal health worker*) or (shasthy* kormis) or (shasthy* shebikas) or (health activist*))<br>Indexes=SCI-EXPANDED, SSCI, A&HCI, CPCI-S, CPCI-SSH, BKCI-S, BKCI-SSH, ESCI, CCR-EXPANDED, IC Timespan=1978-2017
# 22	20,258	TS=(community SAME treatment support*) OR TS=(community* NEAR/4 distributor*) OR TS=(community* NEAR/4 volunteer*) OR TS= (community* NEAR/4 care worker*) OR TS=(community* NEAR/4 careworker*) OR TS=(community* NEAR/4 healthworker*) OR TS=(community* NEAR/4 health worker*)<br>Indexes=SCI-EXPANDED, SSCI, A&HCI, CPCI-S, CPCI-SSH, BKCI-S, BKCI-SSH, ESCI, CCR-EXPANDED, IC Timespan=1978-2017
# 21	6,580	TS=((accredited social health activist*) or (adherence support worker*) or (care facilitator*))<br>Indexes=SCI-EXPANDED, SSCI, A&HCI, CPCI-S, CPCI-SSH, BKCI-S, BKCI-SSH, ESCI, CCR-EXPANDED, IC Timespan=1978-2017
# 20	1,606	TS=(behvarz or brigadista or manzaneras or (rural health assistant*) or gramsakhi or (lay health worker*) or (trained birth assistant*))<br>Indexes=SCI-EXPANDED, SSCI, A&HCI, CPCI-S, CPCI-SSH, BKCI-S, BKCI-SSH, ESCI, CCR-EXPANDED, IC Timespan=1978-2017
# 19	437	Ts=(frontline primary healthcare*) OR TS=(front line primary healthcare*) OR TS=(frontline primary health care*) OR TS=(front line primary health care*)<br>Indexes=SCI-EXPANDED, SSCI, A&HCI, CPCI-S, CPCI-SSH, BKCI-S, BKCI-SSH, ESCI, CCR-EXPANDED, IC Timespan=1978-2017
# 18	1,676	TS=(village health* worker*) OR TS=(village health* guide*) OR TS=(village health* team) OR TS=(lady health worker*)<br>Indexes=SCI-EXPANDED, SSCI, A&HCI, CPCI-S, CPCI-SSH, BKCI-S, BKCI-SSH, ESCI, CCR-EXPANDED, IC Timespan=1978-2017
#	3,502	TS=(community health* NEAR/3 worker*) OR TS=(community health* NEAR/3

For peer review only - http://bmjopen.bmj.com/site/about/guidelines.xhtml

17		volunteer*) OR TS=(community health* NEAR/3 aide*) OR TS=(community health* NEAR/3 practit*) OR TS=(community mental health* NEAR/3 worker*) OR TS=(community mental health* NEAR/3 volunteer*) OR TS=(community mental health* NEAR/3 aide*)<br>Indexes=SCI-EXPANDED, SSCI, A&HCI, CPCI-S, CPCI-SSH, BKCI-S, BKCI-SSH, ESCI, CCR-EXPANDED, IC Timespan=1978-2017
# 16	98,552	#14 OR #13 OR #12<br>Indexes=SCI-EXPANDED, SSCI, A&HCI, CPCI-S, CPCI-SSH, BKCI-S, BKCI-SSH, ESCI, CCR-EXPANDED, IC Timespan=1978-2017
# 15	3,687,339	#11 OR #10 OR #9 OR #8 OR #7 OR #6 OR #5 OR #4 OR #3 OR #2 OR #1<br>Indexes=SCI-EXPANDED, SSCI, A&HCI, CPCI-S, CPCI-SSH, BKCI-S, BKCI-SSH, ESCI, CCR-EXPANDED, IC Timespan=1978-2017
# 14	42,703	TS=(recap* NEAR/3 train*) OR TS=(update NEAR/3 train*) OR TS=(ongoing NEAR/3 train*) OR TS=(in-service NEAR/3 train*) OR TS=(on-going education) OR TS=(supervision) OR TS=(supportive supervision)<br>Indexes=SCI-EXPANDED, SSCI, A&HCI, CPCI-S, CPCI-SSH, BKCI-S, BKCI-SSH, ESCI, CCR-EXPANDED, IC Timespan=1978-2017
# 13	53,039	TS=(continuing education) OR TS=(continuing training)<br>Indexes=SCI-EXPANDED, SSCI, A&HCI, CPCI-S, CPCI-SSH, BKCI-S, BKCI-SSH, ESCI, CCR-EXPANDED, IC Timespan=1978-2017
# 12	4,506	TS=(refresher train* or refresher course*) OR TS=(adequa* NEAR/2 train*)<br>Indexes=SCI-EXPANDED, SSCI, A&HCI, CPCI-S, CPCI-SSH, BKCI-S, BKCI-SSH, ESCI, CCR-EXPANDED, IC Timespan=1978-2017
# 11	141,978	TS=(underserved population* or underserved world* or underserved countr* or underserved nation*) OR TS=(under served population* or under served world* or under served countr* or under served nation*) OR TS=(deprived population* or deprived world* or deprived countr* or deprived nation*) OR TS=(poor population* or poor world* or poorcountr* or poor nation*)<br>Indexes=SCI-EXPANDED, SSCI, A&HCI, CPCI-S, CPCI-SSH, BKCI-S, BKCI-SSH, ESCI, CCR-EXPANDED, IC Timespan=1978-2017
# 10	438,437	TS=(developing population* or developing world*) OR TS=(less developed population* or less developed world*) OR TS=(under developed population* or under developed world*) OR TS=(underdeveloped population* or underdeveloped world*) OR TS=(middle income population* or middle income world*) OR TS=(low* income population* or low* income world*)<br>Indexes=SCI-EXPANDED, SSCI, A&HCI, CPCI-S, CPCI-SSH, BKCI-S, BKCI-SSH, ESCI, CCR-EXPANDED, IC Timespan=1978-2017

For peer review only - http://bmjopen.bmj.com/site/about/guidelines.xhtml

#9	354,673	TS=(developing countr* or developing nation*) OR TS=(less developed countr* or less developed nation*) OR TS=(under developed countr* or under developed nation*) OR TS=(underdeveloped countr* or underdeveloped nation*) OR TS=(middle income countr* or middle income nation*) OR TS=(low* income countr* or low* income nation*)<br>Indexes=SCI-EXPANDED, SSCI, A&HCI, CPCI-S, CPCI-SSH, BKCI-S, BKCI-SSH, ESCI, CCR-EXPANDED, IC Timespan=1978-2017
#8	50,977	TS=(developing economies or developing economy) OR TS=(less developed economies or less developed economy) OR TS=(under developed economies or under developed economy) OR TS=(underdeveloped economies or underdeveloped economy) OR TS=(middle income economies or middle income economies) OR TS=(low* income economies or low* income economy)<br>Indexes=SCI-EXPANDED, SSCI, A&HCI, CPCI-S, CPCI-SSH, BKCI-S, BKCI-SSH, ESCI, CCR-EXPANDED, IC Timespan=1978-2017
#7	7,542	TS=(low* gdp or low* GNP or low* gross domestic or low* gross national)<br>Indexes=SCI-EXPANDED, SSCI, A&HCI, CPCI-S, CPCI-SSH, BKCI-S, BKCI-SSH, ESCI, CCR-EXPANDED, IC Timespan=1978-2017
#6	15,495	TS=(low SAME middle SAME countr*)<br>Indexes=SCI-EXPANDED, SSCI, A&HCI, CPCI-S, CPCI-SSH, BKCI-S, BKCI-SSH, ESCI, CCR-EXPANDED, IC Timespan=1978-2017
#5	32,915	TS= (lmic or lmics or third world or lami countr*)<br>Indexes=SCI-EXPANDED, SSCI, A&HCI, CPCI-S, CPCI-SSH, BKCI-S, BKCI-SSH, ESCI, CCR-EXPANDED, IC Timespan=1978-2017
#4	2,471	TS=(transitional countr*)<br>Indexes=SCI-EXPANDED, SSCI, A&HCI, CPCI-S, CPCI-SSH, BKCI-S, BKCI-SSH, ESCI, CCR-EXPANDED, IC Timespan=1978-2017
#2	522,425	TS=(Africa or Asia or Caribbean or West Indies or South America or Latin America or Central America)<br>Indexes=SCI-EXPANDED, SSCI, A&HCI, CPCI-S, CPCI-SSH, BKCI-S, BKCI-SSH, ESCI, CCR-EXPANDED, IC Timespan=1978-2017
#1	213,604	TS=Developing countries<br>Indexes=SCI-EXPANDED, SSCI, A&HCI, CPCI-S, CPCI-SSH, BKCI-S, BKCI-SSH, ESCI, CCR-EXPANDED, IC Timespan=1978-2017

For peer review only - http://bmjopen.bmj.com/site/about/guidelines.xhtml

ASSIA

ab(((community health* worker* or community health volunteer or or community health aide* or community health practitioner or community mental health* worker* or community mental health* volunteer or community mental health* aide* or village health* worker* or village health* team* or village health* guide* or lady health worker* or lady health visitor* or laywomen* or laywoman* or front-line primary healthcare or front-line primary health care or behvarz or brigadista or manzaneras or rural health assistant* or gramsakhi or lay health worker* or trained birth assistant* or accredited social health activist* or adherence support worker* or care facilitator* or (community and (treatment support*)) or (community* and (distributor* or volunteer* or care worker* or health worker)) or health extension worker* or lay counsellor* or maternal health worker* or peer educator* or shasthya shebikas or shasthya kormis or front line primary health* care worker or front line primary healthcare worker* or health activist*) AND (refresher* train* or refresher* course* or (adequa* and train*) or (continuing medical education) or (continuing medical training) or (continuing nursing education) or (continuing nursing training) or or (supervision) or (supportive supervision) or (continuing education) or ((in-service or ongoing or update or recap*) and train*)) AND (developing countries or Africa or Asia or Caribbean or West Indies or South America or Latin America or Central America or lmic or underdeveloped countries or middle income countries or low income countries or transitional countries))) OR ti((community health* worker* OR community health volunteer OR community health aide* OR community mental health* worker* OR community mental health* volunteer OR community mental health* aide* OR village health* worker* OR village health* team* OR village health* guide* OR lady health worker* OR lady health visitor* OR laywomen* OR laywoman* OR front-line primary healthcare OR front-line primary health care OR behvarz OR brigadista OR manzaneras OR rural health assistant* OR gramsakhi OR lay health worker* OR trained birth assistant* OR accredited social health activist* OR adherence support worker* OR care facilitator* OR (community AND (treatment support*)) OR (community* AND (distributor* OR volunteer* OR care worker* OR health worker)) OR health extension worker* OR lay counsellor* OR maternal health worker* OR peer educator* OR shasthy* shebikas OR shasthy* kormis OR front line primary health* care worker OR front line primary healthcare worker* OR health activist*) AND (refresher* train* OR refresher* course* OR (adequa* AND train*) OR (continuing medical education) OR (continuing medical training) OR (continuing nursing education) OR (continuing nursing training) OR (continuing education) OR ((in-service OR ongoing OR update OR recap*) AND train*)) AND (developing countries OR Africa OR Asia OR Caribbean OR West Indies OR South America OR Latin America OR Central America OR lmic OR underdeveloped countries OR middle income countries OR low income countries OR transitional countries))

For peer review only - http://bmjopen.bmj.com/site/about/guidelines.xhtml

LILACS

community health workers 7

community health volunteer 0

community health aide 2

community mental health worker 0

community mental health volunteer 0

community mental health aide or 0

village health worker 1

village health team 0

village health guide 0

lady health worker 0

lady health visitor 0

laywomen 0

laywoman 0

front-line primary healthcare 0

front-line primary health care 0

behvarz 0

brigadista 0

manzaneras or

rural health assistant* 0

gramsakhi 0

lay health worker 0

trained birth assistant 0

accredited social health activist 0

adherence support worker 0

care facilitator 0

treatment support 11

community 0

health extension worker 0

lay counsellor 0

For peer review only - http://bmjopen.bmj.com/site/about/guidelines.xhtml

maternal health worker 0

peer educator 0

shasthya shebikas 0

shasthya kormis 0

health activist 0

and

refresher training or refresher course or adequate training or adequately trained or on-going education or on-going training or continuing education or in-service training or update training or recap or supervision or supportive supervision

For peer review only

For peer review only - http://bmjopen.bmj.com/site/about/guidelines.xhtml

British Educational Index, ERIC and CINAHL via EBSCO

#	Query
S13	S10 AND S11 AND S12
S12	TI (((community N1 health* N3 (worker* or volunteer or aide* or practition*)) or (community N1 (mental health* N3 (worker* or volunteer or aide*)) or (village N1 health* N1 (worker* or team* or guide*)) or (lady health worker*) or (lady health visitor*) or (laywomen* or laywoman*) or (front-line primary healthcare) or (front-line primary health care) or behvarz or brigadista or manzaneras or (rural health assistant*) or gramsakhi or (lay health worker*) or (trained birth assistant*) or (accredited social health activist*) or (adherence support worker*) or (care facilitator*) or (community N10 (treatment support*)) or (community* N4 (distributor* or volunteer* or (care worker*) or (health worker))) or (health extension worker*) or (lay counsellor*) or (maternal health worker*) or (peer educator*) or (shasthya shebikas) or (shasthya kormis) or (front line primary health* care worker) or (front line primary healthcare worker*) or (health activist*))) OR AB (((community N1 health* N3 (worker* or volunteer or aide*)) or (community N1 (mental health* N3 (worker* or volunteer or aide*)) or (village N1 health* N1 (worker* or team* or guide*)) or (lady health worker*) or (lady health visitor*) or (laywomen* or laywoman*) or (front-line primary healthcare) or (front-line primary health care) or behvarz or brigadista or manzaneras or (rural health assistant*) or gramsakhi or (lay health worker*) or (trained birth assistant*) or (accredited social health activist*) or (adherence support worker*) or (care facilitator*) or (community N10 (treatment support*)) or (community* N4 (distributor* or volunteer* or (care worker*) or (health worker))) or (health extension worker*) or (lay counsellor*) or (maternal health worker*) or (shasthy* shebikas) or (shasthy* korbis) or (front line primary health* care worker) or (front line primary healthcare worker*) or (health activist*)))
S11	TI (((refresher* N1 (train* or course*)) or (adequa* N2 train*) or (on?going education) or (on?going training) or (continuing education) or (supervision) or (supportive supervision) or ((in-service or ongoing or update or recap*) N3 train*))) OR AB (((refresher* N1 (train* or course*)) or (adequa* N2 train*) or (on?going education) or (on?going training) or (supervision) or (supportive supervis*) or ((in-service or ongoing or update or recap*) N3 train*)))
S10	S1 OR S2 OR S3 OR S4 OR S5 OR S6 OR S7 OR S8 OR S9
S9	TI transitional countr* OR AB transitional countr*
S8	TI (lmic or lmics or third world or lami countr*)) OR AB (lmic or lmics or third world or lami countr*))
S7	TI (low N3 middle N3 countr*) OR AB (low N3 middle N3 countr*)
S6	TI ((low* N1 (gdp or gnp or gross domestic or gross national))) OR AB ((low* N1 (gdp or gnp or gross domestic or gross national)))

For peer review only - http://bmjopen.bmj.com/site/about/guidelines.xhtml

S5	TI (((developing or less* developed or under developed or underdeveloped or middle income or low* income) N1 (economy or economies))) OR AB (((developing or less* developed or under developed or underdeveloped or middle income or low* income) N1 (economy or economies)))
S4	TI (((developing or less* developed or under developed or underdeveloped or middle income or low* income or underserved or under served or deprived or poor*) N1 (countr* or nation* or population* or world))) OR AB (((developing or less* developed or under developed or underdeveloped or middle income or low* income or underserved or under served or deprived or poor*) N1 (countr* or nation* or population* or world)))
S3	TI (Afghanistan or Albania or Algeria or Angola or Antigua or Barbuda or Argentina or Armenia or Armenian or Aruba or Azerbaijan or Bahrain or Bangladesh or Barbados or Benin or Byelarus or Byelorussian or Belarus or Belorussian or Belorussia or Belize or Bhutan or Bolivia or Bosnia or Herzegovina or Hercegovina or Botswana or Brasil or Brazil or Bulgaria or Burkina Faso or Burkina Fasso or Upper Volta or Burundi or Urundi or Cambodia or Khmer Republic or Kampuchea or Cameroon or Cameroons or Cameron or Camerons or Cape Verde or Central African Republic or Chad or Chile or China or Colombia or Comoros or Comoro Islands or Comores or Mayotte or Congo or Zaire or Costa Rica or Cote d'Ivoire or Ivory Coast or Croatia or Cuba or Cyprus or Czechoslovakia or Czech Republic or Slovakia or Slovak Republic or Djibouti or French Somaliland or Dominica or Dominican Republic or East Timor or East Timur or Timor Leste or Ecuador or Egypt or United Arab Republic or El Salvador or Eritrea or Estonia or Ethiopia or Fiji or Gabon or Gabonese Republic or Gambia or Gaza or Georgia Republic or Georgian Republic or Ghana or Gold Coast or Greece or Grenada or Guatemala or Guinea or Guam or Guiana or Guyana or Haiti or Honduras or Hungary or India or Maldives or Indonesia or Iran or Iraq or Isle of Man or Jamaica or Jordan or Kazakhstan or Kazakh or Kenya or Kiribati or Korea or Kosovo or Kyrgyzstan or Kirghizia or Kyrgyz Republic or Kirghiz or Kirgizstan or Lao PDR or Laos or Latvia or Lebanon or Lesotho or Basutoland or Liberia or Libya or Lithuania or Macedonia or Madagascar or Malagasy Republic or Malaysia or Malaya or Malay or Sabah or Sarawak or Malawi or Nyasaland or Mali or Malta or Marshall Islands or Mauritania or Mauritius or Agalega Islands or Mexico or Micronesia or Middle East or Moldova or Moldovia or Moldovian or Mongolia or Montenegro or Morocco or Ifni or Mozambique or Myanmar or Myanma or Burma or Namibia or Nepal or Netherlands Antilles or New Caledonia or Nicaragua or Niger or Nigeria or Northern Mariana Islands or Oman or Muscat or Pakistan or Palau or Palestine or Panama or Paraguay or Peru or Philippines or Philipines or Phillipines or Phillippines or Poland or Portugal or Puerto Rico or Romania or Rumania or Roumania or Russia or Russian or Rwanda or Ruanda or Saint Kitts or St Kitts or Nevis or Saint Lucia or St Lucia or Saint Vincent or St Vincent or Grenadines or Samoa or Samoan Islands or Navigator Island or Navigator Islands or Sao Tome or Saudi Arabia or Senegal or Serbia or Montenegro or Seychelles or Sierra Leone or Slovenia or Sri Lanka or Ceylon or Solomon Islands or Somalia or South Africa or Sudan or Suriname or Surinam or Swaziland or Syria or Tajikistan or Tadzhikistan or Tadjikistan or Tadzhik or Tanzania or Thailand or Togo or Togolese Republic or Tonga or Trinidad or

For peer review only - http://bmjopen.bmj.com/site/about/guidelines.xhtml

Tobago or Tunisia or Turkey or Turkmenistan or Turkmen or Uganda or Ukraine or Uruguay or USSR or Soviet Union or Union of Soviet Socialist Republics or Uzbekistan or Uzbek or Vanuatu or New Hebrides or Venezuela or Vietnam or Viet Nam or West Bank or Yemen or Yugoslavia or Zambia or Zimbabwe or Rhodesia) OR AB (Afghanistan or Albania or Algeria or Angola or Antigua or Barbuda or Argentina or Armenia or Armenian or Aruba or Azerbaijan or Bahrain or Bangladesh or Barbados or Benin or Byelarus or Byelorussian or Belarus or Belorussian or Belorussia or Belize or Bhutan or Bolivia or Bosnia or Herzegovina or Hercegovina or Botswana or Brasil or Brazil or Bulgaria or Burkina Faso or Burkina Fasso or Upper Volta or Burundi or Urundi or Cambodia or Khmer Republic or Kampuchea or Cameroon or Cameroons or Cameron or Camerons or Cape Verde or Central African Republic or Chad or Chile or China or Colombia or Comoros or Comoro Islands or Comores or Mayotte or Congo or Zaire or Costa Rica or Cote d'Ivoire or Ivory Coast or Croatia or Cuba or Cyprus or Czechoslovakia or Czech Republic or Slovakia or Slovak Republic or Djibouti or French Somaliland or Dominica or Dominican Republic or East Timor or East Timur or Timor Leste or Ecuador or Egypt or United Arab Republic or El Salvador or Eritrea or Estonia or Ethiopia or Fiji or Gabon or Gabonese Republic or Gambia or Gaza or Georgia Republic or Georgian Republic or Ghana or Gold Coast or Greece or Grenada or Guatemala or Guinea or Guam or Guiana or Guyana or Haiti or Honduras or Hungary or India or Maldives or Indonesia or Iran or Iraq or Isle of Man or Jamaica or Jordan or Kazakhstan or Kazakh or Kenya or Kiribati or Korea or Kosovo or Kyrgyzstan or Kirghizia or Kyrgyz Republic or Kirghiz or Kirgizstan or Lao PDR or Laos or Latvia or Lebanon or Lesotho or Basutoland or Liberia or Libya or Lithuania or Macedonia or Madagascar or Malagasy Republic or Malaysia or Malaya or Malay or Sabah or Sarawak or Malawi or Nyasaland or Mali or Malta or Marshall Islands or Mauritania or Mauritius or Agalega Islands or Mexico or Micronesia or Middle East or Moldova or Moldovia or Moldovian or Mongolia or Montenegro or Morocco or Ifni or Mozambique or Myanmar or Myanma or Burma or Namibia or Nepal or Netherlands Antilles or New Caledonia or Nicaragua or Niger or Nigeria or Northern Mariana Islands or Oman or Muscat or Pakistan or Palau or Palestine or Panama or Paraguay or Peru or Philippines or Philipines or Phillipines or Phillippines or Poland or Portugal or Puerto Rico or Romania or Rumania or Roumania or Russia or Russian or Rwanda or Ruanda or Saint Kitts or St Kitts or Nevis or Saint Lucia or St Lucia or Saint Vincent or St Vincent or Grenadines or Samoa or Samoan Islands or Navigator Island or Navigator Islands or Sao Tome or Saudi Arabia or Senegal or Serbia or Montenegro or Seychelles or Sierra Leone or Slovenia or Sri Lanka or Ceylon or Solomon Islands or Somalia or South Africa or Sudan or Suriname or Surinam or Swaziland or Syria or Tajikistan or Tadzhikistan or Tadjikistan or Tadzhik or Tanzania or Thailand or Togo or Togolese Republic or Tonga or Trinidad or Tobago or Tunisia or Turkey or Turkmenistan or Turkmen or Uganda or Ukraine or Uruguay or USSR or Soviet Union or Union of Soviet Socialist Republics or Uzbekistan or Uzbek or Vanuatu or New Hebrides or Venezuela or Vietnam or Viet Nam or West Bank or Yemen or Yugoslavia or Zambia or Zimbabwe or Rhodesia)

S2 SU (Africa or Asia or Caribbean or West Indies or South America or Latin America or Central America) OR MW (Africa or Asia or Caribbean or West

For peer review only - http://bmjopen.bmj.com/site/about/guidelines.xhtml

	Indies or South America or Latin America or Central America) OR MM (Africa or Asia or Caribbean or West Indies or South America or Latin America or Central America) OR MJ (Africa or Asia or Caribbean or West Indies or South America or Latin America or Central America) OR TI (Africa or Asia or Caribbean or West Indies or South America or Latin America or Central America) OR AB (Africa or Asia or Caribbean or West Indies or South America or Latin America or Central America)
S1	SU Developing Countries OR MW Developing Countries

For peer review only

For peer review only - http://bmjopen.bmj.com/site/about/guidelines.xhtml

Supplementary Table 1. Community Health Worker Terminology. Nomenclature used to describe Community Health Workers' by study.

**Term used to describe 'Community Health Worker'**	**Number of studies**
Community Health Worker (CHW)[1-10]	10
Health Surveillance Assistant (HSA)[11-13]	3
Community Health Volunteer (CHV)[14-16]	3
Lay counsellors[17-19]	3
Health Extension Worker (HEW)[20-22]	3
Lady Health Worker (LHW)[23,24]	2
Community Based Physician Associate (CBPA)[25,26]	2
Community Health Agent (CHA)[27]	1
Maternal and Child Health Worker[28]	1
Behvarz[29]	1
Agentes Polivalentes Elementares (APEs)[30]	1
Community Health Guide/Aragandi Worker[31]	1
Acredited Social Health Activist (ASHA)[32]	1
Community Workers (CWs)[33]	1
Community Health Promoter (CHP)[34]	1
Community Volunteers (CVs)[35]	1
Health Workers (HWs)[36]	1

For peer review only - http://bmjopen.bmj.com/site/about/guidelines.xhtml

Authors	Study Title	Year Published	Country and Region	Total number of CHWs involved	Disease Focus Area	Type of on-going training and total number of sessions provided	Outcome measure
Adejumo et al.[33]	Community referral for presumptive TB in Nigeria: a comparison of four models of active case finding.	2016	Nigeria, West Africa	124	TB	**Supervision.** Variable number of supervisory visits, ranging from monthly to three monthly.	**Change in practice or behaviour** e.g. number of cases of TB detected in the community.
Ameha et al.[20]	Effectiveness of supportive supervision on the consistency of integrated community cases management skills of the health extension workers in 113 districts of Ethiopia.	2013	Ethiopia, East Africa	5000	Child Health	**Supervision.** Variable number of supervisory visits. *(minimum of one, maximum of four)*	**Change in practice or behaviour** e.g. number of recorded cases of diahorrea, malaria and pneumonia managed correctly in the community.
Ayele et al.[27]	The functional status of community health agents: A trial of refresher courses and regular supervision.	1993	Ethiopia, East Africa	102	General focus	**Refresher training course and supervision.** Five-day refresher course and one supervision per month.	**Change in practice or behaviour** e.g. number of home visits, registration activities
Carlough & McCall[28]	Skilled birth attendance: What does it mean and how can it be measured? A	2005	Nepal, South Asia	104 (66 received refresher	Maternal and Reproductive Health	**Refresher training course.** Six-week refresher training	**Mixed methods.** Knowledge and skills assessment using a

	clinical skills assessment of maternal and child health workers in Nepal.			training)		course.	clinical skills assessment tool, plus a qualitative self-assessment scale.
Das et al.[32]	Strengthening malaria service delivery through supportive supervision and community mobilization in an endemic Indian setting: an evaluation of nested delivery models	2015	India, South Asia	N/A (randomised at village level)	Infectious disease	**Supervision.** Twice monthly.	**Change in practice or behaviour** e.g. Assessing for increased use of long last insecticide treated bed nets and proportion of cases tested for falciparum malaria within 24 hours.
Datiko et al.[34]	Exploring providers' perspectives of a community based TB approach in Southern Ethiopia: implication for community based approaches.	2015	Ethiopia, East Africa	20	TB	**Supervision.** Twice monthly.	**Qualitative assessment.** Interviews and focus discussion groups to elicit the experiences of providers.
Dewing et al.[19]	Lay Counselors' Ability to Deliver Counseling for Behavior Change.	2013	South Africa, Southern Africa	39	HIV	**Refresher training course.** 18 hours of refresher training over a 12-month period.	**Knowledge and skills assessment.** Lay counselors ability in motivational interviewing was assessed following refresher training using the Motivational Interviewing Treatment Integrity

For peer review only - http://bmjopen.bmj.com/site/about/guidelines.xhtml

							Tool and an instrument developed by the researchers.
Gallo et al.[1]	Evaluation of a volunteer community-based health worker program for providing contraceptive services in Madagascar.	2013	Madagascar, East Africa	100	Maternal and Reproductive Health	**Refresher training course.** Two-day refresher training course for those who did not meet the minimum level of competency following initial training.	**Knowledge and skills assessment.** A test involving five stimulated encounters regarding knowledge of an injectable contraception.
*Gill et al.[25]	The mCME Project: A Randomized controlled trial of an SMS-Based continuing medical education intervention for improving medical knowledge among Vietnamese community based physicians' assistants.	2016	Vietnam, South-East Asia	593	General focus	**In-service training.** Once daily SMS messages.	**Mixed methods.** Knowledge and skills assessment using a 100-item test, plus a qualitative self-evaluation and qualitative job satisfaction assessment.
Gupta et al.[31]	Implementation of ORT: some problems encountered in training of health workers during an operational research programme.	1994	India, South Asia	323	Child Health	**Refresher training course.** One-day interactive refresher training course.	**Knowledge and skills assessment.** Testing for improved knowledge of ORS using role playing and discussions.
Gupta et al.[36]	Improving quality of home-based postnatal care by microteaching of multipurpose workers in	2016	India, South Asia	12	Child Health	**In-service training.** One 90-minute session every three months.	**Knowledge and skills assessment.** Scores achieved on a structured checklist

For peer review only - http://bmjopen.bmj.com/site/about/guidelines.xhtml

	rural and urban slum areas of Chandigarh, India: a pilot study.						with items regarding maternal history and exam taking technique, new-born examination and maternal counselling.
Hadi.[16]	Management of acute repiratory infections by community health volunteers: experience of Bangladesh Rural Advancement Committee (BRAC).	2003	Bangladesh, South Asia	120	Child Health	**Supervision.** Once a month.	**Knowledge and skills assessment.** Comparing the diagnosis of acute respiratory infection and management between CHWs and trained assessors.
Horwood et al.[7]	A continuous quality improvement intervention to improve the effectiveness of community health workers providing care to mothers and children: a cluster randomised controlled trial in South Africa	2017	South Africa, Southern Africa	120	Maternal and Child Health.	**Supervision.** Twice monthly.	**Mixed methods.** Knowledge and skills were assessed using four questions, which were asked to mothers served by the CHWs regarding antenatal care. Markers including the number of household visits performed by the CHW assessed behaviour change.
Javanparast et al.[29]	The experience of community health workers training in Iran: a qualitative study.	2012	Iran, Middle East	91	General focus	**In-service training.** Variable - ranging from monthly to bi-annually.	**Qualitative evaluation** e.g. interviews with behvarz.
Joos et al.[13]	Evaluation of a mHealth	2016	Malawi,	160	Maternal Health	**Supervision.**	**Change in practice**

For peer review only - http://bmjopen.bmj.com/site/about/guidelines.xhtml

	data Quality Intervention to Improve Documentation of Pregnancy Outcomes by Health Surveillance Assistants in Malawi: A cluster RCT.		East Africa			2-5 informative or motivational SMS messages sent each week.	**or behaviour** e.g. Improved recording of pregnancy.
Kawasaki et al.[2]	Reactions of community members regarding community health workers' activities as a measure of the impact of a training program in Amazonas, Brazil.	2015	Brazil, South America	102	General focus	**Refresher training course.** Once a month refresher training sessions.	**Qualitative assessment.** Interviews with key stakeholders on levels of satisfaction.
Kuule et al.[14]	Community Health Volunteers in Primary Healthcare in Rural Uganda: Factors Influencing Performance.	2017	Uganda, East Africa	508	Child & Maternal and Reproductive Health	**Refresher training course and supervision.** Biannual refresher training sessions and monthly supervisions.	**Change in practice or behaviour** e.g. attendance at meetings, household follow-up and reporting, immunization coverage.
Mash et al.[17]	Reflections on the training of counsellors in motivational interviewing for programmes for the prevention of mother to child transmission of HIV in sub-Saharan Africa.	2008	South Africa, Southern Africa	18	HIV	**Supervision.** Once monthly.	**Knowledge and skills assessment.** Assessment of motivational interview techniques.
McLean et al.[8]	Task sharing in rural Haiti: Qualitative assessment of a brief, structured training with and without	2015	Haiti, Central America	3	Mental Health	**Supervision.** After initial training one week of daily observation by a	**Mixed methods.** Change in practice and behaviour measured through

For peer review only - http://bmjopen.bmj.com/site/about/guidelines.xhtml

	apprenticeship supervision for community health workers.					licensed counselor followed by one further week of supervised sessions.	home visits, provision of supportive visits and referrals. Qualitative assessment of confidence and satisfaction.
Mengistu et al.[21]	Effect of performance review and clinical mentoring meetings (PRCMM) on recording of community case management by health extension workers in Ethiopia.	2014	Ethiopia, East Africa	1175	Child health	**Supervision.** Twice monthly.	**Change in practice or behaviour** e.g. Improved recording and adherence to iCCM guidelines.
Miller et al.[22]	Assessment of the impact of quality improvement interventions on the quality of sick child-care provided by Health Extension Workers in Ethiopia.	2016	Ethiopia, East Africa	157 (based on an estimate of 1.5 HEWs across 104 health posts)	Child health	**Refresher training course and supervision.** One day refresher training course eight weeks after intial iCCM training followed by quarterly supervision sessions.	**Change in practice and behaviour** e.g. number of children correctly managed according to iCCM guidelines.
Mkumbo et al.[9]	Innovation in supervision and support of community health workers for better newborn survival in southern Tanzania.	2014	Tanzania, East Africa	824	Child health	**Supervision.** Quarterly meetings.	**Change in practice and behaviour** e.g. number of volunteer–supervisor contacts
Msisuka et	An evaluation of a	2011	Zambia,	25	HIV	**Refresher training**	**Mixed methods.**

For peer review only - http://bmjopen.bmj.com/site/about/guidelines.xhtml

al.[18]	refresher training intervention for HIV lay counsellors in Chongwe District, Zambia.		East Africa			**course.** Two-day refresher training course.	Knowledge and skills assessment was answering true or false questions on a 25-question quiz and testing 10 blood panel samples. Qualitative assessment regarding motivations and obstacles to performance.
Ndima et al.[30]	Supervision of community health workers in Mozambique: a qualitative study of factors influencing motivation and programme implementation.	2015	Mozambiqu e, East Africa	18	Child & Maternal and Reproductive Health	**Supervision.** Monthly supervisions at the community health centre and quarterly supervisions in the community.	**Mixed methods.** Qualitative assessment e.g. interviews regarding motivation and change in practice e.g. number of home visits and referrals.
**Puchalski Ritchie et al.[12]	A knowledge translation intervention to improve tuberculosis care and outcomes in Malawi: a pragmatic cluster randomized controlled trial.	2015	Malawi, East Africa	36	TB/HIV	**In-service training.** Six on-going training courses lasting for 60-90 minutes over three-months.	**Qualitative assessment.** Interviews with CHWs regarding perceived improvement in knowledge and skills and ability to perform their roles.
**Puchalski Ritchie et al.[11]	Lay Health Workers experience of a tailored knowledge translation intervention to improve	2016	Malawi, East Africa	49	TB/HIV	**In-service training.** Six on-going training courses lasting for 60-90	**Change in practice or behaviour.** Measured through assessing adherence

For peer review only - http://bmjopen.bmj.com/site/about/guidelines.xhtml

	job skills and knowledge: a qualitative study in Zomba district Malawi.					minutes over three-months.	to TB medications and improvements in clinical conditions at the community level.
Rabbani et al.[23]	Health workers' perspectives, knowledge and skills regarding community care management of childhood diahorrea and pneumonia: a qualitative inquiry for an implementation research project "Nigraan" in District Badin, Sindh, Pakistan.	2016	Pakistan, South Asia	108	Child Health	**Supervision.** Twice monthly.	**Mixed methods.** Knowledge and skills assessment regarding management plus qualitative perceptions of the supervision.
Rabbani et al.[24]	Inspiring health worker motivation with supportive supervision: a survey of lady health supervisor motivating factors in rural Pakistan.	2016	Pakistan, South Asia	29	Child Health	**Supervision.** Twice monthly.	**Qualitative measures** e.g. motivation following supervision.
Robertson et al.[4]	Initial experiences and innovations in supervising community health workers for maternal, newborn, and childhealth in Morogoro region, Tanzania.	2015	Tanzania, East Africa	228	Maternal and Child Health	**Supervision.** Once monthly.	**Mixed methods.** Qualitative interviews assessing CHWs experiences and assessment of knowledge and skills through a survey.
Rowe et al.[3]	Longitudinal analysis of community health workers' adherence to treatment	2007	Kenya, East Africa	114	Child Health	**Refresher training course.** Two three-month blocks of	**Change in practice or behaviour** e.g. correct referrals and

For peer review only - http://bmjopen.bmj.com/site/about/guidelines.xhtml

	guidelines, Siaya, Kenya, 1997-2002.					refresher training.	management of sick children.
*Sabin et al.[26]	Benefits and Limitations of TextMessages to Stimulate Higher Learning Among Community Providers: Participants' Views of an mHealth Intervention to Support Continuing Medical Education in Vietnam.	2017	Vietnam, South East Asia	70	General focus	**In-service training.** Once daily SMS messages.	**Qualitative analysis.** Aimed to elict the views and experiances of CBPAs involved in the mCME study.
Singh et al.[15]	Supportive supervision for volunteers to deliver reproductive health education: a cluster randomized trial.	2016	Uganda, East Africa	82	General focus	**Supervision.** Monthly training lasting for between two to three hours per month.	**Change in practice or behaviour** e.g. immunizations, breastfeeding, number of installed tippy taps for hand washing assessed at the household level.
Talukder et al.[35]	In a rural area of Bangladesh, traditional birth attendant training improved early infant feeding practices: a pragmatic cluster randomized trial.	2016	Bangladesh, South Asia	N/A – randomisation done at district level	Child health	**Supervision.** Once a week supervision sessions.	**Change in behaviour or practice** e.g. number of home visits, initiation of breastfeeding.
Vu Henry et al.[5]	Enhancing the Supervision of Community Health Workers With WhatsApp Mobile Messaging: Qualitative Findings From	2016	Kenya, East Africa	25	General focus	**Supervision.** Continuous supportive supervision over a period of six months	**Qualitative analysis** of WhatsApp messages.

For peer review only - http://bmjopen.bmj.com/site/about/guidelines.xhtml

	2 Low-Resource Settings in Kenya.					via WhatsApp.	
Vallières et al.[10]	There's No App for That: Assessing the Impact of mHealth on the Supervision, Motivation, Engagement, and Satisfaction of Community Health Workers in Sierra Leone.	2016	Sierra Leone, West Africa	293	Maternal and child health	**Supervision.** Through a communication group between CHWs and their supervisors installed on a mobile phone.	**Qualitative assessment.** Self reported measures of work engagement and job satisfaction.
Zeitz et al.[6]	Community health worker competency in managing acute respiratory infections of childhood in Bolivia.	1993	Bolivia, South America	80	Child Health	**Refresher training course.** One-day refresher course lasting for eight hours.	**Knowledge and skills assessment** using a pre- and post-intervention test.

Supplementary Table 2. Details of included studies. Legend: A breakdown of individual study details arranged in first author alphabetical order.

Key:

*Two separate analyses from the same study. One study focused on a knowledge and skills assessment of a mobile-based continuing medical education initiative using a 100-item test, plus a qualitative self-evaluation and job satisfaction assessment,[25] the other focused on an in-depth qualitative evaluation of the same study.[26]

**Two separate analyses from the same study. One study focused on the number of HIV and TB cases correctly managed following a knowledge translation intervention,[12] the other focused on a qualitative evaluation of the same intervention.[11]

For peer review only - http://bmjopen.bmj.com/site/about/guidelines.xhtml

Supplementary material references:

1. Gallo MF, Walldorf J, Kolesar R, et al. Evaluation of a volunteer community-based health worker program for providing contraceptive services in Madagascar. *Contraception.* 2013;88(5):657-665.
2. Kawasaki R, Sadamori T, Ferreira de Almeida T, et al. Reactions of community members regarding community health workers' activities as a measure of the impact of a training program in Amazonas, Brazil. *Journal of Rural Medicine.* 2015;10(1):7-19.
3. Rowe SY, Olewe MA, Kleinbaum DG, et al. Longitudinal analysis of community health workers' adherence to treatment guidelines, Siaya, Kenya, 1997-2002. *Tropical Medicine and International Health.* 2007;12(5):651-663.
4. Roberton T, Applegate J, Lefevre AE, et al. Initial experiences and innovations in supervising community health workers for maternal, newborn, and child health in Morogoro region, Tanzania. *Hum Resour Health.* 2015;13:19.
5. Henry JV, Winters N, Lakati A, et al. Enhancing the Supervision of Community Health Workers With WhatsApp Mobile Messaging: Qualitative Findings From 2 Low-Resource Settings in Kenya. *Glob Health Sci Pract.* 2016;4(2):311-325.
6. Zeitz PS, Harrison LH, Lopez M, Cornale G. Community health worker competency in managing acute respiratory infections of childhood in Bolivia. *Bull Pan Am Health Organ.* 1993;27(2):109-119.
7. Horwood C, Butler L, Barker P, et al. A continuous quality improvement intervention to improve the effectiveness of community health workers providing care to mothers and children: a cluster randomised controlled trial in South Africa. *Hum Resour Health.* 2017;15(1):39.
8. McLean KE, Kaiser BN, Hagaman AK, Wagenaar BH, Therosme TP, Kohrt BA. Task sharing in rural Haiti: Qualitative assessment of a brief, structured training with and without apprenticeship supervision for community health workers. *Intervention (Amstelveen).* 2015;13(2):135-155.
9. Mkumbo E, Hanson C, Penfold S, Manzi F, Schellenberg J. Innovation in supervision and support of community health workers for better newborn survival in southern Tanzania. *Int Health.* 2014;6(4):339-341.
10. Vallieres F, McAuliffe E, van Bavel B, Wall PJ, Trye A. There's No App for That: Assessing the Impact of mHealth on the Supervision, Motivation, Engagement, and Satisfaction of Community Health Workers in Sierra Leone. *Ann Glob Health.* 2016;82(5):936-949.
11. Puchalski Ritchie LM, van Lettow M, Barnsley J, et al. Lay Health Workers experience of a tailored knowledge translation intervention to improve job skills and knowledge: a qualitative study in Zomba district Malawi. *BMC Medical Education.* 2016;16:54.
12. Puchalski Ritchie LM, Schull MJ, Martiniuk AL, et al. A knowledge translation intervention to improve tuberculosis care and outcomes in Malawi: a pragmatic cluster randomized controlled trial. *Implement Sci.* 2015;10:38.
13. Joos O, Silva R, Amouzou A, et al. Evaluation of a mHealth Data Quality Intervention to Improve Documentation of Pregnancy Outcomes by Health Surveillance Assistants in Malawi: A Cluster Randomized Trial. *PLoS One.* 2016;11(1):e0145238.
14. Kuule Y, Dobson AE, Woldeyohannes D, et al. Community Health Volunteers in Primary Healthcare in Rural Uganda: Factors Influencing Performance. *Frontiers in Public Health.* 2017;5:62.
15. Singh D, Negin J, Orach CG, Cumming R. Supportive supervision for volunteers to deliver reproductive health education: a cluster randomized trial. *Reproductive Health.* 2016;13(1):126.

For peer review only - http://bmjopen.bmj.com/site/about/guidelines.xhtml

16. Hadi A. Management of acute respiratory infections by community health volunteers: experience of Bangladesh Rural Advancement Committee (BRAC). *Bulletin of the World Health Organization.* 2003;81(3):183-189.
17. Mash R, Baldassini G, Mkhatshwa H, Sayeed I, Ndapeua S, Mash B. Reflections on the training of counsellors in motivational interviewing for programmes for the prevention of mother to child transmission of HIV in sub-Saharan Africa. *South African Family Practice.* 2008;50(2):53-59.
18. Msisuka C, Nozaki I, Kakimoto K, Seko M, Ulaya MM. An evaluation of a refresher training intervention for HIV lay counsellors in Chongwe District, Zambia. *SAHARA J: Journal of Social Aspects of HIV/AIDS Research Alliance.* 2011;8(4):204-209.
19. Dewing S, Mathews C, Cloete A, Schaay N, Simbayi L, Louw J. Lay counselors' ability to deliver counseling for behavior change. *J Consult Clin Psychol.* 2014;82(1):19-29.
20. Ameha A, Karim AM, Erbo A, et al. Effectiveness of supportive supervision on the consistency of integrated community cases management skills of the health extension workers in 113 districts of Ethiopia. *Ethiopian Medical Journal.* 2014;52 Suppl 3:65-71.
21. Mengistu B, Karim AM, Eniyew A, et al. Effect of performance review and clinical mentoring meetings (PRCMM) on recording of community case management by health extension workers in Ethiopia. *Ethiop Med J.* 2014;52 Suppl 3:73-81.
22. Miller NP, Amouzou A, Hazel E, et al. Assessment of the impact of quality improvement interventions on the quality of sick child care provided by Health Extension Workers in Ethiopia. *J Glob Health.* 2016;6(2):020404.
23. Rabbani F, Perveen S, Aftab W, Zahidie A, Sangrasi K, Qazi SA. Health workers' perspectives, knowledge and skills regarding community case management of childhood diarrhoea and pneumonia: a qualitative inquiry for an implementation research project "Nigraan" in District Badin, Sindh, Pakistan. *BMC Health Serv Res.* 2016;16:462.
24. Rabbani F, Shipton L, Aftab W, Sangrasi K, Perveen S, Zahidie A. Inspiring health worker motivation with supportive supervision: a survey of lady health supervisor motivating factors in rural Pakistan. *BMC Health Serv Res.* 2016;16(1):397.
25. Gill CJ, Ngoc BL, Halim N, et al. The mCME Project: A Randomized controlled trial of an SMS-Based continuing medical education intervention for improving medical knowledge among Vietnamese community based physicians' assistants. *PLoS ONE.* 2016;11 (11) (no pagination)(e0166293).
26. Sabin LL, Larson Williams A, Le BN, et al. Benefits and Limitations of Text Messages to Stimulate Higher Learning Among Community Providers: Participants' Views of an mHealth Intervention to Support Continuing Medical Education in Vietnam. *Glob Health Sci Pract.* 2017;5(2):261-273.
27. Ayele F, Desta A, Larson C. The functional status of community health agents: A trial of refresher courses and regular supervision. *Health Policy and Planning.* 1993;8(4):379-384.
28. Carlough M, McCall M. Skilled birth attendance: What does it mean and how can it be measured? A clinical skills assessment of maternal and child health workers in Nepal. *International Journal of Gynecology & Obstetrics.* 2005;89(2):200-208.
29. Javanparast S, Baum F, Labonte R, Sanders D, Rajabi Z, Heidari G. The experience of community health workers training in Iran: a qualitative study. *BMC Health Services Research.* 2012;12:291.
30. Ndima SD, Sidat M, Give C, Ormel H, Kok MC, Taegtmeyer M. Supervision of community health workers in Mozambique: a qualitative study of factors influencing motivation and programme implementation. *Human Resources for Health [Electronic Resource].* 2015;13:63.
31. Gupta DN, SenGupta PG, Sircar BK, Mondal S, Sarkar S, Deb BC. Implementation of ORT: some problems encountered in training of health workers during an operational research programme. *Indian Journal of Public Health.* 1994;38(2):69-72.

For peer review only - http://bmjopen.bmj.com/site/about/guidelines.xhtml

32. Das A, Friedman J, Kandpal E, et al. Strengthening malaria service delivery through supportive supervision and community mobilization in an endemic Indian setting: an evaluation of nested delivery models. *Malar J.* 2014;13:482.
33. Adejumo AO, Azuogu B, Okorie O, et al. Community referral for presumptive TB in Nigeria: a comparison of four models of active case finding. *BMC Public Health.* 2016;16:177.
34. Datiko DG, Yassin MA, Tulloch O, et al. Exploring providers' perspectives of a community based TB approach in Southern Ethiopia: implication for community based approaches. *BMC Health Serv Res.* 2015;15:501.
35. Talukder S, Farhana D, Vitta B, Greiner T. In a rural area of Bangladesh, traditional birth attendant training improved early infant feeding practices: a pragmatic cluster randomized trial. *Matern Child Nutr.* 2017;13(1).
36. Gupta M, Tripathy JP, Jamir L, Sarwa A, Sinha S, Bhag C. Improving quality of home-based postnatal care by microteaching of multipurpose workers in rural and urban slum areas of Chandigarh, India: a pilot study. *Advances in Medical Education and Practice.* 2017;8:1-8.

For peer review only

PRISMA 2009 Checklist

Section/topic	#	Checklist item	Reported on page #
**TITLE**			
Title	1	Identify the report as a systematic review, meta-analysis, or both.	1,2 and 8
**ABSTRACT**			
Structured summary	2	Provide a structured summary including, as applicable: background; objectives; data sources; study eligibility criteria, participants, and interventions; study appraisal and synthesis methods; results; limitations; conclusions and implications of key findings; systematic review registration number.	2
**INTRODUCTION**			
Rationale	3	Describe the rationale for the review in the context of what is already known.	5-7
Objectives	4	Provide an explicit statement of questions being addressed with reference to participants, interventions, comparisons, outcomes, and study design (PICOS).	6-7 & 11-12
**METHODS**			
Protocol and registration	5	Indicate if a review protocol exists, if and where it can be accessed (e.g., Web address), and, if available, provide registration information including registration number.	NA (See reason on page 8)
Eligibility criteria	6	Specify study characteristics (e.g., PICOS, length of follow-up) and report characteristics (e.g., years considered, language, publication status) used as criteria for eligibility, giving rationale.	10-11
Information sources	7	Describe all information sources (e.g., databases with dates of coverage, contact with study authors to identify additional studies) in the search and date last searched.	9-10
Search	8	Present full electronic search strategy for at least one database, including any limits used, such that it could be repeated.	Supplementary material 1-16
Study selection	9	State the process for selecting studies (i.e., screening, eligibility, included in systematic review, and, if applicable, included in the meta-analysis).	10-11
Data collection process	10	Describe method of data extraction from reports (e.g., piloted forms, independently, in duplicate) and any processes for obtaining and confirming data from investigators.	13
Data items	11	List and define all variables for which data were sought (e.g., PICOS, funding sources) and any assumptions and simplifications made.	11&12
Risk of bias in individual studies	12	Describe methods used for assessing risk of bias of individual studies (including specification of whether this was done at the study or outcome level), and how this information is to be used in any data synthesis.	N/A (see comment in strengths and limitations

For peer review only

PRISMA 2009 Checklist

			section on page 3)
Summary measures	13	State the principal summary measures (e.g., risk ratio, difference in means).	N/A
Synthesis of results	14	Describe the methods of handling data and combining results of studies, if done, including measures of consistency (e.g., I^2) for each meta-analysis.	N/A

Section/topic	#	Checklist item	Reported on page #
Risk of bias across studies	15	Specify any assessment of risk of bias that may affect the cumulative evidence (e.g., publication bias, selective reporting within studies).	N/A (see comment in strengths and limitations section on page 3)
Additional analyses	16	Describe methods of additional analyses (e.g., sensitivity or subgroup analyses, meta-regression), if done, indicating which were pre-specified.	N/A
**RESULTS**			
Study selection	17	Give numbers of studies screened, assessed for eligibility, and included in the review, with reasons for exclusions at each stage, ideally with a flow diagram.	14&16
Study characteristics	18	For each study, present characteristics for which data were extracted (e.g., study size, PICOS, follow-up period) and provide the citations.	Supplementary material
Risk of bias within studies	19	Present data on risk of bias of each study and, if available, any outcome level assessment (see item 12).	N/A (see comment in strengths and limitations section on page 3)
Results of individual studies	20	For all outcomes considered (benefits or harms), present, for each study: (a) simple summary data for each intervention group (b) effect estimates and confidence intervals, ideally with a forest plot.	Supplementary material
Synthesis of results	21	Present results of each meta-analysis done, including confidence intervals and measures of consistency.	N/A
Risk of bias across studies	22	Present results of any assessment of risk of bias across studies (see Item 15).	N/A
Additional analysis	23	Give results of additional analyses, if done (e.g., sensitivity or subgroup analyses, meta-regression [see Item	N/A

For peer review only

For peer review only - http://bmjopen.bmj.com/site/about/guidelines.xhtml

PRISMA 2009 Checklist

		16]).	
**DISCUSSION**			
Summary of evidence	24	Summarize the main findings including the strength of evidence for each main outcome; consider their relevance to key groups (e.g., healthcare providers, users, and policy makers).	Supplementary material
Limitations	25	Discuss limitations at study and outcome level (e.g., risk of bias), and at review-level (e.g., incomplete retrieval of identified research, reporting bias).	23
Conclusions	26	Provide a general interpretation of the results in the context of other evidence, and implications for future research.	24
**FUNDING**			
Funding	27	Describe sources of funding for the systematic review and other support (e.g., supply of data); role of funders for the systematic review.	3

From: Moher D, Liberati A, Tetzlaff J, Altman DG, The PRISMA Group (2009). Preferred Reporting Items for Systematic Reviews and Meta-Analyses: The PRISMA Statement. PLoS Med 6(7): e1000097. doi:10.1371/journal.pmed1000097

For peer review only

BMJ Open

The on-going training of Community Health Workers in low- and middle-income countries: A systematic scoping review of the literature.

Journal:	*BMJ Open*
Manuscript ID	bmjopen-2017-021467.R1
Article Type:	Research
Date Submitted by the Author:	06-Mar-2018
Complete List of Authors:	O'Donovan, James; University of Oxford , Department of Education<br>O'Donovan, Charles; University of Leeds Faculty of Medicine and Health<br>Kuhn, Isla; University of Cambridge, Medical Library, School of Clinical Medicine<br>Sachs, Sonia; Earth Institute at Columbia University<br>Winters, Niall; University of Oxford, Education
**Primary Subject Heading**:	Global health
Secondary Subject Heading:	Medical education and training, Public health, Health services research, Health policy
Keywords:	MEDICAL EDUCATION & TRAINING, International health services < HEALTH SERVICES ADMINISTRATION & MANAGEMENT, Community Health Worker, Lay health worker, Supervision, on-going training

SCHOLARONE™ Manuscripts

only

Full Title: The on-going training of Community Health Workers in low- and middle-income countries: A systematic scoping review of the literature.

Authors

James O'Donovan[1*], Charles O'Donovan[2], Isla Kuhn[3], Sonia Sachs[4], Niall Winters[1].

Affiliations

1. University of Oxford, Department of Education, Oxford, Oxfordshire, UK.
2. University of Leeds Faculty of Medicine and Health, Leeds, West Yorkshire, UK.
3. University of Cambridge, Medical Library, School of Clinical Medicine, Cambridge, UK.
4. Earth Institute at Columbia University, New York, NY USA.

*Corresponding Author

Address: University of Oxford, Department of Education, Norham Gardens, Oxford, OX2 6PY.

Telephone: +44 7999177071

Email: james.odonovan@seh.ox.ac.uk

Word Count (excluding title page, abstract, references, figures and tables)**:** 4951

For peer review only

Abstract

Objectives: Understanding the current landscape of on-going training for Community Health Workers (CHWs) in low- and middle-income countries (LMICs) is important both for organisations responsible for their training, as well as researchers and policy makers. This scoping review explores this under-researched area by mapping the current delivery implementation and evaluation of on-going training provision for CHWs in LMICs.

Design: Systematic scoping review.

Data sources: Medline, Embase, AMED, Global Health, Web of Science, Scopus, ASSIA, LILACS, BEI and ERIC.

Study selection: Original studies focussing on the provision of on-going training for CHWs working in a country defined as low- and middle-income according to World Bank Group 2012 classification of economies.

Results: The scoping review found 35 original studies that met the inclusion criteria. On-going training activities for CHWs were described as supervision (n=19), in-service or refresher training (n=13), or a mixture of both (n=3). Although the majority of studies emphasised the importance of providing on-going training, several studies reported no impact of on-going training on performance indicators. The majority of on-going training was delivered in-person, however four studies reported the use of mobile technologies to support training delivery. The outcomes from on-going training activities were measured and reported in different ways, including changes in behaviour, attitudes and practice measured in a quantitative manner (n=16), knowledge and skills (n=6), qualitative assessments (n=5) or a mixed methods approach combining one of the aforementioned modalities (n=8).

Conclusions: This scoping review highlights the diverse range of on-going training for CHWs in LMICs. Given the expansion of CHW programmes globally, more attention should

For peer review only

be given to the design, delivery, monitoring and sustainability of on-going training from a health systems strengthening perspective.

Keywords: Global Health, Community Health Worker (CHW), Lay health worker, Education, On-going training, Refresher training, In-service training, Supportive Supervision, Low- and middle-income country (LMIC)

Strengths and limitations of this study

- The study searched 10 major databases and additional non-peer reviewed literature in order to include as many relevant studies as possible.
- This is one of the first known reviews to assess the provision of on-going training to CHWs in LMICs.
- Methodological quality assessment of the studies included did not take place, since this was a scoping review.
- Due to the exclusion criteria and search strategy some NGO or IGO reports may not have been included in this review.
- There is no fixed definition of a CHW, and so some exclusions based on terminology may be debated.

Funding statement: This research received no specific grant from any funding agency in the public, commercial or not-for-profit sectors', however Dr. James O'Donovan is a DPhil candidate at The University of Oxford and is supported by a personal expenses and research support grant from the Economic and Social Research Council (ES/P000649/1).

Competing interests statement: "All authors have completed the ICMJE uniform disclosure form at www.icmje.org/coi_disclosure.pdf and declare: Dr. O'Donovan reports grants and personal fees from the Economic and Social Research Council, during the conduct of the study; no financial relationships with any organisations that might have an interest in the

For peer review only

submitted work in the previous three years; no other relationships or activities that could appear to have influenced the submitted work."

For peer review only

Introduction

The World Health Organization (WHO) have forecast a global shortage of 18 million health workers by 2030.[1] One solution to address this gap has been to advocate for the recruitment, training and deployment of Community Health Workers (CHWs) in low- and middle-income countries (LMICs).[2] In the broadest sense, CHW is an umbrella term for lay people working within their own community in a health promotion, prevention and delivery role,[3] however the nomenclature used to describe CHWs is wide ranging and their exact roles, responsibilities, recruitment, remuneration and training vary from country to country.[4,5] When provided with the correct resources, training, and support, CHWs have been proven to help improve health outcomes and accessibility to basic services.[2,6,7]

The WHO have suggested that for CHWs to fulfil their role successfully, they require "regular training and supervision".[8] For the purpose of this scoping review, we will focus specifically on evaluating the provision of on-going training for CHWs, rather than initial or pre-service training, since on-going training has typically been "the most neglected phase" of training,[9] with significant variability in terms of how it is delivered.[10] On-going training includes 'in-service' or 'refresher' training, defined as "follow on training received after a period of initial training",[11] or supportive supervision, defined as "a process of helping staff to improve their own work performance continuously… with a focus on using supervisory visits as an opportunity to improve knowledge and skills."[12]

Despite the importance placed on on-going training,[10] there is significant variation both in terms of its frequency, content, structure and monitoring between the different groups responsible for training CHWs.[10,13-15] For example, a study by Singh et al., found there were 22 different designated organisations responsible for training CHWs in Uganda.[13] The study also found that many of these organisations did not have specific training on "when, what and

For peer review only

how to supervise" CHWs.[13]

The frequency with which on-going training is provided appears to vary significantly between different organisations and countries. Guidelines produced by the USAID Health Care Improvement Project, recommend that refresher training should be provided at least every six months to update CHWs on new skills, reinforce initial training, and ensure they are practicing skills learned,[16] yet some CHWs have not had refresher training for over five years.[17] This finding of a poor provision of on-going training is commonplace and mentioned in several other studies, across multiple geographic contexts.[18-21] A multinational analysis from several countries in sub-Saharan Africa concluded that the current provision of refresher training courses was "not sufficient to meaningfully improve the quality of care in these countries," raising into question the need to assess the effectiveness of training programmes, both from the perspective of the individual CHW, but also the health system in which they operate.[22]

Although a systematic review was published in 2013 by Bluestone et al., evaluating effective in-service training design and delivery for health professionals more broadly,[23] there has been no review to specifically assess on-going training for CHWs in LMICs. A review published in 2014 by Hill et al., aimed to determine the impact of supportive supervision strategies for health workers in LMICs, however the scope of this review was relatively narrow, focusing just on supportive supervision, rather than on-going training more broadly and included multiple cadres of health workers.[24]

The aim of this systematic scoping review was therefore to map the current delivery, implementation and evaluation of on-going training provision for CHWs in LMICs.

For peer review only

Methods

Review approach

We conducted a systematic scoping review on the provision of on-going training for CHWs in LMICs. A scoping review is defined as "a form of knowledge synthesis that addresses an exploratory research question aimed at mapping key concepts, types of evidence, and gaps in research related to a defined area or field by systematically searching, selecting, and synthesizing existing knowledge".[25] Scoping reviews are part of the family of research synthesis methods, but compared to systematic reviews address broader research questions. They aim to provide an overview and organisation of existing knowledge rather than a narrow synthesis of a predefined research question,[26,27] and place less emphasis on the critical appraisal of the included evidence compared to a traditional systematic review.[28]

A scoping literature review was chosen for this study since we wished to discover the gaps in the literature with regards to the provision of on-going training for CHWs in LMICs - an area that has not been reviewed before. This approach also enabled us to review a broad body of literature to better understand the current landscape of on-going training across a variety of contexts. This included mapping the extent, range and nature of how on-going training is provided and what future research needs to be undertaken.

A review protocol was not published, and the study was not registered with PROSPERO, as these mechanisms are not applied to scoping reviews.[25,26] Nonetheless, our scoping review followed explicit and transparent research steps to explore the research evidence on on-going training for CHWs in LMICs.

Search Strategy and Selection Criteria

The Cochrane Library, The Campbell Collaboration and The International Prospective Register of Systematic Reviews (PROSPERO) and grey literature were searched to identify

For peer review only

available or on-going systematic reviews pertaining to the provision of on-going training for CHWs in LMICs. No previous or on-going relevant reviews were identified.

We then designed an exhaustive and sensitive search strategy to identify all relevant studies. The search was developed with and reviewed by a medical librarian (IK) to ensure completeness. The search strategy was deliberately designed to be over inclusive. 37 relevant search terms for 'Community Health Workers' and 'on-going training' were developed (*see Table 1 in the supplementary material for the full list of terms used within the search strategies*). These were combined with the World Bank Group 2012 list of LMICs[29] using the AND boolean operator to develop a master search string. Where appropriate, each index-linked MeSH term was exploded to contain all relevant subheadings. In addition, synonyms were searched for each key term, along with wildcards and truncation for free text words. A full record of the conducted search for each database is provided in the Supplementary Material. The following databases were searched to identify primary, peer-reviewed studies published from 12th September 1978, up to and including July 10th 2017:

- Medline;
- Embase and AMED via Ovid;
- Global Health via Ebsco;
- Web of Science;
- Scopus;
- ASSIA via ProQuest;
- LILACS;
- British Education Index;
- ERIC

For peer review only

We wanted to ensure coverage of the relevant literature and education and the social sciences as well as medical sciences, hence including ERIC, BEI, ASSIA and Web of Science. We also wanted to ensure broader coverage of global literature, hence the inclusion of LILACS, which give extensive coverage of Latin America and the Caribbean. The 12th of September 1978 was chosen as a cut-off date since this was the date of the Alma Ata Declaration, which identified CHWs as "one of the cornerstones of comprehensive primary health care".[8]

Despite issues relating to data quality, we included non-peer reviewed literature in this review in order to encapsulate a broad overview of the literature pertaining to refresher training for CHWs in LMICs. To identify relevant additional non-peer reviewed literature, we used the following sources; e-theses online service (ETHoS), conference proceedings on Index of Conference proceedings and Google Scholar. Finally, we also searched the reference lists of all relevant papers that we identified, using snowball sampling.

Inclusion and exclusion criteria

Studies were included if:

1. The primary participants were CHWs;
2. The CHWs worked in a country defined as low- or middle-income according to World Bank Group 2012 classification of economies;
3. It was explicitly stated that the objectives or aims of the study were to evaluate or assess the provision of on-going training, which could include refresher training, in-service training, continuing training or supportive supervision.

Studies were excluded if:

1. The primary focus of the paper was on health care professionals other than CHWs - for example, doctors, medical students, nurses, or allied healthcare professionals, such as midwives or community based physician's assistants, were excluded.

For peer review only - http://bmjopen.bmj.com/site/about/guidelines.xhtml

2. The study was not conducted in a country defined as a LMIC according to World Bank Group 2012 classification of economies;
3. The paper was not an original, full text, research study. For example, commentaries, letters, opinion pieces, study protocols, training needs assessments and conference proceedings with only an abstract available, were all excluded.
4. The focus of the study was primarily on initial or pre-service training, rather than on-going training;
5. As part of the screening process during the full text review stage, studies were excluded if they did not report or describe the following three areas: (i) the design, (ii) the duration and frequency, and (iii) the outcomes, of the on-going training programme. It was deemed necessary that these three areas were commented upon in order that we had sufficient detail about the on-going training programme from which to base our analysis. These were also good screening questions from which to exclude studies for which the description and evaluation of on-going training was not the primary focus of the study, but rather was just mentioned briefly or in passing.

Since the aim of our scoping review was to map the existing literature regarding the provision, design and outcomes of on-going training, both qualitative and quantitative study designs were included. Studies did not require a comparison group for inclusion.

Population: Although the nomenclature given to CHWs varies across the literature, for the purpose of this study we referred to the 2007 WHO definition:

"Community health workers should be members of the communities where they work, should be selected by the communities, should be answerable to the communities for their activities, should be supported by the health system but not necessarily a part of its organization, and have shorter training than professional workers."[30]

For peer review only

This definition allows for different types of health care workers to be classified as CHWs in different contexts. To clarify the ambiguity surrounding the term 'shorter training' given in the description above, we followed the definition from Lewin et al., to define shorter training as: "no formal professional or paraprofessional certificated or degreed tertiary education."[31]

Intervention: Studies had to focus on the provision of on-going training. For this review, on-going training is an umbrella term referring to any type of training a CHW can receive after a period of initial training. This can include refresher training, continuing training, in-service training or supportive supervision. We purposely aimed to encapsulate a broad range of on-going training subtypes, so as to better understand the current state of the field.

Research design: To be included, studies had to qualify as an original, full text, research study. This meant that review articles, commentaries, letters, policy briefs, protocols, training needs assessments and conference abstracts were not included. Generally, the original article had to include an introduction, explicitly state the aims of the study were to evaluate the provision of on-going training, and include a methods, results and discussion section to allow us to extract the necessary data for the questions we set out to answer.

Outcomes: No studies were excluded based on the measured outcomes, since one of the primary aims of this scoping review was to determine which measures are used to report the outcomes of on-going training programmes.

Study selection

All papers identified via database searching were exported into EndNote 7.1 and duplicate references removed. Titles and abstracts of all publications identified in the search were screened by two authors (JOD and COD). This determined whether they would be considered for a full text review. Those that were clearly irrelevant to the topic of this study were discarded at this stage. The full text of all the papers identified as potentially relevant by one

For peer review only

or both review authors was then retrieved and reviewed in full against the inclusion and exclusion criteria. At all stages, disagreements between the review authors were resolved via discussion or, if required, by seeking a third review from an independent researcher. The independent researcher was always the same person and was not part of the direct research team listed in this study. Where appropriate, we contacted the authors of individual studies for further information.

Data analysis

Once studies were determined to have met the inclusion criteria, the relevant data was systematically extracted from each study and tabulated using a 'data charting form' in a Microsoft Excel spreadsheet by one author (JOD). The data extracted from each study included the study author, title, date, country and region which the study took place, CHW name and cadre description, the number of CHWs who took part in the study, the disease focus area, a description of how the on-going training programme was delivered, as well as a report on the outcomes measured. The use of a 'data charting form' has been recommended by Arksey & O'Malley and Levac et al., as a key stage of conducting a scoping review.[26,32] Where necessary, the corresponding authors for relevant studies were contacted via email to clarify aspects of their work prior to final inclusion.

Once the data had been transferred into the spreadsheet, two authors (JOD & NW) reviewed the information and selected key focus areas for the review, as well as categories for the outcome reporting methods. The same two authors thematically grouped outcome data from on-going training into one of the following four categories: 1. Knowledge and Skills Assessments 2. Changes in Behaviour, Attitudes or Practice 3. Qualitative Assessments 4. Mixed Methods Approaches. Similarly, if the use of mobile technologies was noted in the

For peer review only - http://bmjopen.bmj.com/site/about/guidelines.xhtml

study, this was documented and categorised using the mHealth framework developed by Labrique et al.[33]

Patient and Public Involvement

Patients and the public were not involved in this scoping review.

For peer review only

Results

Search results

The initial search of the ten databases yielded 3923 articles (see Table 2, Supplementary Material). After exclusion of duplicate references using the EndNote referencing system, 2609 papers were identified for initial screening. After the initial abstract and title screen, 172 studies were identified for full text review. Following this review, 137 papers were excluded. Reasons for exclusion at full-text screening can be found in the PRISMA flowchart (Figure 1). As a result, we were left with 35 original studies meeting the inclusion criteria.[13-15,34-65]

CHW cadres and study characteristics

22 different terms were identified as defining CHWs across the 35 studies, with significant variations being noted between studies in terms of CHW roles, responsibilities and status. The majority of studies evaluating the provision of on-going training for CHWs have been published since 2015 (n=19), with no relevant studies published before 1993. In terms of geographic location, most studies took place in East Africa, (n=16) or South Asia (n=7). For full details regarding CHW cadre descriptions and study characteristics, please refer to Table 3 in the supplementary material.

On-going training details

The reported type, frequency, duration, training focus and outcomes of on-going training delivery were highly variable between studies (see Table 3, Supplementary Material). For example, Zeitz et al., reported on a one-day refresher training course for CHWs which specifically focused on acute respiratory illness in children and used pre and post testing of knowledge as the outcome measure of the training.[65] This is in contrast with Kawasaki et al., who carried out a two-year study where CHWs received monthly refresher trainings and the

For peer review only - http://bmjopen.bmj.com/site/about/guidelines.xhtml

outcome measures were focused on behaviour change at the community level; for example improved handwashing techniques and the number of household visits carried out by CHWs.[48] This variation, both in terms of duration, structure and content focus makes direct comparison between studies difficult (see Table 3 in the supplementary material).

Oliver et al., highlighted the importance of co-designing programmes with CHWs to help ensure relevance to their practices and experiences.[66] This scoping review revealed a lack of participatory input from key stakeholders in the design and delivery of the training programmes. Only five studies documented seeking input from CHWs in the design of the training programmes.[13,14,48,56,57] For example, Puchalski Ritchie et al., stated that the training content was developed based on the training needs identified by CHWs in a qualitative survey prior to the programme being established.[56,57] They also mentioned that the training sessions and tools were chosen in consultation with local collaborators and the local language was used in the study.[64,67]

With regards to programme delivery, most training was delivered in-person, with four studies reporting the use of mobile technologies to deliver or assist on-going training activities.[14,47,59,64] To report the outcomes of on-going training programmes, a range of different measures were used. The majority of studies evaluated the effect of on-going training by using proxy markers to assess change in practice, attitudes or behaviour (n=16).[13,34-36,38,47-49,52-54,56,61-64] This included assessing behaviour change at a community level, such as improved vaccination uptake and hand washing amongst households,[13] to changes in practice among CHWs, such as improved record keeping.[47] Assessment of knowledge and skills, mainly through the use of pre- and post-intervention tests, formed the sole means of evaluation in six of the studies.[40-43,65,67] Five studies used a qualitative approach for programme evaluation, mainly through the use of interviews and focus discussion groups, which were then thematically analysed and reported.[14,39,46,57,59] Eight

For peer review only - http://bmjopen.bmj.com/site/about/guidelines.xhtml

studies adopted a mixed methods approach, using a combination of knowledge and skills assessments, qualitative approaches or changes in behaviour, attitudes and practice.[15,37,45,50,51,55,58,60] The outcomes reported were variable given the heterogeneity of the approaches to evaluation, however the majority of studies reported positive outcomes following on-going training; for example Horwood et al. found that children managed by a CHW who had attended a refresher training session were more likely to be managed correctly according to iCCM guidelines compared to those who had not.[45] Similarly, a study by Singh et al., found that homes in areas where CHWs had received supportive supervision were more likely to have installed and functioning tippy taps for hand washing, compared to areas served by CHWs who had not received supervision.[13] Yet despite the many positive outcomes associated with on-going training, there were also studies that found no difference in outcome measures between CHWs who received on-going training and those who had not and there were even negative reports of on-going training. One such example of this was the study by Javanparast et al., which revealed that CHWs were dissatisfied with on-going training in its current format, in particular "its quality and timing, the infrequency of courses, inadequately qualified trainers who are unfamiliar with the behvarz (CHWs) working environment, the lack of practical sessions and of physical space and training facilities".[46] Similarly, Ndima et al., found that CHWs in Mozambique felt their supportive supervision was poorly organised, causing them to feel demotivated, with their supervisors citing high concurrent workloads and a lack of support.[15]

For full details of the outcomes for individual studies please refer to Table 3 in the supplementary material.

For peer review only

Discussion

There is a diverse range of approaches in the design, delivery and reported outcomes of on-going training for CHWs in LMICs and a number of significant gaps remain.

Location, content and duration of on-going training programmes

The majority of studies describing on-going training for CHWs have a narrow geographic concentration. Given the highly contextualised role of the CHW,[68] this presents an opportunity for further research to be carried out in other geographical contexts. Furthermore, the majority of studies focused on the provision of on-going training for maternal and child health, or infectious diseases such as HIV and TB. Given the combined shortage of a lack of specialist health workers and the high morbidity and mortality from the aforementioned disease groups, CHWs have rightly been trained to address these issues. Although the burden of infectious disease and child and maternal health remain problematic in LMIC settings, no studies focussed on the provision of on-going training for non-communicable diseases (NCDs) and only one study focussed on the provision of on-going training for CHWs involved in mental health care.[51] NCDs have been described as the "social justice issue of our time,"[69,70] since they disproportionally affect populations in LMICs.[71] It is therefore imperative that more attention is directed towards providing on-going training in the prevention and management of NCDs at a community level if we are to make realistic progress towards SDG 3.4, which has set the target of reducing premature mortality from NCDs by a third, by 2030.[72] This public health need to expand CHW provision towards NCDs is both an opportunity and challenge, since it will require the commitment of governments, funders and program managers to retrain and refocus large CHW workforces.

Delivery of on-going training programmes

The majority of on-going training was delivered in person, with only four studies reporting the use of mobile technologies as playing a role in training delivery. This was a surprising finding since mobile technologies have been used as a mean to train other cadres of healthcare professionals in LMICs.[73-75] Given the high ownership of mobile phones in sub-Saharan Africa,[76] and the ability for flexible learning, data collection,[77] the use of mHealth to facilitate on-going training warrants exploration.[78] One of the studies included in this review highlighted the role of mobile phones to strengthen supportive supervision for CHWs in Kenya.[14] A WhatsApp group to facilitate instant messaging was created for CHWs and their supervisors to “support supervision, professional development, and team building”.[14] Importantly the authors of this study reported not only on the quality assurance and information exchange, which the system facilitated, but also on the supportive environment fostered by the use of the technology.[14]

Given that several studies cited supervisors high concurrent workloads as to why on-going training was poorly organised and delivered,[15,39,46,60] mHealth should be explored further as a potential tool to manage human resource shortages, since this is one of the key applications of mHealth tools mentioned by Labrique et al., as a health systems strengthening innovation.[33]

As a caveat, Hampshire et al., have urged researchers and practitioners to proceed with caution and consider the financial implications when considering mobile technologies as a training tool for CHWs, due to the potential risk of reinforcing socioeconomic, geographical and gender inequalities.[79] Furthermore, Joos et al., highlighted the need to consider how mobile phones can successfully transition to scale following pilot studies.[47]

Outcome measures and outcomes of on-going training

Given the variation of how on-going training programme outcomes were evaluated and reported, direct comparison between studies is difficult.

For outcome reporting, 16 studies used markers of behaviour change at the household level or CHW practice to measure the impact of on-going training. Using measures of behaviour change to evaluate the effectiveness of on-going training is a welcome move towards ensuring meaningful programme evaulation,[80,81] however researchers and programme managers should be aware of the multiple confounding variables that could influence these behaviors, such as the Hawthorne effect, and the difficulty in assessing these practices longitudinally, as well as the need to approach programme evaluations from a complex interventions standpoint.[82]

Similarly, where pre-and-post tests of knowledge and skills acquisition are used to evaluate the impact of on-going training programmes, they do not necessarily reflect the abilities of CHWs to perform their role well in the community,[83] nor do they provide any insight into CHWs experiences of training. Hamilton and Friesen, argue that instrumental views of assessing learning often fail to capture the practical and emancipatory concerns of learners,[84] and thus alternative methods of evaluation should be explored.[85] Furthermore, it is important to consider the validity and applicability of such tests to real life settings, given that many of the assessment tools have been designed by the researchers and are unvalidated. What is more, some CHWs have only been in formal education to the level of primary or secondary school, and so this form of assessment may introduce construct-validity bias. Interestingly, Rowe et al., who used a skills and knowledge assessment tool and found no improvement in scores between the groups of CHWs who took part in refresher training and those who did not.[61] They questioned the usefulness of refresher training based on this outcome, however failed to acknowledge the other benefits of on-going training which they did not measure, such as an improved sense of community, motivation and empowerment.[86]

For peer review only - http://bmjopen.bmj.com/site/about/guidelines.xhtml

Puchalski Richie et al., actively avoided using an “assessment of knowledge and skills”[56] since they were concerned that it might negatively affect participation in training; instead, they carried out a qualitative evaluation of CHWs overall satisfaction with the program as a measure of training success.[57] Similarly other studies, which used a qualitative approach to outcomes evaluation, found that CHWs had negative experiences of on-going training - insights that would not have necessarily be revealed if a purely empirical approach was taken towards programme evaluation. [87] A mixed methods approach towards evaluation may therefore be a useful approach for future studies.

No studies used the framework for outcome-level evaluation of in-service training of healthcare workers produced by O’Mallay et al., in 2013.[88] This framework was developed in a holistic manner to evaluate in-service training of health workers based on the needs of the individual, the organisation and the health system. Current assessment of in-service training programme assessment relies heavily on measuring and reporting training “outputs” such as the number of CHWs trained, the total hours of training delivered, and scores obtained on standardised tests.

A small number of studies used self-reported satisfaction,[64] motivation[58] or increased agency[57] as outcomes to measure the impact of on-going training. These are what Kok refers to as “software” of a training programme and can affect motivation and performance.[89] Kok argues that the software elements of the system are important since they effect CHW performance by “influencing self-esteem, attitudes and agency,”[89] as well as satisfaction and motivation. Ndima et al., commented that when training focuses too heavily on developing technical skills there is a danger that “examining value and attitudes of CHWs and abilities to understand and support individual and group dynamics”[15] can be lost.

Participatory approaches to on-going training design, delivery and evaluation

For peer review only - http://bmjopen.bmj.com/site/about/guidelines.xhtml

Given the lack of documented participant input and feedback in terms of program design, delivery and evaluation, research into the use of participatory action research (PAR) is one area that would warrant further investigation. PAR broadly involves working 'with' end-users in a collaborative effort rather than 'for' or 'on' them.[90] It encapsulates the ideals of promoting autonomy and social justice, and works on the principle that the end-users wishes and needs, should be respected and valued.[90] This school of thought was echoed by Perry and Crigler, who advised a "top-down supervisory approach…may not be as feasible or effective as a participatory supervision model where CHWs and their communities are provided with the resources and autonomy to seek out the support that they need to perform well and stay motivated."[91]

It is also important to consider socio-cultural sensitivities in the design of an on-going training intervention, including cultural beliefs, especially in areas where the practice of traditional medicine is still commonplace and may be at odds with a more Western approach to healthcare. In the study by Singh et al., the training intervention was delayed by four months due to villagers believing the immunizations used by the CHWs were intended to cause infertility and the insecticide treated bednets were designed to 'kill their children'.[13] This is especially relevant when on-going training programmes are being designed and implemented by non-native researchers, in countries emerging from post-colonial pasts and where local beliefs are rooted in historical antecedants.[92]

Study limitations

It is important to recognise that given the highly contextualised nature of CHW training programmes[21,68] this scoping review does not try to address best practice or provide guidelines. Rather, we have attempted to map the current landscape of on-going training for CHWs in order to broadly identify key similarities or differences between on-going training

For peer review only

programmes and identify areas that may have received little attention in the literature to date to help inform other researchers, practitioners or policy makers working in this field.

We tried to be as inclusive as possible to identify relevant literature, but with the diverse range of terms used to describe CHWs, it is possible we have inadvertently missed out some eligible studies describing on-going training for CHWs. Further, we did not conduct an exhaustive search for grey literature sources due to the challenges in appraising these types of publications as well as the lack of standardised search guidelines for scoping reviews.[93]

Finally, given the nature of scoping reviews, a critical appraisal of the studies included in the review was not performed.[26] This could be perceived as a limitation since the overall quality and level of detail of the studies was variable. There was also significant heterogeneity between studies, which makes direct comparisons difficult. Future work should aim to clearly outline the context in which CHWs work and provide a detailed description of their job roles and responsibilities to help orientate the reader and contextualise the setting.

For peer review only

Conclusions

There is significant variability between on-going training programmes for CHWs in LMICs, both in terms of design, structure, content, duration and reported outcomes. This fragmented approach means little is understood about how to best deliver on-going training in LMICs. On-going training programmes have largely taken an empirical approach, focussing on specific areas, for example child and maternal health and infectious diseases, in limited geographic contexts and have variable approaches towards outcome measurement and reporting. The danger is that this approach fails to acknowledge what Kim, Farmer and Porter refer to as the "broader systems and conditions affecting global health-care delivery."[81] Given the heterogeneity of the field we advocate for a realist approach to evaluation for future research, considering training as a complex intervention. This may help those interested in the field to make better sense of its complex nature with a view to understanding what works, for whom, and under what conditions. Through taken this approach and considering the contextual requirements, on-going training programmes are more likely to contribute to a systems level improvement in resource limited settings.

For peer review only

Figure Legends:

Figure 1. PRISMA flow diagram. The PRIMSA diagram details our search and selection process applied during the scoping review.

Contributor Statement: Activities undertaken by the authors were as follows: Establishment of research question(s) and development of search strategy: JOD, IK and NW. Background framing: JOD & NW. Database search and record screening: JOD, COD & IK. Extraction of primary studies from the included reviews: JOD and COD. Discussion: JOD, COD, NW, SS.

Acknowledgements: We wish to thank Miss. Stephanie Sobek for proofreading the paper prior to final submission.

Data sharing: All data is contained within the main body of the text and in the on-line supplementary material.

For peer review only

References

1. Darzi A, Evans T. The global shortage of health workers-an opportunity to transform care. *Lancet.* 2016;388(10060):2576-2577.
2. Perry HB, Zulliger R, Rogers MM. Community health workers in low-, middle-, and high-income countries: an overview of their history, recent evolution, and current effectiveness. *Annu Rev Public Health.* 2014;35:399-421.
3. Turinawe EB, Rwemisisi JT, Musinguzi LK, et al. Selection and performance of village health teams (VHTs) in Uganda: lessons from the natural helper model of health promotion. *Hum Resour Health.* 2015;13:73.
4. Bhattacharyya K, Winch P, LeBan K, M. T. *Community Health Worker Incentives and Disincentives: How They Affect Motivation, Retention, and Sustainability. Basic Support for Institutionalizing Child Survival Project (BASICS II).* Virginia, USA: USAID;2001.
5. Olaniran A, Smith H, Unkels R, Bar-Zeev S, van den Broek N. Who is a community health worker? - a systematic review of definitions. *Glob Health Action.* 2017;10(1):1272223.
6. Prinja S, Jeet G, Verma R, et al. Economic analysis of delivering primary health care services through community health workers in 3 North Indian states. *PLoS One.* 2014;9(3):e91781.
7. Vaughan K, Kok MC, Witter S, Dieleman M. Costs and cost-effectiveness of community health workers: evidence from a literature review. *Hum Resour Health.* 2015;13:71.
8. The World Health Organization. Community health workers: What do we know about them? The state of the evidence on programmes, activities, costs and impact on health outcomes of using community health workers. 2007; http://www.who.int/hrh/documents/community_health_workers_brief.pdf Accessed 05/11/2017.
9. Li VC, Goethals PR, Dorfman S. A global review of training of community health workers, 1983-84. *Int Q Community Health Educ.* 2006;27(3):181-218.
10. Redick C, Faich Dini HS, Long L-A. *The Current State of CHW Training Programs in Sub-Saharan Africa and South Asia: What We Know, What We Don't Know, and What We Need to Do.* 2014.
11. Hopkins D. *Inservice training and educational development: An International Survey* New Hampshire, USA,1986.
12. The World Health Organization. Training for mid-level managers (MLM) Module 4: Supportive supervision. 2008; http://www.who.int/immunization/documents/MLM_module4.pdf. Accessed 05/11/2017.
13. Singh D, Negin J, Orach CG, Cumming R. Supportive supervision for volunteers to deliver reproductive health education: a cluster randomized trial. *Reproductive Health.* 2016;13(1):126.
14. Vu Henry J, Winters N, Lakati A, et al. Enhancing the Supervision of Community Health Workers With WhatsApp Mobile Messaging: Qualitative Findings From 2 Low-Resource Settings in Kenya. *Glob Health Sci Pract.* 2016;4(2):311-325.
15. Ndima SD, Sidat M, Give C, Ormel H, Kok MC, Taegtmeyer M. Supervision of community health workers in Mozambique: a qualitative study of factors influencing motivation and programme implementation. *Human Resources for Health [Electronic Resource].* 2015;13:63.
16. Crigler L HK, Furth R, Bjerregaard D. Community Health Worker Assessment and Improvement Matrix (CHW AIM): A Toolkit for Improving Community Health Worker Programs and Services USAID Health Care Improvement Project. 2011; http://www.who.int/workforcealliance/knowledge/toolkit/50.pdf. Accessed 17/8/2017.

For peer review only - http://bmjopen.bmj.com/site/about/guidelines.xhtml

17. Wanduru P, Tetui M, Tuhebwe D, et al. The performance of community health workers in the management of multiple childhood infectious diseases in Lira, northern Uganda - a mixed methods cross-sectional study. *Glob Health Action.* 2016;9:33194.
18. Stellenberg E, Van Zyl M, Eygelaar J. Knowledge of community care workers about key family practices in a rural community in South Africa. *African Journal of Primary Health Care & Family Medicine.* 2015;7(1):892.
19. Sipsma HL, Curry LA, Kakoma JB, Linnander EL, Bradley EH. Identifying characteristics associated with performing recommended practices in maternal and newborn care among health facilities in Rwanda: a cross-sectional study. *Human Resources for Health.* 2012;10.
20. Sheikh S, Qureshi RN, Khowaja AR, et al. Health care provider knowledge and routine management of pre-eclampsia in Pakistan. *Reproductive Health.* 2016;13(Suppl 2):104.
21. Blacklock C, Goncalves Bradley DC, Mickan S, et al. Impact of Contextual Factors on the Effect of Interventions to Improve Health Worker Performance in Sub-Saharan Africa: Review of Randomised Clinical Trials. *PLoS One.* 2016;11(1):e0145206.
22. Leslie HH, Gage A, Nsona H, Hirschhorn LR, Kruk ME. Training And Supervision Did Not Meaningfully Improve Quality Of Care For Pregnant Women Or Sick Children In Sub-Saharan Africa. *Health Affairs.* 2016;35(9):1716-1724.
23. Bluestone J, Johnson P, Fullerton J, Carr C, Alderman J, BonTempo J. Effective in-service training design and delivery: evidence from an integrative literature review. *Hum Resour Health.* 2013;11:51.
24. Hill Z, Dumbaugh M, Benton L, et al. Supervising community health workers in low-income countries--a review of impact and implementation issues. *Glob Health Action.* 2014;7:24085.
25. Colquhoun HL, Levac D, O'Brien KK, et al. Scoping reviews: time for clarity in definition, methods, and reporting. *J Clin Epidemiol.* 2014;67(12):1291-1294.
26. Arksey H, O'Malley L. Scoping studies: towards a methodological framework. *International Journal of Social Research Methodology.* 2005;8(1):19-32.
27. Miake-Lye IM, Hempel S, Shanman R, Shekelle PG. What is an evidence map? A systematic review of published evidence maps and their definitions, methods, and products. *Syst Rev.* 2016;5:28.
28. Haby MM, Chapman E, Clark R, Barreto J, Reveiz L, Lavis JN. What are the best methodologies for rapid reviews of the research evidence for evidence-informed decision making in health policy and practice: a rapid review. *Health Res Policy Syst.* 2016;14(1):83.
29. The World Bank. World Bank Country and Lending Groups. 2012 http://data.worldbank.org/about/country-classifications. Accessed 07/11/2017.
30. The World Health Organization. *Strengthening the performance of community health workers in primary health care. Report of a WHO Study Group.* WHO Technical Report Series, No. 780 Geneva World Health Organization 1989.
31. Lewin SA, Dick J, Pond P, et al. Lay health workers in primary and community health care. *Cochrane Database Syst Rev.* 2005(1):CD004015.
32. Levac D, Colquhoun H, O'Brien KK. Scoping studies: advancing the methodology. *Implement Sci.* 2010;5:69.
33. Labrique AB, Vasudevan L, Kochi E, Fabricant R, Mehl G. mHealth innovations as health system strengthening tools: 12 common applications and a visual framework. *Glob Health Sci Pract.* 2013;1(2):160-171.
34. Adejumo AO, Azuogu B, Okorie O, et al. Community referral for presumptive TB in Nigeria: a comparison of four models of active case finding. *BMC Public Health.* 2016;16:177.
35. Ameha A, Karim AM, Erbo A, et al. Effectiveness of supportive supervision on the consistency of integrated community cases management skills of the health extension workers in 113 districts of Ethiopia. *Ethiopian Medical Journal.* 2014;52 Suppl 3:65-71.
36. Ayele F, Desta A, Larson C. The functional status of community health agents: A trial of refresher courses and regular supervision. *Health Policy and Planning.* 1993;8(4):379-384.

For peer review only

37. Carlough M, McCall M. Skilled birth attendance: What does it mean and how can it be measured? A clinical skills assessment of maternal and child health workers in Nepal. *International Journal of Gynecology & Obstetrics.* 2005;89(2):200-208.
38. Das A, Friedman J, Kandpal E, et al. Strengthening malaria service delivery through supportive supervision and community mobilization in an endemic Indian setting: an evaluation of nested delivery models. *Malar J.* 2014;13:482.
39. Datiko DG, Yassin MA, Tulloch O, et al. Exploring providers' perspectives of a community based TB approach in Southern Ethiopia: implication for community based approaches. *BMC Health Serv Res.* 2015;15:501.
40. Dewing S, Mathews C, Cloete A, Schaay N, Simbayi L, Louw J. Lay counselors' ability to deliver counseling for behavior change. *J Consult Clin Psychol.* 2014;82(1):19-29.
41. Gallo MF, Walldorf J, Kolesar R, et al. Evaluation of a volunteer community-based health worker program for providing contraceptive services in Madagascar. *Contraception.* 2013;88(5):657-665.
42. Gupta DN, SenGupta PG, Sircar BK, Mondal S, Sarkar S, Deb BC. Implementation of ORT: some problems encountered in training of health workers during an operational research programme. *Indian Journal of Public Health.* 1994;38(2):69-72.
43. Gupta M, Tripathy JP, Jamir L, Sarwa A, Sinha S, Bhag C. Improving quality of home-based postnatal care by microteaching of multipurpose workers in rural and urban slum areas of Chandigarh, India: a pilot study. *Advances in Medical Education and Practice.* 2017;8:1-8.
44. Hadi A. Management of acute respiratory infections by community health volunteers: experience of Bangladesh Rural Advancement Committee (BRAC). *Bulletin of the World Health Organization.* 2003;81(3):183-189.
45. Horwood C, Butler L, Barker P, et al. A continuous quality improvement intervention to improve the effectiveness of community health workers providing care to mothers and children: a cluster randomised controlled trial in South Africa. *Hum Resour Health.* 2017;15(1):39.
46. Javanparast S, Baum F, Labonte R, Sanders D, Rajabi Z, Heidari G. The experience of community health workers training in Iran: a qualitative study. *BMC Health Services Research.* 2012;12:291.
47. Joos O, Silva R, Amouzou A, et al. Evaluation of a mHealth Data Quality Intervention to Improve Documentation of Pregnancy Outcomes by Health Surveillance Assistants in Malawi: A Cluster Randomized Trial. *PLoS One.* 2016;11(1):e0145238.
48. Kawasaki R, Sadamori T, Ferreira de Almeida T, et al. Reactions of community members regarding community health workers' activities as a measure of the impact of a training program in Amazonas, Brazil. *Journal of Rural Medicine.* 2015;10(1):7-19.
49. Kuule Y, Dobson AE, Woldeyohannes D, et al. Community Health Volunteers in Primary Healthcare in Rural Uganda: Factors Influencing Performance. *Frontiers in Public Health.* 2017;5:62.
50. Mash R, Baldassini G, Mkhatshwa H, Sayeed I, Ndapeua S, Mash B. Reflections on the training of counsellors in motivational interviewing for programmes for the prevention of mother to child transmission of HIV in sub-Saharan Africa. *South African Family Practice.* 2008;50(2):53-59.
51. McLean KE, Kaiser BN, Hagaman AK, Wagenaar BH, Therosme TP, Kohrt BA. Task sharing in rural Haiti: Qualitative assessment of a brief, structured training with and without apprenticeship supervision for community health workers. *Intervention (Amstelveen).* 2015;13(2):135-155.
52. Mengistu B, Karim AM, Eniyew A, et al. Effect of performance review and clinical mentoring meetings (PRCMM) on recording of community case management by health extension workers in Ethiopia. *Ethiop Med J.* 2014;52 Suppl 3:73-81.

For peer review only

53. Miller NP, Amouzou A, Hazel E, et al. Assessment of the impact of quality improvement interventions on the quality of sick child care provided by Health Extension Workers in Ethiopia. *J Glob Health.* 2016;6(2):020404.
54. Mkumbo E, Hanson C, Penfold S, Manzi F, Schellenberg J. Innovation in supervision and support of community health workers for better newborn survival in southern Tanzania. *Int Health.* 2014;6(4):339-341.
55. Msisuka C, Nozaki I, Kakimoto K, Seko M, Ulaya MM. An evaluation of a refresher training intervention for HIV lay counsellors in Chongwe District, Zambia. *SAHARA J: Journal of Social Aspects of HIV/AIDS Research Alliance.* 2011;8(4):204-209.
56. Puchalski Ritchie LM, Schull MJ, Martiniuk AL, et al. A knowledge translation intervention to improve tuberculosis care and outcomes in Malawi: a pragmatic cluster randomized controlled trial. *Implement Sci.* 2015;10:38.
57. Puchalski Ritchie LM, van Lettow M, Barnsley J, et al. Lay Health Workers experience of a tailored knowledge translation intervention to improve job skills and knowledge: a qualitative study in Zomba district Malawi. *BMC Medical Education.* 2016;16:54.
58. Rabbani F, Perveen S, Aftab W, Zahidie A, Sangrasi K, Qazi SA. Health workers' perspectives, knowledge and skills regarding community case management of childhood diarrhoea and pneumonia: a qualitative inquiry for an implementation research project "Nigraan" in District Badin, Sindh, Pakistan. *BMC Health Serv Res.* 2016;16:462.
59. Rabbani F, Shipton L, Aftab W, Sangrasi K, Perveen S, Zahidie A. Inspiring health worker motivation with supportive supervision: a survey of lady health supervisor motivating factors in rural Pakistan. *BMC Health Serv Res.* 2016;16(1):397.
60. Roberton T, Applegate J, Lefevre AE, et al. Initial experiences and innovations in supervising community health workers for maternal, newborn, and child health in Morogoro region, Tanzania. *Hum Resour Health.* 2015;13:19.
61. Rowe SY, Olewe MA, Kleinbaum DG, et al. Longitudinal analysis of community health workers' adherence to treatment guidelines, Siaya, Kenya, 1997-2002. *Tropical Medicine and International Health.* 2007;12(5):651-663.
62. Sylla A, Guèye EHB, N'Diaye O, et al. Low level educated community health workers training: a strategy to improve children access to acute respiratory treatment in Senegal. *Archives de Pediatrie.* 2007;14(3):244-248.
63. Talukder S, Farhana D, Vitta B, Greiner T. In a rural area of Bangladesh, traditional birth attendant training improved early infant feeding practices: a pragmatic cluster randomized trial. *Matern Child Nutr.* 2017;13(1).
64. Vallieres F, McAuliffe E, van Bavel B, Wall PJ, Trye A. There's No App for That: Assessing the Impact of mHealth on the Supervision, Motivation, Engagement, and Satisfaction of Community Health Workers in Sierra Leone. *Ann Glob Health.* 2016;82(5):936-949.
65. Zeitz PS, Harrison LH, Lopez M, Cornale G. Community health worker competency in managing acute respiratory infections of childhood in Bolivia. *Bull Pan Am Health Organ.* 1993;27(2):109-119.
66. Oliver M, Geniets A, Winters N, Rega I, Mbae SM. What do community health workers have to say about their work, and how can this inform improved programme design? A case study with CHWs within Kenya. *Glob Health Action.* 2015;8:27168.
67. Hadi A. Management of acute respiratory infections by community health volunteers: experience of Bangladesh Rural Advancement Committee (BRAC). *Bull World Health Organ.* 2003;81(3):183-189.
68. Dieleman M, Gerretsen B, van der Wilt GJ. Human resource management interventions to improve health workers' performance in low and middle income countries: a realist review. *Health Res Policy Syst.* 2009;7:7.
69. Saligram N, Nugent R. Chronic diseases--the social justice issue of our time. *Lancet.* 2016;387(10022):943.

For peer review only

70. Casswell S. Chronic diseases--the social justice issue of our time. *Lancet.* 2016;387(10022):942-943.
71. The World Health Organization. Global Health Observatory data - NCD mortality and morbidity. 2015 http://www.who.int/gho/ncd/mortality_morbidity/en/. Accessed 14/11/2017.
72. United Nations Development Programme. Sustainable Development Goals - SDG 3. 2017 https://sustainabledevelopment.un.org/sdg3. Accessed 14/11/2017
73. O'Donovan J, Bersin A, O'Donovan C. The effectiveness of mobile health (mHealth) technologies to train healthcare professionals in developing countries: a review of the literature. *BMJ Innovations.* 2015;1(1):33-36.
74. Singh P, Sachs JD. 1 million community health workers in sub-Saharan Africa by 2015. *Lancet.* 2013;382(9889):363-365.
75. Diedhiou A, Gilroy KE, Cox CM, et al. Successful mLearning Pilot in Senegal: Delivering Family Planning Refresher Training Using Interactive Voice Response and SMS. *Glob Health Sci Pract.* 2015;3(2):305-321.
76. GSMA Intelligence. The Mobile Economy, SubSaharan Africa 2015. 2015; https://www.gsma.com/mobileeconomy/archive/GSMA_ME_SubSaharanAfrica_2015.pdf. Accessed 23/11/2017.
77. Gordon N. Flexible Pedagogies: technology-enhanced learning. 2014; https://www.heacademy.ac.uk/system/files/resources/tel_report_0.pdf. Accessed 21/11/2017.
78. Langer L., Winters N., Stewart R. Mobile Learning for Development: Ready to Randomise? In: Kalz M., Bayyurt Y., Specht M., eds. *Mobile as a Mainstream – Towards Future Challenges in Mobile Learning. mLearn 2014. Communications in Computer and Information Science*: Springer, Cham 2014.
79. Hampshire K, Porter G, Mariwah S, et al. Who bears the cost of 'informal mhealth'? Health-workers' mobile phone practices and associated political-moral economies of care in Ghana and Malawi. *Health Policy Plan.* 2017;32(1):34-42.
80. A new institute for global health evaluations. *Lancet.* 2007;369(9577):1902.
81. Kim JY, Farmer P, Porter ME. Redefining global health-care delivery. *Lancet.* 2013;382(9897):1060-1069.
82. Gilmore B, Adams BJ, Bartoloni A, et al. Improving the performance of community health workers in humanitarian emergencies: a realist evaluation protocol for the PIECES programme. *BMJ Open.* 2016;6(8):e011753.
83. Abdel-All M, Putica B, Praveen D, Abimbola S, Joshi R. Effectiveness of community health worker training programmes for cardiovascular disease management in low-income and middle-income countries: a systematic review. *BMJ Open.* 2017;7(11):e015529.
84. Hamilton E, Friesen N. Online Education: A Science and Technology Studies Perspective. *Canadian Journal of Learning and Technology.* 2013;39(2).
85. Winters N, Oliver M, Langer L. Can mobile health training meet the challenge of 'measuring better'? *Comparitive Education.* 2017;53(1):115-131.
86. Rowe AK, de Savigny D, Lanata CF, Victora CG. How can we achieve and maintain high-quality performance of health workers in low-resource settings? *Lancet.* 2005;366(9490):1026-1035.
87. Greenhalgh T, Annandale E, Ashcroft R, et al. An open letter to The BMJ editors on qualitative research. *BMJ.* 2016;352:i563.
88. O'Malley G, Perdue T, Petracca F. A framework for outcome-level evaluation of in-service training of health care workers. *Human Resources for Health.* 2013;11.
89. Kok MC, Broerse JEW, Theobald S, Ormel H, Dieleman M, Taegtmeyer M. Performance of community health workers: situating their intermediary position within complex adaptive health systems. *Hum Resour Health.* 2017;15(1):59.

For peer review only - http://bmjopen.bmj.com/site/about/guidelines.xhtml

90. Bennett M. A Review of the Literature on the Benefits and Drawbacks of Participatory Action Research *First Peoples Child & Family Review.* 2004;1(1):19-32.
91. Perry H, Crigler L. *Developing and Strengthening Community Health Worker Programs at Scale* Washington D.C. : USAID and MCHIP (Maternal and Child Health Integrated Program);2014.
92. Grietens KP, Ribera JM, Erhart A, et al. Doctors and vampires in sub-Saharan Africa: ethical challenges in clinical trial research. *Am J Trop Med Hyg.* 2014;91(2):213-215.
93. Tricco AC, Lillie E, Zarin W, et al. A scoping review on the conduct and reporting of scoping reviews. *BMC Med Res Methodol.* 2016;16:15.

For peer review only

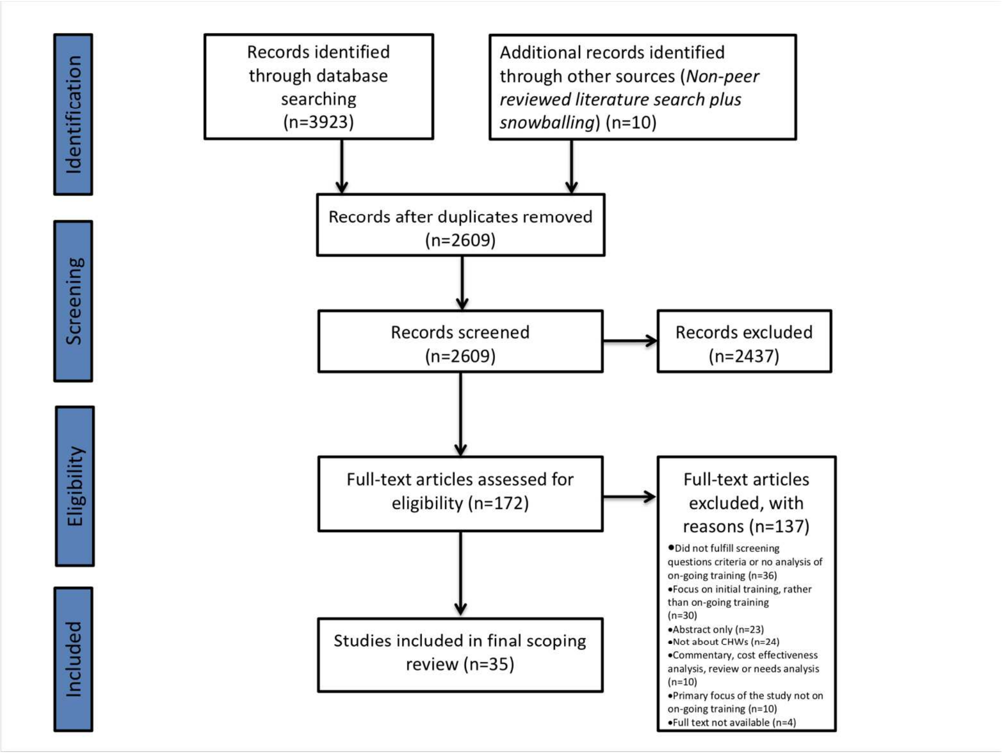

Figure 1. PRISMA flow diagram. The PRIMSA diagram details the search and selection process applied during the scoping review.

For peer review only

Supplementary Material

Table 1. Database search strategies, including search terms.

Database(s)	Search strategy
**AMED** (Allied and Complementary Medicine) 1985 to July 2017, **Embase** 1974 to 2017 July 10, **Global Health** 1973 to 2017 Week 29 **Ovid MEDLINE(R)** 1946 to July Week 2 2017.	**1.** Developing Countries**.sh,kf.**<br>**2.** (Africa or Asia or Caribbean or West Indies or South America or Latin America or Central America)**.hw,kf,ti,ab,cp.**<br>**3.** (Afghanistan or Albania or Algeria or Angola or Antigua or Barbuda or Argentina or Armenia or Armenian or Aruba or Azerbaijan or Bahrain or Bangladesh or Barbados or Benin or Byelarus or Byelorussian or Belarus or Belorussian or Belorussia or Belize or Bhutan or Bolivia or Bosnia or Herzegovina or Hercegovina or Botswana or Brasil or Brazil or Bulgaria or Burkina Faso or Burkina Fasso or Upper Volta or Burundi or Urundi or Cambodia or Khmer Republic or Kampuchea or Cameroon or Cameroons or Cameron or Camerons or Cape Verde or Central African Republic or Chad or Chile or China or Colombia or Comoros or Comoro Islands or Comores or Mayotte or Congo or Zaire or Costa Rica or Cote d'Ivoire or Ivory Coast or Croatia or Cuba or Cyprus or Czechoslovakia or Czech Republic or Slovakia or Slovak Republic or Djibouti or French Somaliland or Dominica or Dominican Republic or East Timor or East Timur or Timor Leste or Ecuador or Egypt or United Arab Republic or El Salvador or Eritrea or Estonia or Ethiopia or Fiji or Gabon or Gabonese Republic or Gambia or Gaza or Georgia Republic or Georgian Republic or Ghana or Gold Coast or Greece or Grenada or Guatemala or Guinea or Guam or Guiana or Guyana or Haiti or Honduras or Hungary or India or Maldives or Indonesia or Iran or Iraq or Isle of Man or Jamaica or Jordan or Kazakhstan or Kazakh or Kenya or Kiribati or Korea or Kosovo or Kyrgyzstan or Kirghizia or Kyrgyz Republic or Kirghiz or Kirgizstan or Lao PDR or Laos or Latvia or Lebanon or Lesotho or Basutoland or Liberia or Libya or Lithuania or Macedonia or Madagascar or Malagasy Republic or Malaysia or Malaya or Malay or Sabah or Sarawak or Malawi or Nyasaland or Mali or Malta or Marshall Islands or Mauritania or Mauritius or Agalega Islands or Mexico or Micronesia or Middle East or Moldova or Moldovia or Moldovian or Mongolia or Montenegro or Morocco or Ifni or Mozambique or Myanmar or Myanma or Burma or Namibia or Nepal or Netherlands Antilles or New Caledonia or Nicaragua or Niger or Nigeria or Northern Mariana Islands or Oman or Muscat or Pakistan or Palau or Palestine or Panama or Paraguay or Peru or Philippines or

For peer review only - http://bmjopen.bmj.com/site/about/guidelines.xhtml

	Philipines or Phillipines or Phillippines or Poland or Portugal or Puerto Rico or Romania or Rumania or Roumania or Russia or Russian or Rwanda or Ruanda or Saint Kitts or St Kitts or Nevis or Saint Lucia or St Lucia or Saint Vincent or St Vincent or Grenadines or Samoa or Samoan Islands or Navigator Island or Navigator Islands or Sao Tome or Saudi Arabia or Senegal or Serbia or Montenegro or Seychelles or Sierra Leone or Slovenia or Sri Lanka or Ceylon or Solomon Islands or Somalia or South Africa or Sudan or Suriname or Surinam or Swaziland or Syria or Tajikistan or Tadzhikistan or Tadjikistan or Tadzhik or Tanzania or Thailand or Togo or Togolese Republic or Tonga or Trinidad or Tobago or Tunisia or Turkey or Turkmenistan or Turkmen or Uganda or Ukraine or Uruguay or USSR or Soviet Union or Union of Soviet Socialist Republics or Uzbekistan or Uzbek or Vanuatu or New Hebrides or Venezuela or Vietnam or Viet Nam or West Bank or Yemen or Yugoslavia or Zambia or Zimbabwe or Rhodesia).**hw,kf,ti,ab,cp.**<br>**4.** ((developing or less* developed or under developed or underdeveloped or middle income or low* income or underserved or under served or deprived or poor*) adj (countr* or nation? or population? or world)).**ti,ab.**<br>**5.** ((developing or less* developed or under developed or underdeveloped or middle income or low* income) adj (economy or economies)).**ti,ab.**<br>**6.** (low* adj (gdp or gnp or gross domestic or gross national)).**ti,ab.**<br>**7.** (low adj3 middle adj3 countr*).**ti,ab.**<br>**8.** (lmic or lmics or third world or lami countr*).**ti,ab.**<br>**9.** transitional countr*.**ti,ab.**<br>**10.** or/1-9<br>AND<br>11. ((refresher* adj (train* or course*)) or (adequa* adj2 train*) or (on-going training) or (on-going education) or (continuing education) or ((in-service or update or recap*) adj3 train*)) or (exp education, continuing/ or exp inservice training/) or (supervision) or (supportive supervis*)<br>AND<br>12. ((community adj health* adj3 (worker* or volunteer or aide* or practition*)) or (community adj (mental health*) adj3 (worker* or volunteer or aide*)) or (village adj health* adj (worker* or team* or guide*)) or (lady health worker*) or (lady health visitor*) or (front-line primary health?care) or (front-line primary health care) or behvarz or brigadista or manzaneras or (rural health assistant*) or gramsakhi or (lay health worker*) or (trained birth assistant*) or (accredited social health activist*) or (adherence support worker*) or (care facilitator*) or (community adj10 (treatment support*)) or (community* adj4 (distributor* or volunteer*)) or (health extension worker*) or (lay counsellor*) or (maternal health

For peer review only

	worker*) or (shasthy? shebikas) or (shasthy? kormis) or (front line primary health* care worker) or (front line primary healthcare worker*) or (health activist*)).ti,ab.
**SCOPUS**	(TITLE-ABS-KEY ((front-line AND primary AND healthcare) OR (front-line AND primary AND health AND care) OR behvarz OR brigadista OR manzaneras OR (rural AND health AND assistant*) OR gramsakhi OR (lay AND health AND worker*) OR (trained AND birth AND assistant*) OR (accredited AND social AND health AND activist*) OR (adherence AND support AND worker*) OR (care AND facilitator*))) OR (TITLE-ABS-KEY ((community AND health* W/3 (worker* OR volunteer OR aide* OR practit*)) OR (community AND mental AND health* W/3 (worker* OR volunteer OR aide* OR practit*)) OR (village AND health* W/1 (worker* OR team* OR guide*)) OR (lady AND health AND worker*) OR (lady AND health AND visitor*) OR (lay AND healthworker) OR (TITLE-ABS-KEY ((health AND extension AND worker*) OR (lay AND counsellor*) OR (maternal AND health AND worker*) OR (peer AND educator*) OR (shasthy* AND shebikas) OR (shasthy* AND kormi) OR (front AND line AND primary AND health* AND care AND worker) OR (front AND line AND primary AND healthcare AND worker*) OR (health AND activist*))) OR (TITLE-ABS-KEY (community W/10 treatment AND support*)) OR (TITLE-ABS-KEY (community W/10 distributor*)) OR (TITLE-ABS-KEY (community W/10 volunteer*)) OR (TITLE-ABS-KEY (community W/10 care AND worker*)) OR (TITLE-ABS-KEY (community W/10 health AND worker*))<br><br>**AND**<br>(TITLE-ABS-KEY (refresher AND train* OR refresher AND course* OR (adequa* W/2 train*) OR (on-going education) OR (on-going training) OR (continuing education) OR (continuing training) OR (recap training) OR (in-service training) OR (update training) OR (supervision) or (supportive supervision)<br><br>**Limits applied**

For peer review only

	1. Countries - (Afghanistan or Albania or Algeria or Angola or Antigua or Barbuda or Argentina or Armenia or Armenian or Aruba or Azerbaijan or Bahrain or Bangladesh or Barbados or Benin or Byelarus or Byelorussian or Belarus or Belorussian or Belorussia or Belize or Bhutan or Bolivia or Bosnia or Herzegovina or Hercegovina or Botswana or Brasil or Brazil or Bulgaria or Burkina Faso or Burkina Fasso or Upper Volta or Burundi or Urundi or Cambodia or Khmer Republic or Kampuchea or Cameroon or Cameroons or Cameron or Camerons or Cape Verde or Central African Republic or Chad or Chile or China or Colombia or Comoros or Comoro Islands or Comores or Mayotte or Congo or Zaire or Costa Rica or Cote d'Ivoire or Ivory Coast or Croatia or Cuba or Cyprus or Czechoslovakia or Czech Republic or Slovakia or Slovak Republic or Djibouti or French Somaliland or Dominica or Dominican Republic or East Timor or East Timur or Timor Leste or Ecuador or Egypt or United Arab Republic or El Salvador or Eritrea or Estonia or Ethiopia or Fiji or Gabon or Gabonese Republic or Gambia or Gaza or Georgia Republic or Georgian Republic or Ghana or Gold Coast or Greece or Grenada or Guatemala or Guinea or Guam or Guiana or Guyana or Haiti or Honduras or Hungary or India or Maldives or Indonesia or Iran or Iraq or Isle of Man or Jamaica or Jordan or Kazakhstan or Kazakh or Kenya or Kiribati or Korea or Kosovo or Kyrgyzstan or Kirghizia or Kyrgyz Republic or Kirghiz or Kirgizstan or Lao PDR or Laos or Latvia or Lebanon or Lesotho or Basutoland or Liberia or Libya or Lithuania or Macedonia or Madagascar or Malagasy Republic or Malaysia or Malaya or Malay or Sabah or Sarawak or Malawi or Nyasaland or Mali or Malta or Marshall Islands or Mauritania or Mauritius or Agalega Islands or Mexico or Micronesia or Middle East or Moldova or Moldovia or Moldovian or Mongolia or Montenegro or Morocco or Ifni or Mozambique or Myanmar or Myanma or Burma or Namibia or Nepal or Netherlands Antilles or New Caledonia or Nicaragua or Niger or Nigeria or Northern Mariana Islands or Oman or Muscat or Pakistan or Palau or Palestine or Panama or Paraguay or Peru or Philippines or Philipines or Phillipines or Phillippines or Poland or Portugal or Puerto Rico or Romania or Rumania or Roumania or Russia or Russian or Rwanda or Ruanda or Saint Kitts or St Kitts or Nevis or Saint Lucia or St Lucia or Saint Vincent or St Vincent or Grenadines or Samoa or Samoan Islands

For peer review only

	or Navigator Island or Navigator Islands or Sao Tome or Saudi Arabia or Senegal or Serbia or Montenegro or Seychelles or Sierra Leone or Slovenia or Sri Lanka or Ceylon or Solomon Islands or Somalia or South Africa or Sudan or Suriname or Surinam or Swaziland or Syria or Tajikistan or Tadzhikistan or Tadjikistan or Tadzhik or Tanzania or Thailand or Togo or Togolese Republic or Tonga or Trinidad or Tobago or Tunisia or Turkey or Turkmenistan or Turkmen or Uganda or Ukraine or Uruguay or USSR or Soviet Union or Union of Soviet Socialist Republics or Uzbekistan or Uzbek or Vanuatu or New Hebrides or Venezuela or Vietnam or Viet Nam or West Bank or Yemen or Yugoslavia or Zambia or Zimbabwe or Rhodesia)<br>2. Dates - (1978-2017)

For peer review only

**Web of Science**	# 25 - #24 AND #16 AND #15<br>Indexes=SCI-EXPANDED, SSCI, A&HCI, CPCI-S, CPCI-SSH, BKCI-S, BKCI-SSH, ESCI, CCR-EXPANDED, IC Timespan=1978-2017<br># 24 - #23 OR #22 OR #21 OR #20 OR #19 OR #18 OR #17<br>Indexes=SCI-EXPANDED, SSCI, A&HCI, CPCI-S, CPCI-SSH, BKCI-S, BKCI-SSH, ESCI, CCR-EXPANDED, IC Timespan=1978-2017<br># 23 - TS=((health extension worker*) or (lay counsellor*) or (maternal health worker*) or (shasthy* kormis) or (shasthy* shebikas) or (health activist*))<br>Indexes=SCI-EXPANDED, SSCI, A&HCI, CPCI-S, CPCI-SSH, BKCI-S, BKCI-SSH, ESCI, CCR-EXPANDED, IC Timespan=1978-2017<br># 22 - TS=(community SAME treatment support*) OR TS=(community* NEAR/4 distributor*) OR TS=(community* NEAR/4 volunteer*) OR TS= (community* NEAR/4 care worker*) OR TS=(community* NEAR/4 careworker*) OR TS=(community* NEAR/4 healthworker*) OR TS=(community* NEAR/4 health worker*)<br>Indexes=SCI-EXPANDED, SSCI, A&HCI, CPCI-S, CPCI-SSH, BKCI-S, BKCI-SSH, ESCI, CCR-EXPANDED, IC Timespan=1978-2017<br># 21 - TS=((accredited social health activist*) or (adherence support worker*) or (care facilitator*))<br>Indexes=SCI-EXPANDED, SSCI, A&HCI, CPCI-S, CPCI-SSH, BKCI-S, BKCI-SSH, ESCI, CCR-EXPANDED, IC Timespan=1978-2017<br># 20 - TS=(behvarz or brigadista or manzaneras or (rural health assistant*) or gramsakhi or (lay health worker*) or (trained birth assistant*))<br>Indexes=SCI-EXPANDED, SSCI, A&HCI, CPCI-S, CPCI-SSH, BKCI-S, BKCI-SSH, ESCI, CCR-EXPANDED, IC Timespan=1978-2017<br># 19 - Ts=(frontline primary healthcare*) OR TS=(front line primary healthcare*) OR TS=(frontline primary health care*) OR TS=(front line primary health care*)<br>Indexes=SCI-EXPANDED, SSCI, A&HCI, CPCI-S, CPCI-SSH, BKCI-S, BKCI-SSH, ESCI, CCR-EXPANDED, IC Timespan=1978-2017<br># 18 - TS=(village health* worker*) OR TS=(village health* guide*) OR TS=(village health* team) OR TS=(lady health worker*)<br>Indexes=SCI-EXPANDED, SSCI, A&HCI, CPCI-S, CPCI-SSH, BKCI-S, BKCI-SSH, ESCI, CCR-EXPANDED, IC Timespan=1978-2017<br># 17 - TS=(community health* NEAR/3 worker*) OR TS=(community health* NEAR/3 volunteer*) OR TS=(community health* NEAR/3 aide*) OR TS=(community health* NEAR/3 practit*) OR TS=(community mental health* NEAR/3 worker*) OR TS=(community mental health* NEAR/3 volunteer*) OR TS=(community mental health* NEAR/3 aide*)<br>Indexes=SCI-EXPANDED, SSCI, A&HCI, CPCI-S, CPCI-SSH, BKCI-S, BKCI-SSH, ESCI, CCR-EXPANDED, IC Timespan=1978-2017

For peer review only - http://bmjopen.bmj.com/site/about/guidelines.xhtml

	# 16 98,552<br>#14 OR #13 OR #12<br>Indexes=SCI-EXPANDED, SSCI, A&HCI, CPCI-S, CPCI-SSH, BKCI-S, BKCI-SSH, ESCI, CCR-EXPANDED, IC<br>Timespan=1978-2017<br># 15 3,687,339<br>#11 OR #10 OR #9 OR #8 OR #7 OR #6 OR #5 OR #4 OR #3 OR #2 OR #1<br>Indexes=SCI-EXPANDED, SSCI, A&HCI, CPCI-S, CPCI-SSH, BKCI-S, BKCI-SSH, ESCI, CCR-EXPANDED, IC<br>Timespan=1978-2017<br># 14 42,703<br>TS=(recap* NEAR/3 train*) OR TS=(update NEAR/3 train*) OR TS=(ongoing NEAR/3 train*) OR TS=(in-service NEAR/3 train*) OR TS=(on-going education) OR TS=(supervision) OR TS=(supportive supervision)<br>Indexes=SCI-EXPANDED, SSCI, A&HCI, CPCI-S, CPCI-SSH, BKCI-S, BKCI-SSH, ESCI, CCR-EXPANDED, IC<br>Timespan=1978-2017<br># 13 53,039<br>TS=(continuing education) OR TS=(continuing training)<br>Indexes=SCI-EXPANDED, SSCI, A&HCI, CPCI-S, CPCI-SSH, BKCI-S, BKCI-SSH, ESCI, CCR-EXPANDED, IC<br>Timespan=1978-2017<br># 12 4,506<br>TS=(refresher train* or refresher course*) OR TS=(adequa* NEAR/2 train*)<br>Indexes=SCI-EXPANDED, SSCI, A&HCI, CPCI-S, CPCI-SSH, BKCI-S, BKCI-SSH, ESCI, CCR-EXPANDED, IC<br>Timespan=1978-2017<br># 11 141,978<br>TS=(underserved population* or underserved world* or underserved countr* or underserved nation*) OR TS=(under served population* or under served world* or under served countr* or under served nation*) OR TS=(deprived population* or deprived world* or deprived countr* or deprived nation*) OR TS=(poor population* or poor world* or poorcountr* or poor nation*)<br>Indexes=SCI-EXPANDED, SSCI, A&HCI, CPCI-S, CPCI-SSH, BKCI-S, BKCI-SSH, ESCI, CCR-EXPANDED, IC<br>Timespan=1978-2017<br># 10 438,437<br>TS=(developing population* or developing world*) OR TS=(less developed population* or less developed world*) OR TS=(under developed population* or under developed world*) OR TS=(underdeveloped population* or underdeveloped world*) OR TS=(middle income population* or middle income world*) OR TS=(low* income population* or low* income world*)<br>Indexes=SCI-EXPANDED, SSCI, A&HCI, CPCI-S, CPCI-SSH, BKCI-S, BKCI-SSH, ESCI, CCR-EXPANDED, IC<br>Timespan=1978-2017<br># 9 354,673<br>TS=(developing countr* or developing nation*) OR TS=(less developed countr* or less developed nation*) OR TS=(under developed countr* or under developed nation*) OR

For peer review only - http://bmjopen.bmj.com/site/about/guidelines.xhtml

	TS=(underdeveloped countr* or underdeveloped nation*) OR TS=(middle income countr* or middle income nation*) OR TS=(low* income countr* or low* income nation*)<br>Indexes=SCI-EXPANDED, SSCI, A&HCI, CPCI-S, CPCI-SSH, BKCI-S, BKCI-SSH, ESCI, CCR-EXPANDED, IC<br>Timespan=1978-2017<br># 8 50,977<br>TS=(developing economies or developing economy) OR TS=(less developed economies or less developed economy) OR TS=(under developed economies or under developed economy) OR TS=(underdeveloped economies or underdeveloped economy) OR TS=(middle income economies or middle income economies) OR TS=(low* income economies or low* income economy)<br>Indexes=SCI-EXPANDED, SSCI, A&HCI, CPCI-S, CPCI-SSH, BKCI-S, BKCI-SSH, ESCI, CCR-EXPANDED, IC<br>Timespan=1978-2017<br># 7 7,542<br>TS=(low* gdp or low* GNP or low* gross domestic or low* gross national)<br>Indexes=SCI-EXPANDED, SSCI, A&HCI, CPCI-S, CPCI-SSH, BKCI-S, BKCI-SSH, ESCI, CCR-EXPANDED, IC<br>Timespan=1978-2017<br># 6 15,495<br>TS=(low SAME middle SAME countr*)<br>Indexes=SCI-EXPANDED, SSCI, A&HCI, CPCI-S, CPCI-SSH, BKCI-S, BKCI-SSH, ESCI, CCR-EXPANDED, IC<br>Timespan=1978-2017<br># 5 32,915<br>TS= (lmic or lmics or third world or lami countr*)<br>Indexes=SCI-EXPANDED, SSCI, A&HCI, CPCI-S, CPCI-SSH, BKCI-S, BKCI-SSH, ESCI, CCR-EXPANDED, IC<br>Timespan=1978-2017<br># 4 2,471<br>TS=(transitional countr*)<br>Indexes=SCI-EXPANDED, SSCI, A&HCI, CPCI-S, CPCI-SSH, BKCI-S, BKCI-SSH, ESCI, CCR-EXPANDED, IC<br>Timespan=1978-2017<br># 2 522,425<br>TS=(Africa or Asia or Caribbean or West Indies or South America or Latin America or Central America)<br>Indexes=SCI-EXPANDED, SSCI, A&HCI, CPCI-S, CPCI-SSH, BKCI-S, BKCI-SSH, ESCI, CCR-EXPANDED, IC<br>Timespan=1978-2017<br># 1 213,604<br>TS=Developing countries<br>Indexes=SCI-EXPANDED, SSCI, A&HCI, CPCI-S, CPCI-SSH, BKCI-S, BKCI-SSH, ESCI, CCR-EXPANDED, IC<br>Timespan=1978-2017

For peer review only

**ASSIA**	ab(((community health* worker* or community health volunteer or or community health aide* or community health practitioner or community mental health* worker* or community mental health* volunteer or community mental health* aide* or village health* worker* or village health* team* or village health* guide* or lady health worker* or lady health visitor* or laywomen* or laywoman* or front-line primary healthcare or front-line primary health care or behvarz or brigadista or manzaneras or rural health assistant* or gramsakhi or lay health worker* or trained birth assistant* or accredited social health activist* or adherence support worker* or care facilitator* or (community and (treatment support*)) or (community* and (distributor* or volunteer* or care worker* or health worker)) or health extension worker* or lay counsellor* or maternal health worker* or peer educator* or shasthya shebikas or shasthya kormis or front line primary health* care worker or front line primary healthcare worker* or health activist*) AND (refresher* train* or refresher* course* or (adequa* and train*) or (continuing medical education) or (continuing medical training) or (continuing nursing education) or (continuing nursing training) or or (supervision) or (supportive supervision) or (continuing education) or ((in-service or ongoing or update or recap*) and train*)) AND (developing countries or Africa or Asia or Caribbean or West Indies or South America or Latin America or Central America or lmic or underdeveloped countries or middle income countries or low income countries or transitional countries))) OR ti((community health* worker* OR community health volunteer OR community health aide* OR community mental health* worker* OR community mental health* volunteer OR community mental health* aide* OR village health* worker* OR village health* team* OR village health* guide* OR lady health worker* OR lady health visitor* OR laywomen* OR laywoman* OR front-line primary healthcare OR front-line primary health care OR behvarz OR brigadista OR manzaneras OR rural health assistant* OR gramsakhi OR lay health worker* OR trained birth assistant* OR accredited social health activist* OR adherence support worker* OR care facilitator* OR (community AND (treatment support*)) OR (community* AND (distributor* OR volunteer* OR care worker* OR health worker)) OR health extension worker* OR lay counsellor* OR maternal health worker* OR peer educator* OR shasthy* shebikas OR shasthy* kormis OR front line primary health* care worker OR front line primary healthcare worker* OR health activist*) AND (refresher* train* OR refresher* course* OR (adequa* AND train*) OR (continuing medical education) OR (continuing medical training) OR (continuing nursing education) OR (continuing

	nursing training) OR (continuing education) OR ((in-service OR ongoing OR update OR recap*) AND train*)) AND (developing countries OR Africa OR Asia OR Caribbean OR West Indies OR South America OR Latin America OR Central America OR lmic OR underdeveloped countries OR middle income countries OR low income countries OR transitional countries))
**LILACS**	community health workers 7<br>community health volunteer 0<br>community health aide 2<br>community mental health worker 0<br>community mental health volunteer 0<br>community mental health aide or 0<br>village health worker 1<br>village health team 0<br>village health guide 0<br>lady health worker 0<br>lady health visitor 0<br>laywomen 0<br>laywoman 0<br>front-line primary healthcare 0<br>front-line primary health care 0<br>behvarz 0<br>brigadista 0<br>manzaneras or<br>rural health assistant* 0<br>gramsakhi 0<br>lay health worker 0<br>trained birth assistant 0<br>accredited social health activist 0<br>adherence support worker 0<br>care facilitator 0<br>treatment support 11<br>community 0<br>health extension worker 0<br>lay counsellor 0<br>maternal health worker 0<br>peer educator 0<br>shasthya shebikas 0<br>shasthya kormis 0<br>health activist 0<br><br>and<br><br>refresher training or refresher course or adequate training or adequately trained or on-going education or on-going training or continuing education or in-service training or update training or recap or supervision or supportive

For peer review only - http://bmjopen.bmj.com/site/about/guidelines.xhtml

	supervision
**British Educational Index, ERIC via EBSCO**	S13 S10 AND S11 AND S12<br>S12 TI (((community N1 health* N3 (worker* or volunteer or aide* or practition*)) or (community N1 (mental health* N3 (worker* or volunteer or aide*)) or (village N1 health* N1 (worker* or team* or guide*)) or (lady health worker*) or (lady health visitor*) or (laywomen* or laywoman*) or (front-line primary healthcare) or (front-line primary health care) or behvarz or brigadista or manzaneras or (rural health assistant*) or gramsakhi or (lay health worker*) or (trained birth assistant*) or (accredited social health activist*) or (adherence support worker*) or (care facilitator*) or (community N10 (treatment support*)) or (community* N4 (distributor* or volunteer* or (care worker*) or (health worker))) or (health extension worker*) or (lay counsellor*) or (maternal health worker*) or (peer educator*) or (shasthya shebikas) or (shasthya kormis) or (front line primary health* care worker) or (front line primary healthcare worker*) or (health activist*))) OR AB (((community N1 health* N3 (worker* or volunteer or aide*)) or (community N1 (mental health* N3 (worker* or volunteer or aide*)) or (village N1 health* N1 (worker* or team* or guide*)) or (lady health worker*) or (lady health visitor*) or (laywomen* or laywoman*) or (front-line primary healthcare) or (front-line primary health care) or behvarz or brigadista or manzaneras or (rural health assistant*) or gramsakhi or (lay health worker*) or (trained birth assistant*) or (accredited social health activist*) or (adherence support worker*) or (care facilitator*) or (community N10 (treatment support*)) or (community* N4 (distributor* or volunteer* or (care worker*) or (health worker))) or (health extension worker*) or (lay counsellor*) or (maternal health worker*) or (shasthy* shebikas) or (shasthy* korbis) or (front line primary health* care worker) or (front line primary healthcare worker*) or (health activist*)))<br>S11 TI (((refresher* N1 (train* or course*)) or (adequa* N2 train*) or (on?going education) or (on?going training) or (continuing education) or (supervision) or (supportive supervision) or ((in-service or ongoing or update or recap*) N3 train*))) OR AB (((refresher* N1 (train* or course*)) or (adequa* N2 train*) or (on?going education) or (on?going training) or (supervision) or (supportive supervis*) or ((in-service or ongoing or update or recap*) N3 train*)))<br>S10 S1 OR S2 OR S3 OR S4 OR S5 OR S6 OR S7 OR S8 OR S9<br>S9 TI transitional countr* OR AB transitional countr*<br>S8 TI (lmic or lmics or third world or lami countr*)) OR AB (lmic or lmics or third world or lami countr*))<br>S7 TI (low N3 middle N3 countr*) OR AB (low N3 middle N3 countr*)<br>S6 TI ((low* N1 (gdp or gnp or gross domestic or gross national))) OR AB ((low* N1 (gdp or gnp or gross domestic or gross national)))<br>S5 TI (((developing or less* developed or under developed or underdeveloped or middle income or low* income) N1 (economy

For peer review only - http://bmjopen.bmj.com/site/about/guidelines.xhtml

	or economies))) OR AB (((developing or less* developed or under developed or underdeveloped or middle income or low* income) N1 (economy or economies)))<br>S4 TI (((developing or less* developed or under developed or underdeveloped or middle income or low* income or underserved or under served or deprived or poor*) N1 (countr* or nation* or population* or world))) OR AB (((developing or less* developed or under developed or underdeveloped or middle income or low* income or underserved or under served or deprived or poor*) N1 (countr* or nation* or population* or world)))<br>S3 TI (Afghanistan or Albania or Algeria or Angola or Antigua or Barbuda or Argentina or Armenia or Armenian or Aruba or Azerbaijan or Bahrain or Bangladesh or Barbados or Benin or Byelarus or Byelorussian or Belarus or Belorussian or Belorussia or Belize or Bhutan or Bolivia or Bosnia or Herzegovina or Hercegovina or Botswana or Brasil or Brazil or Bulgaria or Burkina Faso or Burkina Fasso or Upper Volta or Burundi or Urundi or Cambodia or Khmer Republic or Kampuchea or Cameroon or Cameroons or Cameron or Camerons or Cape Verde or Central African Republic or Chad or Chile or China or Colombia or Comoros or Comoro Islands or Comores or Mayotte or Congo or Zaire or Costa Rica or Cote d'Ivoire or Ivory Coast or Croatia or Cuba or Cyprus or Czechoslovakia or Czech Republic or Slovakia or Slovak Republic or Djibouti or French Somaliland or Dominica or Dominican Republic or East Timor or East Timur or Timor Leste or Ecuador or Egypt or United Arab Republic or El Salvador or Eritrea or Estonia or Ethiopia or Fiji or Gabon or Gabonese Republic or Gambia or Gaza or Georgia Republic or Georgian Republic or Ghana or Gold Coast or Greece or Grenada or Guatemala or Guinea or Guam or Guiana or Guyana or Haiti or Honduras or Hungary or India or Maldives or Indonesia or Iran or Iraq or Isle of Man or Jamaica or Jordan or Kazakhstan or Kazakh or Kenya or Kiribati or Korea or Kosovo or Kyrgyzstan or Kirghizia or Kyrgyz Republic or Kirghiz or Kirgizstan or Lao PDR or Laos or Latvia or Lebanon or Lesotho or Basutoland or Liberia or Libya or Lithuania or Macedonia or Madagascar or Malagasy Republic or Malaysia or Malaya or Malay or Sabah or Sarawak or Malawi or Nyasaland or Mali or Malta or Marshall Islands or Mauritania or Mauritius or Agalega Islands or Mexico or Micronesia or Middle East or Moldova or Moldovia or Moldovian or Mongolia or Montenegro or Morocco or Ifni or Mozambique or Myanmar or Myanma or Burma or Namibia or Nepal or Netherlands Antilles or New Caledonia or Nicaragua or Niger or Nigeria or Northern Mariana Islands or Oman or Muscat or Pakistan or Palau or Palestine or Panama or Paraguay or Peru or Philippines or Philipines or Phillipines or Phillippines or Poland or Portugal or Puerto Rico or Romania or Rumania or Roumania or Russia or Russian or Rwanda or Ruanda or Saint Kitts or St Kitts or Nevis or Saint Lucia or St Lucia or Saint Vincent or St Vincent or Grenadines or Samoa or Samoan Islands or Navigator Island or Navigator Islands or Sao Tome or Saudi Arabia or Senegal or Serbia or Montenegro or Seychelles or Sierra Leone or Slovenia or Sri Lanka or Ceylon or Solomon Islands or Somalia or South Africa or Sudan or Suriname or Surinam or Swaziland or Syria or Tajikistan or Tadzhikistan or Tadjikistan or Tadzhik or Tanzania

For peer review only

	or Thailand or Togo or Togolese Republic or Tonga or Trinidad or Tobago or Tunisia or Turkey or Turkmenistan or Turkmen or Uganda or Ukraine or Uruguay or USSR or Soviet Union or Union of Soviet Socialist Republics or Uzbekistan or Uzbek or Vanuatu or New Hebrides or Venezuela or Vietnam or Viet Nam or West Bank or Yemen or Yugoslavia or Zambia or Zimbabwe or Rhodesia) OR AB (Afghanistan or Albania or Algeria or Angola or Antigua or Barbuda or Argentina or Armenia or Armenian or Aruba or Azerbaijan or Bahrain or Bangladesh or Barbados or Benin or Byelarus or Byelorussian or Belarus or Belorussian or Belorussia or Belize or Bhutan or Bolivia or Bosnia or Herzegovina or Hercegovina or Botswana or Brasil or Brazil or Bulgaria or Burkina Faso or Burkina Fasso or Upper Volta or Burundi or Urundi or Cambodia or Khmer Republic or Kampuchea or Cameroon or Cameroons or Cameron or Camerons or Cape Verde or Central African Republic or Chad or Chile or China or Colombia or Comoros or Comoro Islands or Comores or Mayotte or Congo or Zaire or Costa Rica or Cote d'Ivoire or Ivory Coast or Croatia or Cuba or Cyprus or Czechoslovakia or Czech Republic or Slovakia or Slovak Republic or Djibouti or French Somaliland or Dominica or Dominican Republic or East Timor or East Timur or Timor Leste or Ecuador or Egypt or United Arab Republic or El Salvador or Eritrea or Estonia or Ethiopia or Fiji or Gabon or Gabonese Republic or Gambia or Gaza or Georgia Republic or Georgian Republic or Ghana or Gold Coast or Greece or Grenada or Guatemala or Guinea or Guam or Guiana or Guyana or Haiti or Honduras or Hungary or India or Maldives or Indonesia or Iran or Iraq or Isle of Man or Jamaica or Jordan or Kazakhstan or Kazakh or Kenya or Kiribati or Korea or Kosovo or Kyrgyzstan or Kirghizia or Kyrgyz Republic or Kirghiz or Kirgizstan or Lao PDR or Laos or Latvia or Lebanon or Lesotho or Basutoland or Liberia or Libya or Lithuania or Macedonia or Madagascar or Malagasy Republic or Malaysia or Malaya or Malay or Sabah or Sarawak or Malawi or Nyasaland or Mali or Malta or Marshall Islands or Mauritania or Mauritius or Agalega Islands or Mexico or Micronesia or Middle East or Moldova or Moldovia or Moldovian or Mongolia or Montenegro or Morocco or Ifni or Mozambique or Myanmar or Myanma or Burma or Namibia or Nepal or Netherlands Antilles or New Caledonia or Nicaragua or Niger or Nigeria or Northern Mariana Islands or Oman or Muscat or Pakistan or Palau or Palestine or Panama or Paraguay or Peru or Philippines or Philipines or Phillipines or Phillippines or Poland or Portugal or Puerto Rico or Romania or Rumania or Roumania or Russia or Russian or Rwanda or Ruanda or Saint Kitts or St Kitts or Nevis or Saint Lucia or St Lucia or Saint Vincent or St Vincent or Grenadines or Samoa or Samoan Islands or Navigator Island or Navigator Islands or Sao Tome or Saudi Arabia or Senegal or Serbia or Montenegro or Seychelles or Sierra Leone or Slovenia or Sri Lanka or Ceylon or Solomon Islands or Somalia or South Africa or Sudan or Suriname or Surinam or Swaziland or Syria or Tajikistan or Tadzhikistan or Tadjikistan or Tadzhik or Tanzania or Thailand or Togo or Togolese Republic or Tonga or Trinidad or Tobago or Tunisia or Turkey or Turkmenistan or Turkmen or Uganda or Ukraine or Uruguay or USSR or Soviet Union or Union of Soviet Socialist Republics or Uzbekistan or Uzbek or Vanuatu

For peer review only

	or New Hebrides or Venezuela or Vietnam or Viet Nam or West Bank or Yemen or Yugoslavia or Zambia or Zimbabwe or Rhodesia)<br>S2 SU (Africa or Asia or Caribbean or West Indies or South America or Latin America or Central America) OR MW (Africa or Asia or Caribbean or West Indies or South America or Latin America or Central America) OR MM (Africa or Asia or Caribbean or West Indies or South America or Latin America or Central America) OR MJ (Africa or Asia or Caribbean or West Indies or South America or Latin America or Central America) OR TI (Africa or Asia or Caribbean or West Indies or South America or Latin America or Central America) OR AB (Africa or Asia or Caribbean or West Indies or South America or Latin America or Central America)<br>S1 SU Developing Countries OR MW Developing Countries

For peer review only

For peer review only - http://bmjopen.bmj.com/site/about/guidelines.xhtml

Table 2. Results from individual database searches.

Database	Number of hits	Page number for search strategy details
Medline (via OVID)	391	2-4
EMBASE (via OVID)	505	2-4
Global Health (via OVID)	351	2-4
AMED (via OVID)	36	2-4
Scopus	1217	5-6
Web of Science	1001	7-9
ASSIA via ProQuest	64	10
LILACS	21	11-12
BEI via EBSCO	38	13-16
ERIC via EBSCO	262	13-16
CINAHL	67	13-16

For peer review only

Legend. The results from individual database searches, including the number of hits and the supplementary material page numbers where the search strategies can be found.

Table 3. Details of included studies.

Authors	Study Title	Year	Country and Region	CHW name	Cadre description	Number of CHWs	Disease Focus Area	Training details	Outcome measure and outcomes	Use of mHealth
Adejumo et al.[34]	Community referral for presumptive TB in Nigeria: a comparison of four models of active case finding.	2016	Nigeria, West Africa	Community Workers (CWs)	Cadre description varied depending on the district from:<br><br>A) Unsupervised volunteer CWs where selection criteria was "any interested member of the community". Paid $13-20 quarterly.<br><br>to<br><br>B) 'Direct dealing CWs' whose selection criteria was that they had to:<br>1. Be known to the local leader;	124	TB	**Type:** Supervision<br>**Content:** No details<br>**Duration:** Variable number of supervisory visits, ranging from no supervision to three monthly depending on the model of supervision.<br>**Provider:** TB Local Government Supervisors and community based partner organisations<br>**Location:** Varying from no supervision to monthly meetings in the office and	**Outcome measure(s):** Change in behaviour, attitude or practice e.g. number of cases of TB detected in the community.<br><br>**Outcome(s):** The highest median referrals and mean TB diagnoses was obtained by the model with training supervision, and $80/quarterly payments (Comprehensive Quotas-Oriented model). The model with irregularly supervised, trained, and compensated	No details

For peer review only - http://bmjopen.bmj.com/site/about/guidelines.xhtml

					community;<br>4. Preferably have previous active involvement in volunteer work.<br>Paid $80 quarterly.					
Ameha et al.[35]	Effectivenes s of supportive supervision on the consistency of integrated community cases management skills of the health extension workers in 113 districts of Ethiopia.	2013	Ethiopia, East Africa	Health Extension Workers (HEWs)	No details provided	5000	Child Health	**Type:** Supervision<br>**Content:** Review of at least two cases from register and performance coaching<br>**Duration:** Variable number of supervisory visits -minimum of one, maximum of four.<br>**Provider:** John Snow, Inc. through the Last Ten Kilometers project (L10K) in partnership with the Ministry of Health<br>**Location:** Health posts	**Outcome measure(s):** Change in behaviour, attitude or practice e.g. number of recorded cases of diahorrea, malaria and pneumonia managed correctly in the community<br><br>**Outcome(s):** After controlling for secular trend and other factors, significant dose-response relationships were observed between number of supportive supervision visits and Integrated Community Case Management (iCCM) treatment indictors	No details
Ayele et al.[36]	The functional status of community	1993	Ethiopia, East Africa	Community Health Agents (CHAs)	No details provided	102	General focus	**Type:** Refresher training course and supervision.<br>**Content:** No	**Outcome measure(s):** Change in behaviour, attitude	No details

For peer review only

	health agents: A trial of refresher courses and regular supervision.							details<br>**Duration:** Five-day refresher course and one supervision per month.<br>**Provider:** Community leaders<br>**Location:** In the community (field supervision)	or practice e.g. number of home visits, registration activities<br><br>**Outcome(s):** 10 out of the 13 CHA activity scores were higher in the group receiving refresher training and supervision at 3 and 6 months compared to the group not receiving it	
Carlough & McCall[37]	Skilled birth attendance: What does it mean and how can it be measured? A clinical skills assessment of maternal and child health workers in Nepal.	2005	Nepal, South Asia	Maternal and Child Health Workers (MCHWs)	Local women aged 18—35 who have completed a 15-week course in maternal and child health which covers both theoretical and practical components	104 (66 received refresher training)	Maternal and Reproductive Health	**Type:** Refresher training course.<br>**Content:** Midwifery and emergency obstetric skills including focused antenatal care, active management of the third stage of labor, initial care for postpartum hemorrhage, pre-eclampsia and infection, and immediate neonatal care. MCHWs who completed refresher training receiving a first aid obstetric	**Outcome measure(s):** Mixed methods. Knowledge and skills assessment using a Clinical Skills Assessment (CSA) tool, plus a qualitative self-assessment scale.<br><br>**Outcome(s):** The MCHWs who received refresher training performed significantly better than those who did not on the CSA especially in the domains of: Use of medications in pregnancy; Managing post-partum	No details

								emergency kit **Duration:** Six-week refresher training course. **Provider:** No details **Location:** No details	hemorrhage; Normal delivery management	
Das et al.[38]	Strengthening malaria service delivery through supportive supervision and community mobilization in an endemic Indian setting: an evaluation of nested delivery models	2015	India, South Asia	Accredited social health activists (ASHAs)	The ASHAs role is in the "early detection, management and prevention of malaria at the community level They have been trained to test for malaria cases using rapid diagnostic tests and to treat these cases with artemisinin combination therapy.	N/A (randomised at village level)	Infectious disease	**Type:** Supervision **Content:** Recapping knowledge about transmission, diagnosis and treatment of malaria; practical support for performing and interpreting rapid diagnosis tests; administration of the correct dosage of ACT and follow-up to monitor compliance and record keeping **Duration:** Twice monthly. **Provider:** A mixture between the governments National Vector Borne Disease Control Programme and an NGO **Location:** In the	**Outcome measure(s):** Change in behaviour, attitude or practice at the household level e.g. Assessing for increased use of long last insecticide treated bed nets and proportion of cases tested for falciparum malaria within 24 hours. **Outcome(s):** Combining supportive supervision of CHWs with community mobilisation resulted in greater usage of bed nets and greater likelihood to seek treatment from a CHW resulting in fever cases being more likely to receive a timely	No details

								field	diagnosis	
Datiko et al.[39]	Exploring providers' perspectives of a community based TB approach in Southern Ethiopia: implication for community based approaches.	2015	Ethiopia, East Africa	Health Extension Workers (HEWs)/Community Health Promoters (CHPs)	HEWs trained for 1 year; salaried members of formal health system; range of duties. In this project their role was to collect sputum, produce smears and support patient treatment.<br><br>CHPs were unpaid volunteers, selected by communities to play a support role to HEWs. Their role was to support the HEW in identifying TB cases.	20	TB	**Type:** Supervision<br>**Content:** Supervising HEWs practically and ensuring smooth running of the project e.g. collection of sputum, case identification etc.<br>**Duration:** Twice monthly<br>**Provider:** District field supervisors funded by TB Reach (a multilateral funding organisation)<br>**Location:** In the field	**Outcome measure(s):** Qualitative assessment. Interviews and focus discussion groups to elicit the experiences of providers.<br><br>**Outcome(s):** HEWs felt generally well supported by their supervisors.<br>A small number of HEWs referred to supervisors who had not fulfilled the demanding co-ordination role well.<br>The greatest challenge for supervisors was the intensity of their workload coupled with the need to cover the large geographical area of their district.	No details
Dewing et al.[40]	Lay Counselors' Ability to Deliver	2013	South Africa, Southern Africa	Lay Counselors (LCs)	Usually women carrying out functions related to health care	39	HIV	**Type:** Refresher training course and supportive	**Outcome measure(s):** Knowledge and skills assessment.	No details

	Counseling for Behavior Change.				delivery but who have no formal professional or paraprofessional certificate or degreed tertiary education. Their role specific to HIV relates to enhancing treatment adherence and encouraging safer sexual practice.			supervision. **Content:** Refresher training was aimed at covering difficulties counselors were experiencing with the eight-step 'Options' protocol and its delivery in the clinic setting. Supportive supervision recapped the protocol and MI principles. Learning was facilitated by means of demonstration, group discussion, role-plays, and self-evaluation. **Duration:**14 hours of refresher training over a two-day period and four 1-hour supportive supervision courses over a period of 4-months. **Provider:** Two counseling psychologists with experience in training lay	Lay counselors ability in motivational interviewing was assessed following refresher training using the Motivational Interviewing Treatment Integrity Tool and an instrument developed by the researchers.<br><br>**Outcome(s):** Although LCs did not achieve complete proficiency in MI, refresher training and supervision improved LCs basic counseling communication skills and therapeutic approach	

For peer review only

								counselors from the Western Cape Provincial Department of Health provided the refresher training course and individual NGOs provided the supervision. **Location:** No details		
Gallo et al.[41]	Evaluation of a volunteer community-based health worker programme for providing contraceptive services in Madagascar.	2013	Madagascar, East Africa	Community Health Workers (CHWs)	CHWs deliver maternal, reproductive health and family planning services.<br><br>Receive an initial 10-day training. Unpaid but can receive a small profit from commercial goods they sell e.g. condoms, oral and injectable contraception	100	Maternal and Reproductive Health	**Type:** Refresher training course. **Content:** No details **Duration:** Two-day refresher training course for those who did not meet the minimum level of competency following initial training. **Provider:** No details **Location:** No details	**Outcome measure(s):** Knowledge and skills assessment. A test involving five stimulated encounters regarding knowledge of an injectable contraception<br><br>**Outcome(s):** Refresher training resulted in higher scores on the clinical test, which consisted of assessing knowledge of injectable contraception, and five observed simulated client encounters.	No details
Gupta et al.[42]	Implementation of ORT:	1994	India, South Asia	Community Health	CHGs in this study were	323	Child Health	**Type:** Refresher training	**Outcome measure(s):**	No details

For peer review only - http://bmjopen.bmj.com/site/about/guidelines.xhtml

	some problems encountered in training of health workers during an operational research programme.			Guides (CHGs)/Anganwadi Health Workers (AHWs)	grassroots level, part time volunteers selected by village leaders from amongst the local residents. They receive 3 months of pre-service training and serve approximately 1000 people. Their role is to deliver primary healthcare in the village.<br><br>AHWs are also part time workers, with one AHW per village. They were trained for 3 months and their primary role was to deliver nutrition and healthcare services to children.<br><br>The government has overall responsibility for the workers.			course.<br>**Content:** Refresher course in Bengali recapping knowledge and containing important practical skills such as how to prepare ORS solution<br>**Duration:** One-day interactive refresher training course.<br>**Provider:** No details<br>**Location:** No details	Knowledge and skills assessment. Testing for improved knowledge of ORS using role playing and discussions.<br><br>**Outcome(s):** Following refresher training knowledge and skills on features such as use of home fluids, preparation of ORS and dosage of ORS was increased.	
Gupta et al.[43]	Improving quality of home-based postnatal	2016	India, South Asia	Auxiliary Nurse Midwives (ANMs)	ANMs receive 8 days of training in 'Integrated Management of	12	Child Health	**Type:** In-service training.<br>**Content:** microteaching to	**Outcome measure(s):** Knowledge and skills assessment.	No details

For peer review only

	care by microteaching of multipurpose workers in rural and urban slum areas of Chandigarh, India: a pilot study.				Newborn and Childhood Illnesses' and undertake 'skill based work'<br><br>No further details on remuneration or level of education.			enhance the postnatal care skills of ANMs<br>**Duration:** One 90-minute session every three months.<br>**Provider:** A Lady Health Visitor (LHV) and a male social worker<br>**Location:** A health post of the Dept. of Community Medicine, School of Public Health, Chandigarh.	Scores achieved on a structured checklist with items regarding maternal history and exam taking technique, new-born examination and maternal counselling.<br><br>**Outcome(s):** Maternal examination, maternal counseling regarding danger signs and newborn examination all improved significantly after the third round of microteaching. In addition more ANMs carried weighing scales, thermometers, and registers after receiving training.	
Hadi.[44]	Management of acute repiratory infections by community health volunteers: experience of Bangladesh	2003	Bangladesh, South Asia	Community Health Volunteers (CHVs)	The role of CHVs was to detect and treat cases of acute respiratory infection & to refer severe and complicated cases to nearby health clinics.	120	Child Health	**Type:** Supervision<br>**Content:** No details<br>**Duration:** Once a month.<br>**Provider:** Paramedics from BRAC<br>**Location:** No	**Outcome measure(s):** Knowledge and skills assessment. Comparing the diagnosis of acute respiratory infection and management between CHWs and	No details

	Rural Advancement Committee (BRAC).				Unpaid, selected from among the local area: most had only 5 years of schooling.<br><br>3 days of basic training covering theoretical and practical concepts of acute respiratory tract infections.			details	trained assessors.<br><br>**Outcome(s):** The "sensitivity, specificity, and overall agreement rates in diagnosing and treating ARIs" were significantly higher among the CHVs who were supervised.	
Horwood et al.[45]	A continuous quality improvement intervention to improve the effectiveness of community health workers providing care to mothers and children: a cluster randomised controlled trial in South Africa	2017	South Africa, Southern Africa	Community Health Workers (CHWs)	CHWs are recruited and deployed by the Department of Health and receive a small stipend.<br><br>They fulfill a variety of roles in the community including home-based care, education on prevention of mother to child transmission of HIV, adherence support for antiretroviral and TB treatment, and provision of maternal and child health services using	120	Maternal and Child Health.	**Type:** Supervision **Content:** Sessions focused on areas for improvement which were jointly decided with CHWs and supervisors **Duration:** Twice monthly. **Provider:** Mentors based at the University of KwaZulu-Natal **Location:** No details	**Outcome measure(s):** Mixed methods. Knowledge and skills were assessed using four questions, which were asked to mothers served by the CHWs regarding antenatal care. Markers including the number of household visits performed by the CHW assessed behaviour change.<br><br>**Outcome(s):** CHW visits during pregnancy and the postnatal period were significantly higher in the CHW	No details

					iCCM.				group who received supervision.<br>Mothers seen by CHWs who had received supervision demonstrated higher maternal and child health knowledge scores and reported higher exclusive breastfeeding rates. Similarly, HIV-positive mothers were more likely to have disclosed their HIV status to the CHW<br>however, uptake of facility-based interventions were not significantly different.	
Javanparast et al.[46]	The experience of community health workers training in Iran: a qualitative study.	2012	Iran, Middle East	Behvarz	Behvarz are full time employees of the health system. They are selected from her/his own community and work in the 'Village Health House' - the most peripheral health delivery facility in the rural areas of Iran.	91	General focus	**Type:** In-service training<br>**Content:** Updates on new policies and programmes, reinforcement of initial training concepts, and ensuring<br>they are practicing skills learned correctly<br>**Duration:** Variable - ranging	**Outcome measure(s):** Qualitative evaluation e.g. interviews with behvarz.<br><br>**Outcome(s):** Compared to pre-service training, in-service training was viewed unfavourably by the behvarz.<br>They complained	No details

					They have a two year period of training.			from monthly to bi-annually.<br>**Provider:** GPs or other allied health workers<br>**Location:** Rural Health Centres	about "its quality and timing, the infrequency of courses, inadequately qualified trainers who are unfamiliar with the behvarz working environment, the lack of practical sessions and of physical space and training facilities".	
Joos et al.[47]	Evaluation of a mHealth data Quality Intervention to Improve Documentation of Pregnancy Outcomes by Health Surveillance Assistants in Malawi: A cluster RCT.	2016	Malawi, East Africa	Health Surveillance Assistants (HSAs)	HSAs are government trained and paid CHWs.<br><br>They are attached to a local health center and serve approximately 1000 people.<br><br>The scope of their work varies but specific to this project it involved training on the documentation of pregnancies, births, and deaths.	160	Maternal Health	**Type:** Supervision<br>**Content:** The intervention group received SMS messages containing motivational and data quality content.<br>**Duration:** 2-5 SMS messages were sent each week.<br>**Provider:** Mobile based<br>**Location:** NA	**Outcome measure(s):** Change in behaviour, attitudes or practice e.g. Improved recording of pregnancy.<br><br>**Outcome(s):** Improved documentation of pregnancies was observed in both the intervention and control groups.	**Yes** - one-way SMS messages that were sent to HSAs on a regular basis during a 12-month period and reporting on pregnancy outcomes was assessed. Two arms to the study. HSAs in the treatment group received high volume motivational and data quality SMS. HSAs in the control group only received low volume

For peer review only

										motivational SMS.<br><br>Labrique classification:<br>-Provider training and education
Kawasaki et al.[48]	Reactions of community members regarding community health workers' activities as a measure of the impact of a training programme in Amazonas, Brazil.	2015	Brazil, South America	Community Health Workers (CHWs)	Nationwide CHW programme coordinated by the MoH known as Programa dos Agentes Comunitários de Saúde (PACS).<br><br>CHWs are paid.<br><br>Multiple responsibilities including home visits, health promotion, vaccination, record keeping, community meetings.	102	General focus	**Type:** Refresher training course.<br>**Content:** CHWs were trained on facilitating adequate use of health-care services, and health promotion guidance based on the CHW manual published by the MoH plus mention of ad-hoc training<br>**Duration:** Once a month refresher training sessions.<br>**Provider:** An NGO in partnership with the city and state hospital<br>**Location:** In the hospital in the city	**Outcome measure(s):** Change in behaviour, attitudes or practice. Baseline and endline surveys concerning recognition and satisfaction with respect to CHW performance among members of the community were conducted<br><br>**Outcome(s):** Increased awareness of the work of CHWs amongst the community after refresher training courses and better partnership work between supervisors and CHWs was observed.<br><br>The survey also	No details

For peer review only - http://bmjopen.bmj.com/site/about/guidelines.xhtml

									revealed an increase in home visits, greater levels of recognition of CHW functions, and increase levels of satisfaction from community members.	
Kuule et al.[49]	Community Health Volunteers in Primary Healthcare in Rural Uganda: Factors Influencing Performance .	2017	Uganda, East Africa	Community Health Volunteers (CHVs)	CHVs are part of the Village Health Team programme in Uganda.<br><br>The CHVs are trained and maintained by a variety of organisations, including NGOs.<br><br>They are largely unpaid.<br><br>They are expected to engage in a variety of activities including general tasks in all primary health-care core areas e.g. home visits, community information management, health promotion and education, management of	508	Child & Maternal and Reproductive Health	**Type:** Refresher training course and supervision. **Content:** updates on issues such as symptoms of childhood illnesses; key indicators for referrals and how to monitor children for malnutrition. **Duration:** Biannual refresher training sessions and monthly supervisions. **Provider:** Community health nurses **Location:** Hospital setting	**Outcome measure(s):** Change in practice, attitudes or behaviour e.g. attendance at meetings, household follow-up and reporting, immunization coverage.<br><br>**Outcome(s):** Refresher trainings were associated with improved performance, however due to multiple confounding variables they could not be determined to be causative.	No details

					common illnesses, and follow-up of pregnant women.					
Mash et al.[50]	Reflections on the training of counsellors in motivational interviewing for programmes for the prevention of mother to child transmission of HIV in sub-Saharan Africa.	2008	South Africa, Southern Africa	Lay counsellors (LCs)	No details provided	18	HIV	**Type:** Supervision **Content:** Recapping of motivational interviewing techniques **Duration:** Once monthly **Provider:** Four trained action researchers and counsellors **Location:** No details	**Outcome measure(s):** Mixed methods knowledge and skills assessment through assessment of motivational interview techniques and qualitative feedback from supervisors.<br><br>**Outcome(s):** The lay counsellors were not proficient in motivational interviewing despite receiving on going training.<br><br>Qualitative feedback revealed that a lot of time during the on-going training was spent on covering "really basic information".	No details
McLean et al.[51]	Task sharing in rural Haiti: Qualitative assessment of a brief, structured training with	2015	Haiti, Central America	Ajan Santé (community health workers) and Promoteurs (community members	CHWs in this study were largely providing services for HIV/AIDS and cholera prevention prior to the study which	3	Mental Health	**Type:** Supervision **Content:** Recapping of knowledge and skills regarding mental health diagnosis and	**Outcome measure(s):** Mixed methods. Change in practice and behaviour measured through home visits, provision of	No details

For peer review only

	and without apprenticeship supervision for community health workers.			who provide health education through song).	focused on mental health service provision.<br><br>There was a lack of detail regarding financial remuneration and pre-service training.			symptoms, observed practice and details of how to carry out home visits<br>**Duration:** After initial training one week of daily observation by a licensed counselor followed by one further week of supervised sessions.<br>**Provider:** a licensed counselor<br>**Location:** No details	supportive visits and referrals. Qualitative assessment of confidence and satisfaction.<br><br>**Outcome(s):** With supervision there was a greater number of home visits. Qualitative findings support the added value of supervision according to trainees.	
Mengistu et al.[52]	Effect of performance review and clinical mentoring meetings (PRCMM) on recording of community case management by health extension workers in Ethiopia.	2014	Ethiopia, East Africa	Health Extension Workers (HEWs)	HEWs received training using iCCM materials on assessment and treatment of childhood pneumonia, malaria, diarrhea and malnutrition.<br><br>iCCM training was supported by the government and Save the Children (NGO).<br><br>No details on remuneration.<br><br>No details on pre-service education	1175	Child health	**Type:** Supervision<br>**Content:** A review of HEWs records took place on day one and clinical mentoring took place on day two.<br>**Duration:** Twice monthly for two days.<br>**Provider:** Health workers trained as trainers<br>**Location:** A meeting hall in a central town	**Outcome measure(s):** Change in practice, attitudes or behaviour e.g. Improved recording and adherence to iCCM guidelines.<br><br>**Outcome(s):** Supervision improved iCCM performance of HEWs and the authors recommended that as such it should be integrated within the PHC system and given about every six	No details

For peer review only

					levels.				months.	
Miller et al.[53]	Assessment of the impact of quality improvemen t interventions on the quality of sick child-care provided by Health Extension Workers in Ethiopia.	2016	Ethiopia, East Africa	Health Extension Workers (HEWs)	All HEWs are literate women with at least a tenth–grade education, who receive a one–year pre–service training. The pre–service training covers iCCM.<br><br>Following the training, they are recruited as government employees and deployed to work out of health posts at the kebele (sub–district) level.<br><br>There are typically two HEWs working at one health post, which serves approximately 5000 people.	157 (based on an estimate of 1.5 HEWs across 104 health posts)	Child health	**Type:** Refresher training course and supervision **Content:** Refresher training and supportive supervision focussed on reinforcing knowledge and skills learned during the initial iCCM training, carrying out observed visits and checking record keeping before identifying gaps for improvement. **Duration:** Refresher training was a half-day to one-day course eight weeks after intial iCCM training. Supportive supervision was carried out quarterly. **Provider:** Refresher training was provided by iCCM trainer from the district or from	**Outcome measure(s):** Change in behaviour**,** attitudes and practice e.g. number of children correctly managed according to iCCM guidelines.<br><br>**Outcome(s):** Children managed by a HEW who had attended a refresher training course were eight times more likely to be correctly managed, compared to children managed by a HEW who did not. Management by an HEW who received refresher training also significantly increased the odds of correct management**,** whereas the supportive supervision element did not significantly affect the odds of receiving correct care.	No details

For peer review only

								an implementing partner agency. Supervision was provided by implementing partner NGO staff, and sometimes health center staff or woreda health officials.<br>**Location:** Both were held at a local health post		
Mkumbo et al.[54]	Innovation in supervision and support of community health workers for better newborn survival in southern Tanzania.	2014	Tanzania, East Africa	Community Health Volunteers (CHVs).	CHVs were trained to carry out home visits in the first week of life to promote warmth, hygiene and breastfeeding, in order to try improve newborn survival.<br><br>Work in a voluntary capacity.	824	Child health	**Type:** Supervision<br>**Content:** Review of work around newborn checklist, discussion of individual needs<br>**Duration:** Quarterly meetings.<br>**Provider:** Nurse<br>**Location:** Community based visits	**Outcome measure(s):** Change in behaviour, attitudes and practice e.g. number of volunteer–supervisor contacts.<br><br>**Outcome(s):** The community-linked supervision approach resulted in over 50 times more supervision contacts than during the facility-only supervision approach**.**	No details
Msisuka et al.[55]	An evaluation of a refresher training intervention for HIV lay counsellors	2011	Zambia, East Africa	Lay counsellors (LCs)	Lay counsellors are community members recruited by the Zambian Ministry of Health who become certified	25	HIV	**Type:** Refresher training course<br>**Content:** Testing for HIV, counselling and safety. Training	**Outcome measure(s):** Mixed methods. Knowledge and skills assessment was answering true or false questions	No details

For peer review only

	in Chongwe District, Zambia.				after completing a 7-week national training package for psychosocial counseling for HIV. The training package includes a 2-week theoretical component followed by a 5-week supervised practical component. The training covered HIV infection, appropriate values and attitudes for counselors, behaviour change communication, psychosocial support, pre-test and post-test counselling, and professional ethics.			materials were adapted from the National Counseling and Testing Training Curriculum **Duration:** Two-day refresher training course. **Provider:** National trainers for psychosocial Counseling from the National AIDS Counsel **Location:** One central location in the Chongwe district	on a 25-question quiz and testing 10 blood panel samples. An attitudes assessment regarding motivations and obstacles to performance was carried out using a questionnaire.<br><br>**Outcome(s):** Refresher training increased knowledge domains in all areas, particularly in standard precaution and post-exposure prophylaxis.<br><br>52% of LCs responded that periodic opportunities to update their knowledge and skills are crucial to their continued work as LCs.	
Ndima et al.[15]	Supervision of community health workers in Mozambique: a	2015	Mozambique, East Africa	Agentes Polivalentes Elementaires (APEs)	APEs are volunteers, trained by the MoH, They commit to certain terms through a "	18	Child & Maternal and Reproductive Health	**Type:** Supervision **Content:** A checklist was used which covered several areas, including	**Outcome measure(s):** Mixed methods. Qualitative assessment e.g. interviews regarding	No details

	qualitative study of factors influencing motivation and programme implementat ion.				contract" which outlines their right to an allowance or subsidy and free health care at the local primary health centre or dispensary.<br><br>They receive a 4-month residential pre-service training in iCCM and maternal health.			whether APEs had particular commodities available, and if they were completing and recording their duties correctly<br>**Duration:** Monthly supervisions at the community health centre and quarterly supervisions in the community.<br>**Provider:** Qualified nurses attached to a health center<br>**Location:** Mix between health centre and community based	motivation and change in practice e.g. number of home visits and referrals.<br><br>**Outcome(s):** Supervision was irregular and infrequent, affecting APEs motivation. When it did occur, supervision was felt to focus more on fault-finding than being supportive in nature.<br>Supervisors, felt unsupported with high concurrent workloads in health facilities, where they had multiple roles.<br>A lack of resources for supervision activities was identified, and supervisors felt caught up in administrative issues around APE allowances that they were unable to solve. Many supervisors were not trained in	

For peer review only

									providing supportive supervision.	
*Puchalski Ritchie et al.[56]	A knowledge translation intervention to improve tuberculosis care and outcomes in Malawi: a pragmatic cluster randomized controlled trial.	2015	Malawi, East Africa	Health Surveillance Assistants (HSAs)	HSAs are a formal cadre of paid lay health worker. Their roles include provision of outpatient TB care and adherence support. At the time of this study, pre-service training for general HSAs consisted of 10 weeks of in-class training, with approximately 1 day devoted to TB control, transmission, and treatment.<br><br>A subgroup of HSAs, termed TB focus LHWs, receive 2 weeks of additional TB specific training and are responsible for the provision of TB care at the health center	49	TB/HIV	**Type:** In-service training<br>**Content:** Case-based discussions and role playing covering TB transmission, treatment, and consequences of poor adherence; the interaction of TB and HIV; common barriers to adherence and appropriate methods for preventing and addressing non-adherence. There was also training on the use of a clinical support tool.<br>**Duration:** Six on-going training courses lasting for 60-90 minutes over three-months.<br>**Provider:** TB focus LHWs<br>**Location:** Local health centres	**Outcome measure(s):** Change in behaviour, attitudes or practice. Measured through assessing adherence to TB medications and improvements in clinical conditions at the community level.<br><br>**Outcome(s):** There was no different between the control and intervention groups regarding the proportion of treatment successes.	No details

For peer review only

					level. TB focus LHWs recruit and train general LHWs to assist with TB care.					
*Puchalski Ritchie et al.[57]	Lay Health Workers experience of a tailored knowledge translation intervention to improve job skills and knowledge: a qualitative study in Zomba district Malawi.	2016	Malawi, East Africa	Health Surveillance Assistants (HSAs)	See description provided above.	36	TB/HIV	**Type:** In-service training **Content:** Case based role playing and discussions covering topics such as TB transmission and natural history, the interaction of TB and HIV, TB treatment including side-effects and their management, common barriers to adherence, consequences of poor treatment adherence, and approaches to preventing and addressing poor adherence **Duration:** Six on-going training courses lasting for 60-90 minutes over three-months. **Provider:** TB focus LHWs **Location:** Local	**Outcome measure(s):** Qualitative assessment. Interviews with CHWs regarding perceived improvement in knowledge and skills and ability to perform their roles. **Outcome(s):** Generally the in-service training was well received. HSAs reported increased TB, HIV, and job-specific knowledge; improved clinical skills; and increased confidence and satisfaction with their work. Suggestions for improvement were less consistent across participants, but included: increasing the duration of the	No details

For peer review only

								health centres	training, changing to an off-site venue, providing stipends or refreshments as incentives, and adding HIV and drug dosing content	
**Rabbani et al.[58]	Health workers' perspectives, knowledge and skills regarding community care management of childhood diahorrea and pneumonia: a qualitative inquiry for an implementation research project "Nigraan" in District Badin, Sindh, Pakistan.	2016	Pakistan, South Asia	Lady Health Workers (LHWs)	LHWs provide preventive and basic curative maternal, newborn and under five child health (MNCH) services in their catchment area. LHWs are salaried staff, recognized by the government and are preferably married and educated (minimum eight years of schooling). They mostly reside in the area where they serve. An LHW serves approximately 100– 150 households, representing an average population of 1000.	108	Child Health	**Type:** Supervision **Content:** Supervisory visits involve the LHW being accompanied during their home visits, where their supervisor guides them and addresses their concerns **Duration:** Twice monthly. **Provider:** Lady Health Supervisors **Location:** Community based	**Outcome measure(s):** Mixed methods. Knowledge and skills assessment regarding management plus qualitative perceptions of the supervision.<br><br>**Outcome(s):** LHWs considered adequate supervision and the presence of LHSs during household visits as a factor facilitating their performance. LHWs did not have a preference for written or verbal feedback, but supervisors considered written individual feedback to LHWs to be more useful than group and verbal feedback.	No details

For peer review only

					The LHW also works from her home, where she is encouraged to have a portion of her home designated as a " Health House".<br><br>The are supervised by Lady Health Supervisors (LHSs). LHSs are attached to the first level care facility (FLCF) and are responsible for on-going supervision and monitoring of LHWs. LHSs are female health workers aged 22– 45, residing locally with a good educational background and have several years' experience as a LHW. Salaries range from $160-180/month. Each LHS supervises approximately 15– 25 LHWs.					

For peer review only

**Rabbani et al.[59]	Inspiring health worker motivation with supportive supervision: a survey of lady health supervisor motivating factors in rural Pakistan.	2016	Pakistan, South Asia	Lady Health Supervisors (LHSs)	See description of LHSs provided above.	29	Child Health	**Type:** Supervision **Content:** Supervisory visits involve the LHW being accompanied during their home visits, where their supervisor guides them and addresses their concerns **Duration:** Twice monthly **Provider:** LHSs **Location:** Community based	**Outcome measure(s):** Qualitative measures e.g. motivation following supervision. **Outcome(s):** Lady health supervisors are motivated by both their role in providing supportive supervision to lady health workers and by the supervisory support received from their coordinators and managers.	Mobile phones were provided to improve communicatio n and coordination between LHSs and LHWs regarding case detection, tracking, management, and follow-up. Labrique classification: -Provider to provider communicatio n
Roberton et al.[60]	Initial experiences and innovations in supervising community health workers for maternal, newborn, and childhealth in Morogoro region,	2015	Tanzania, East Africa	Community Health Workers (CHWs)	CHWs are volunteers. Their role includes identifying pregnancies, conducting routine home visits to antenatal and postpartum women and facilitating group-based discussion sessions in the community.	228	Maternal and Child Health	**Type:** Supervision **Content:** Different supervisors have different roles. Facility based supervisors responsibilities include providing technical support to CHWs to facilitate community mapping and household census,	**Outcome measure(s):** Mixed methods. Qualitative interviews assessing CHWs experiences and change in behaviour, attitudes or practice measured through survey data recording frequency and content of CHW supervision.	No details

	Tanzania.				The topics of these discussions include antenatal care, danger signs, birth preparedness, maternal and child nutrition, postpartum and newborn care, family planning, and HIV/AIDS.<br><br>CHWs are required to be residents of the village, over age 18, role models for maternal and child health in their community, and preferably with at least form four level of schooling.<br><br>CHWs self nominate themselves prior to village governments nominating their top candidates. Selection of of CHWs was finalized at			MoH supervisors used supervisions to provide CHWs with working tools and stipends whereas village leaders work at a local level for advocacy, support and community awareness<br>**Duration:** Once monthly from facility based supervisors and quarterly from MoH teams and village leaders<br>**Provider:** A mixed provider model including facility health workers trained in supportive supervision through a 2-week " Community MNCH Supervisor' s Training" programme, village leaders and<br>**Location:** Mixture between primary health clinic and in the field	**Outcome(s):** CHWs value supervision and appreciate the sense of legitimacy that arises when supervisors visit them in their village. Village leaders and district staff are engaged and committed to supporting CHWs. Despite these successes, facility-based supervisors visit CHWs in their village an average of only once every 2.8 months, CHWs and supervisors still see supervision primarily as an opportunity to check reports, and meetings with district staff are infrequent and not well scheduled.	

For peer review only - http://bmjopen.bmj.com/site/about/guidelines.xhtml

					village meetings.					
Rowe et al.[61]	Longitudinal analysis of community health workers' adherence to treatment guidelines, Siaya, Kenya, 1997-2002.	2007	Kenya, East Africa	Community Health Workers (CHWs)	CHWs were trained by CARE, an NGO.<br><br>Their role was to assess, diagnose and treat children under age 5 years according to the CARE Management of the Sick Child (MSC) guidelines, a simplified version of the WHO/ UNICEF iCCM guidelines.	114	Child Health	**Type:** Refresher training course **Content:** Knowledge reviews followed by working on weaknesses in CHW clinical skills that were identified by performance assessments and practical sessions in small groups **Duration:** Two three-month blocks of refresher training. **Provider:** No details **Location:** No details	**Outcome measure(s):** Change in behaviour, attitudes or practice e.g. correct referrals and management of sick children.<br><br>**Outcome(s):** The study revealed that immediately after the first refresher training, the mean adherence level of CHWs to the guidelines for managing sick children improved for patients with a severe illness, but worsened for patients without severe illness. Adherence scores declined rapidly during the 6 months after the second refresher training. The authors concluded that the first refresher was partially effective but the second refresher had an effect contrary to that intended, and patient	No details

For peer review

									characteristics had a strong influence on adherence patterns.	
Singh et al.[13]	Supportive supervision for volunteers to deliver reproductive health education: a cluster randomized trial.	2016	Uganda, East Africa	Community Health Volunteers (CHVs) and Community Health Workers (CHWs)	CHVs role in this context was to offer preventative reproductive health care. They had "relatively short training" and they volunteer 5–10 h per week, receiving little or no remuneration and poor supervision after an initial training period.<br><br>Groups of CHVs are known as Village Health Teams and are often maintained by various NGOs<br><br>The CHWs were younger demographic with higher basic education. Trained for 6 months to 2 years as full-time members of the health system.	82	General focus	**Type:** Supervision **Content:** The supervision sessions involved accompaniment on a home visit and topics covered were those from previous training sessions such as encouraging birth at a health facility or danger signs in pregnancy **Duration:** Monthly training lasting for between two to three hours per month. **Provider:** CHWs **Location:** In the field (home visits)	**Outcome measure(s):** Change in behaviour, attitudes or practice e.g. immunizations, breastfeeding, number of installed tippy taps for hand washing assessed at the household level.<br><br>**Outcome(s):** Overall this study demonstrated an increase in desired behaviors in both the intervention and control arms over the study period. Both arms showed high retention rates of CHVs. At 1 year follow-up there was a significantly higher prevalence of installed and functioning tippy taps for hand washing in the intervention villages than control villages. All outcome and	No details

For peer review only

									process measures related to home-visits to homes with pregnant women and newborn babies favored the intervention villages.	
Sylla et al.[62]	Low level educated community health workers training: a strategy to improve children access to acute respiratory treatment in Senegal	2007	Senegal, West Africa	Les agents de santé communautaire (ASC)	ASCs are volunteers serving their communities. They offer their services within health huts. They are recruited by the government and are typically have at least a primary level of education and are able to read and write in French.<br><br>In this programme ASCs received a 3-day preservice training course using the WHO guidelines for Acute Respiratory Illness.	107	Child Health	**Type:** In-service training **Content:** Recapping material from the WHO guidelines to assess & manage acute respiratory illness (ARI) **Duration:** Once a month **Provider:** Head nurse **Location:** Health centres	**Outcome measure(s):** Change in behaviour, attitudes or practice.<br><br>**Outcome(s):** ASCs who were trained and supported with follow-up could help provide care to children with ARI in the community by following the WHO guidelines for ARI recognition and management, however given that 28% of severe pneumonia cases were misclassified as pneumonia it would be important to emphasize the recognition of danger signs and the follow-up of severe cases.	No details
Talukder	In a rural	2016	Banglades	Traditional	TBAs are also	N/A –	Child	**Type:** Supervision	**Outcome**	No details

For peer review only

et al.[63]	area of Bangladesh, traditional birth attendant training improved early infant feeding practices: a pragmatic cluster randomized trial.		h, South Asia	birth attendants (TBAs) / Community Volunteers (CVs)	known as 'skilled birth attendants' and are government trained. There are approximately 7500 working across Bangladesh. Their roles are include assisting deliveries and advising mothers on breastfeeding.<br><br>CVs are community based volunteers such as relatives, friends or neighbours. No information was provided on their exact roles or training.	randomisation done at district level	health	**Content:** Field supervisors checked on breastfeeding activities in the community<br>**Duration:** Once a week supervision sessions.<br>**Provider:** Field supervisors<br>**Location:** In the field	**measure(s):** Change in behaviour, attitudes or practice e.g. number of home visits, initiation of breastfeeding<br><br>**Outcome(s):** Although outcome measures, such as rate of breast feeding and avoidance of pre-lacteal feeds, improved in both groups, there was no significant difference between outcome measures in the group that had received just training compared to training plus supervision	
Vu Henry et al.[14]	Enhancing the Supervision of Community Health Workers With WhatsApp Mobile Messaging: Qualitative Findings From 2	2016	Kenya, East Africa	Community Health Volunteers / Community Health Extension Workers (CHEWs)	CHVs are volunteer Community Health workers. No further details were provided on specific cadre roles.<br><br>CHEWs are the CHV supervisors.	25	General focus	**Type:** Supervision<br>**Content:** Messages were sent between CHVs and CHEWs regarding assessing childhood development Milestones.<br>**Duration:** Continuous supportive supervision over a	**Outcome measure(s):** Qualitative analysis of WhatsApp messages.<br><br>**Outcome(s):** The thematic analysis revealed that most of the content related to creating a social environment, sharing	Yes – WhatsApp groups were created between supervisors and CHWs to support supervision, professional development, and team building.

For peer review only - http://bmjopen.bmj.com/site/about/guidelines.xhtml

	Low-Resource Settings in Kenya.							period of six months via WhatsApp. **Provider:** Through a communication group between CHWs and their supervisors installed on a mobile phone **Location:** Not applicable (mobile based)	communication and information, or promoting quality of services.	Labrique classification categories:<br><br>-Provider to provider communicatio n<br>-Provider training and education<br>-Human Resource Management
Vallières et al.[64]	There's No App for That: Assessing the Impact of mHealth on the Supervision, Motivation, Engagement, and Satisfaction of Community Health Workers in Sierra Leone.	2016	Sierra Leone, West Africa	Community Health Workers (CHWs)	CHWs in this study were trained by World Vision Ireland' s Access to Infant and Maternal Health programme.<br><br>Recruitment was done in accordance with the Policy for Community Health Workers in Sierra Leone published by the MoH.<br><br>CHWs in this model are volunteers, undergo a minimum 10-day basic training	292	Maternal and child health	**Type:** Supervision **Content:** The MOTECH suite app allowed CHWs to register pregnant women and their children, alert CHWs when household visits are overdue, allow CHWs to make referrals to their affi liated PHU, and collect household data during household visits. **Duration:** 6-months **Provider:** MOTECH suite **Location:** Mobile based application	**Outcome measure(s):** Change in behaviour, attitudes or practice measured through self-reported measures of work engagement and job satisfaction.<br><br>**Outcome(s):** There was no differences between the perceived supervision and motivation across the different groups of CHWs over time with the introduction of the MOTECH Suite as a human resource management tool. Furthermore, there	**Yes** - assessed the use of the Mobile Technology for Community Health (MOTECH) Suite application on the perceived organizational factors of a CHW programme. The MOTECH suite allows CHWs to "register pregnant women and their children …alert CHWs when

For peer review only

					course and be a resident of the village.<br><br>They serve between 100-500 people.				was no significant change in the self-reported measures of work engagement and job satisfaction across each of the intervention groups over time.	household visits are overdue, allow CHWs to make referrals to their local health unit and collect household data".<br><br>Labrique categories:<br>-Registries or vital event tracking<br>-Data collection and reporting<br>-Provider workplanning and scheduling<br>-Provider to provider communication<br>-Human Resource Management
Zeitz et al.[65]	Community health worker competency in managing acute respiratory	1993	Bolivia, South America	Communtiy Health Workers (CHWs)	CHWs in this study were recruited and managed by three different NGOs.<br><br>They were all	80	Child Health	**Type:** Refresher training course<br>**Content:** Knowledge and skills pertaining to the recognition of acute respiratory	**Outcome measure(s):** Knowledge and skills assessment using a pre- and post-intervention test.	No details

For peer review only

	infections of childhood in Bolivia.				volunteers with additional jobs with many working in agriculture. Four-fifths of the members of all three groups were literate and able to count.<br><br>They had all received variable training based on the WHO acute respiratory infections guidelines (*some of which were out-dated*).			infection in children using the results of a pre-course evaluation.<br>**Duration:** One-day refresher course lasting for eight hours.<br>**Provider:** Physicians, nurses, and auxiliary health workers who were routinely involved with training CHWs in ARI management.<br>**Location:** No details	**Outcome(s):** Improvements were seen across the pre and post test assessments following refresher training and statistically significant improvements were observed in key domains including identification of danger signs, acute respiratory tract infection classification and treatment.	

Legend. A table outlining key details from the studies included within the final scoping review.

Key

*Two separate analyses from the same study. One study focused on the number of HIV and TB cases correctly managed following a knowledge translation intervention, the other focused on a qualitative evaluation of the same intervention.

**Two separate analyses from the same study. One study focused on a qualitative evaluation of Lady Health Supervisors feelings of motivation following provision of supervisor, the other focussed on a mixed methods assessment of Lady Health Workers regarding management of childhood diarrhoea and pneumonia following supervision plus qualitative perceptions of the supervision.

For peer review only

For peer review only

PRISMA 2009 Checklist

Section/topic	#	Checklist item	Reported on page #
**TITLE**			
Title	1	Identify the report as a systematic review, meta-analysis, or both.	1,2 and 7
**ABSTRACT**			
Structured summary	2	Provide a structured summary including, as applicable: background; objectives; data sources; study eligibility criteria, participants, and interventions; study appraisal and synthesis methods; results; limitations; conclusions and implications of key findings; systematic review registration number.	2
**INTRODUCTION**			
Rationale	3	Describe the rationale for the review in the context of what is already known.	5-6
Objectives	4	Provide an explicit statement of questions being addressed with reference to participants, interventions, comparisons, outcomes, and study design (PICOS).	6 & 10-11
**METHODS**			
Protocol and registration	5	Indicate if a review protocol exists, if and where it can be accessed (e.g., Web address), and, if available, provide registration information including registration number.	NA (See reason on page 7)
Eligibility criteria	6	Specify study characteristics (e.g., PICOS, length of follow-up) and report characteristics (e.g., years considered, language, publication status) used as criteria for eligibility, giving rationale.	8-9
Information sources	7	Describe all information sources (e.g., databases with dates of coverage, contact with study authors to identify additional studies) in the search and date last searched.	8-9 & 12
Search	8	Present full electronic search strategy for at least one database, including any limits used, such that it could be repeated.	Supplementary material 1-14
Study selection	9	State the process for selecting studies (i.e., screening, eligibility, included in systematic review, and, if applicable, included in the meta-analysis).	9-10
Data collection process	10	Describe method of data extraction from reports (e.g., piloted forms, independently, in duplicate) and any processes for obtaining and confirming data from investigators.	12
Data items	11	List and define all variables for which data were sought (e.g., PICOS, funding sources) and any assumptions and simplifications made.	12
Risk of bias in individual studies	12	Describe methods used for assessing risk of bias of individual studies (including specification of whether this was done at the study or outcome level), and how this information is to be used in any data synthesis.	N/A (see comment in strengths and limitations

For peer review only

PRISMA 2009 Checklist

			section regarding scoping reviews on pages 7 & 22)
Summary measures	13	State the principal summary measures (e.g., risk ratio, difference in means).	12
Synthesis of results	14	Describe the methods of handling data and combining results of studies, if done, including measures of consistency (e.g., I^2) for each meta-analysis.	N/A

Section/topic	#	Checklist item	Reported on page #
Risk of bias across studies	15	Specify any assessment of risk of bias that may affect the cumulative evidence (e.g., publication bias, selective reporting within studies).	N/A (see comment in strengths and limitations section on page 7)
Additional analyses	16	Describe methods of additional analyses (e.g., sensitivity or subgroup analyses, meta-regression), if done, indicating which were pre-specified.	N/A
**RESULTS**			
Study selection	17	Give numbers of studies screened, assessed for eligibility, and included in the review, with reasons for exclusions at each stage, ideally with a flow diagram.	Page 14 and PRISMA diagram
Study characteristics	18	For each study, present characteristics for which data were extracted (e.g., study size, PICOS, follow-up period) and provide the citations.	Supplementary material Table 3
Risk of bias within studies	19	Present data on risk of bias of each study and, if available, any outcome level assessment (see item 12).	N/A (see comment in strengths and limitations section on page 7 and 22)

For peer review only

PRISMA 2009 Checklist

Results of individual studies	20	For all outcomes considered (benefits or harms), present, for each study: (a) simple summary data for each intervention group (b) effect estimates and confidence intervals, ideally with a forest plot.	Supplementary material Table 3
Synthesis of results	21	Present results of each meta-analysis done, including confidence intervals and measures of consistency.	N/A
Risk of bias across studies	22	Present results of any assessment of risk of bias across studies (see Item 15).	N/A
Additional analysis	23	Give results of additional analyses, if done (e.g., sensitivity or subgroup analyses, meta-regression [see Item 16]).	N/A
**DISCUSSION**			
Summary of evidence	24	Summarize the main findings including the strength of evidence for each main outcome; consider their relevance to key groups (e.g., healthcare providers, users, and policy makers).	Supplementary material Table 3 and discussion page 17-22
Limitations	25	Discuss limitations at study and outcome level (e.g., risk of bias), and at review-level (e.g., incomplete retrieval of identified research, reporting bias).	22
Conclusions	26	Provide a general interpretation of the results in the context of other evidence, and implications for future research.	23
**FUNDING**			
Funding	27	Describe sources of funding for the systematic review and other support (e.g., supply of data); role of funders for the systematic review.	3

From: Moher D, Liberati A, Tetzlaff J, Altman DG, The PRISMA Group (2009). Preferred Reporting Items for Systematic Reviews and Meta-Analyses: The PRISMA Statement. PLoS Med 6(7): e1000097. doi:10.1371/journal.pmed1000097

For peer review only
